# Tandem Cu-catalyzed ketenimine formation and intramolecular nucleophile capture: Synthesis of 1,2-dihydro-2-iminoquinolines from 1-(*o*-acetamidophenyl)propargyl alcohols

**DOI:** 10.3762/bjoc.10.125

**Published:** 2014-05-28

**Authors:** Gadi Ranjith Kumar, Yalla Kiran Kumar, Ruchir Kant, Maddi Sridhar Reddy

**Affiliations:** 1 Medicinal & Process Chemistry Division, CSIR-Central Drug Research Institute, BS-10/1, Sector 10, Jankipuram extension, Sitapur Road, P.O. Box 173, Lucknow 226031, India; 2 Academy of Scientific and Innovative Research, New Delhi 110001, India; 3 Molecular & Structural Biology Division, CSIR-Central Drug Research Institute,BS-10/1, Sector 10, Jankipuram extension, Sitapur Road, P.O. Box 173, Lucknow 226031, India

**Keywords:** alkyne, azide, cycloaddition, cyclization, quinoline

## Abstract

The copper-catalyzed ketenimine formation reaction of 1-(*o*-acetamidophenyl)propargyl alcohols with various sulfonyl azides is found to undergo a concomitant intramolecular nucleophile attack to generate 1,2-dihydro-2-iminoquinolines after aromatization (via elimination of acetyl and hydroxy groups) and tautomerization. The reaction produces 4-substituted and 3,4-unsubstituted title compounds in moderate to good yields under mild reaction conditions.

## Introduction

The synthesis of *N*-sulfonylketenimines via CuAAC (copper-catalyzed azide–alkyne cycloaddition) between terminal alkynes and sulfonyl azides has staged for a conceptually novel class of approaches for the synthesis of various nitrogen-containing cyclic and acyclic motifs of pharmaceutical and material interests [1–23]. After its discovery by Chang and co-workers [1–6], various groups (including the discoverers) have utilized the reaction for the synthesis of diverse chemical compounds by trapping the thus formed ketenimine inter- and intramolecularly. Various multicomponent reactions using external nucleophiles like H_2_O, alcohols, amines, imines, etc., have been reported while very few recent methods [16–23], including ours [16], disclosed the utilization of internal nucleophiles for trapping the ketenimines successfully. In continuation of our interest in the functionalization of alkynes through electrophilic activation [24–29], we herein report a synthesis of 1,2-dihydro-2-iminoquinolines (tauromerized form of 2-sulfonylaminoquinolines) from 1-(*o*-acetamidophenyl)propargyl alcohols via copper-catalyzed ketenimine formation with various sulfonyl azides.

2-Aminoquinolines and their derivatives have been found to be important constituents in pharmaceutical chemistry [30–42] and also found to play a crucial role in molecular recognition processes [43–46]. Due to its vast-spread importance, several methods have been developed to construct this useful framework. Apart from the classical Chichibabin reaction [47], there has been considerable interest shown for the synthesis of these compounds with varying substitution patterns [48–64]. Most of the methods used presynthesized quinolines for the 2-amination via either creating initially a leaving group and its subsequent substitution or via activation of the nitrogen atom and direct amination at C-2 while few methods afforded the direct 2-aminoquinoline synthesis from the acyclic precursors.

**Scheme 1:**
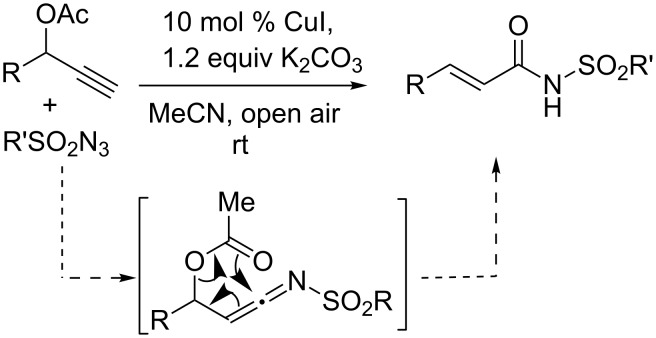
Synthesis of unsaturated amides via ketenimine formation.

Most recently, we have disclosed a method for the synthesis of *E*-α,β-unsaturated amides from propargyl acetates via copper catalyzed ketenimine formation with sulfonyl azides (Scheme 1) [16]. The reaction was thought to proceed through intramolecular nucleophilic attack on the in situ generated ketenimine. We became curious to know the fate of the reaction in the presence of another internal nucleophile in the same distance (6-membered) as a competitor.

## Results and Discussion

**Scheme 2:**
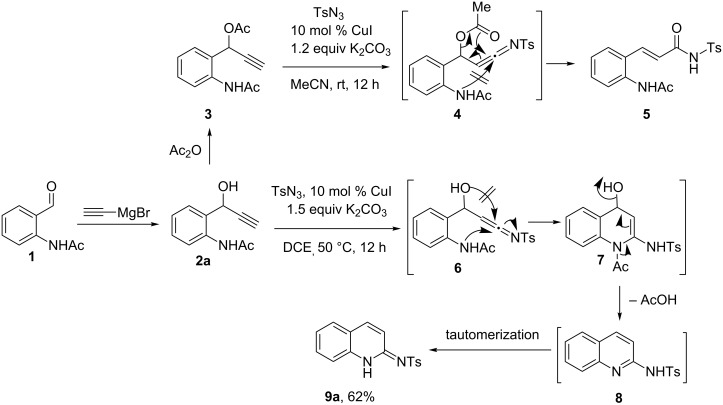
Intramolecular nucleophilic capture by ketenimines.

Thus, we synthesized a substrate **3** (from **1** via **2a**) through Grignard reaction followed by acetylation (Scheme 2). Substrate **3** contains two nucleophiles in the form of acetyl and acetamide groups at equal distance. When we treated **3** with similar reaction conditions (TsN_3_, 10 mol % CuI, 1.2 equiv K_2_CO_3_, 0.1 M CH_3_CN) as used in the previous method, surprisingly, only α,β-unsaturated amide **5** was obtained as a single product through acetyl migration on the intermediate **4**. No trace of product through acetamide attack was obtained, which was probably due to the weaker nucleophilic nature of the latter nucleophile. We then wanted to test the method on **2a** which contains a hydroxy group as a nucleophile for a relatively strained 4-membered attack and the acetamide group for the 6-membered attack. In our earlier study, the hydroxy group was found to be a less effective nucleophile compared to the acetyl group in such a reaction, as the earlier requires higher energy for its strained 4-membered cyclization. In light of this, we speculated that the acetamide group might have more chances for the cyclization in the tentative intermediate **6**. As expected, the reaction on **2a** proceeded through a 6-membered cyclization to form **7** which underwent aromatization via elimination of acetic acid to produce **8**. Product **8** exists in tautomerized form of **9** (vide infra, X-ray structure of **9j**). In fact, an elegant synthesis of such compounds was earlier achieved by Wang et al. through three component coupling reaction under similar conditions [13]. The method was shown to be not working for the synthesis of 4-unsubstituted adducts as the 2-aminobenzaldehyde was found to be unreactive in the given conditions and, moreover, the reaction is not possible with acetylene as the alkyne partner to produce 3,4-unsubstituted adducts. We herein report the intramolecular version of Wang's protocol for the synthesis of differently substituted products.

**Scheme 3:**
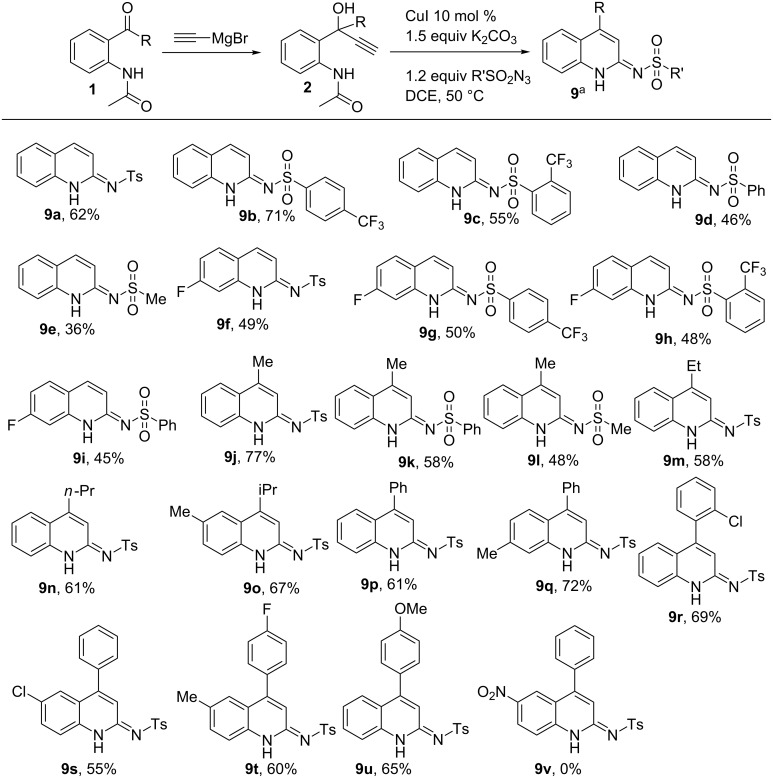
Synthesis of 1,2-dihydro-2-tosyliminoquinolines. Reaction conditions: Sulfonyl azide (1.2 mmol), **2** (1.0 mmol), base (1.5 mmol), CuI (0.1 mmol), DCE (5 mL, 0.2 M solution), 50 °C, open air. ^a^Isolated yield.

To explore the practicality of the above reaction, a series of starting materials were selected with the variation in substitution on propargyl alcohols as well as sulfonyl azides. It was found that the cascade process was applicable for a wide range of substrates providing the dihydroquinoline derivatives **9** in yields ranging from 36% to 77% (Scheme 3). Initially, we screened 2-propargyl alcohols prepared from aminobenzaldehydes as they were unreactive in Wang's protocol. Thus, **2a** was treated with various sulfonyl azides. Trifluoromethanesulfonyl azides reacted similar to toluenesulfonyl azide to afford the products **9b** and **9c** in 71% and 55% yields. The lower yield in case of **9c** can be attributed to the steric hindrance created by the bulky triflruoromethyl group at the ortho position. Benzene- and methanesulfonyl azides produced the corresponding products (**9d** and **9e**) in somewhat lower yields (46% and 36%, respectively). Fluorinated 2^o^-propargyl alcohol **2b** was then reacted with various aryl sulfonyl azides to get the corresponding products **9f–i** in yields ranging from 45–50%.

Next, 3^o^-propargyl alcohols prepared from aminoacetophenones and aminobenzophenones were subjected to the title reaction for the synthesis of 4-methyl/phenyl-1,2-dihydro-2-iminoquinolines. It was found that these 3^o^-propargyl alcohols were more productive compared to the above 2^o^-propargyl alcohols. Thus **2c–f** were subjected to the standard reaction conditions with various sulfonyl azides to get the corresponding 4-alkyl-substituted quinoline derivatives **9j-o** in 48–77% yields. In further exploration, 4-phenyl-substituted adducts **9p** and **9q** were synthesized from **2g** and **2h** in 61% and 72% yields, respectively. Halogen groups (chloro and fluoro) on the core part as well as the pendant phenyl ring survived well in the reaction to produce the products (**9r–t**) in yields ranging from 55% to 69%. Electron rich phenyl substrate **2l** produced the corresponding product **9u** in 65% yield while electron poor substrate **2m** was found to be unreactive in the reaction.

**Figure 1:**
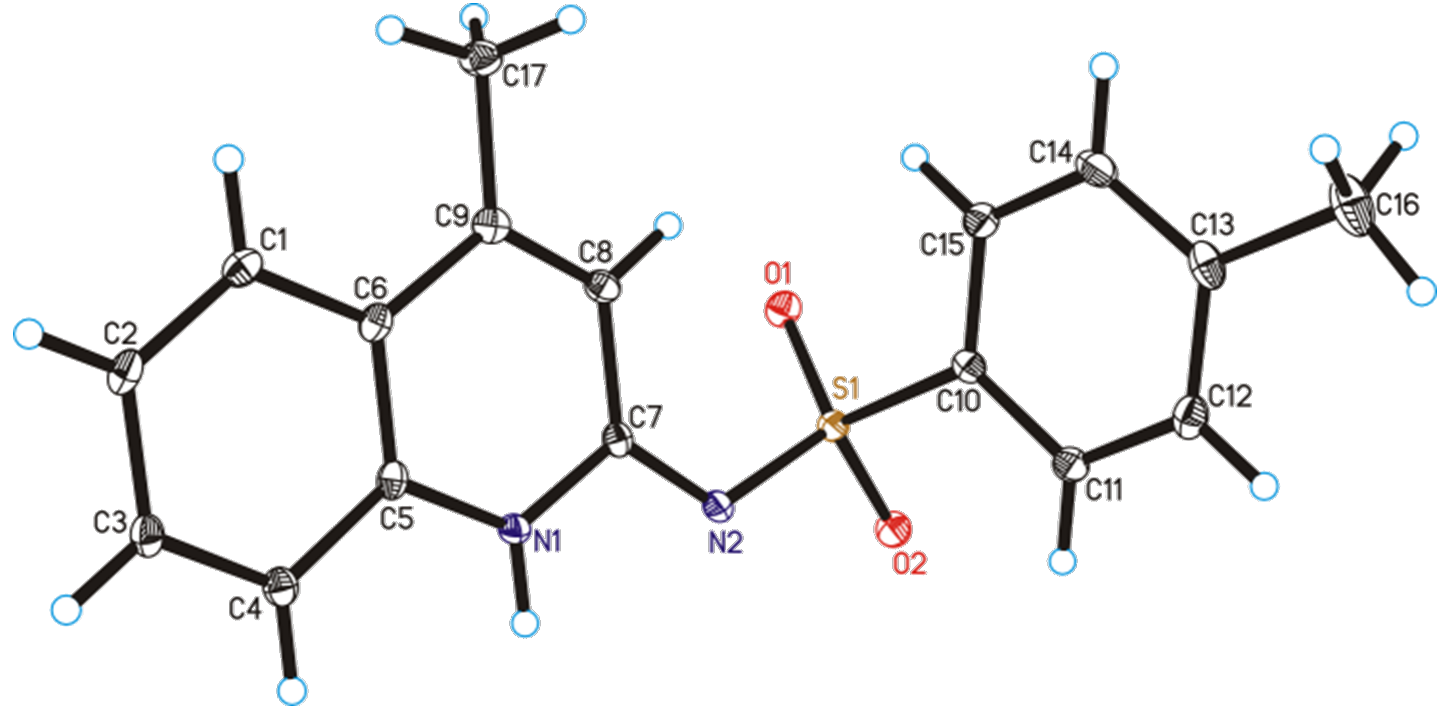
X-ray Structure of **9j** (CCDC number 971729).

The structures of the products were confirmed by ^1^H NMR, ^13^C NMR and High Resolution Mass Spectrometry (HRMS). X-ray crystallography of one of the compounds, **9j**, gave unambiguous structure confirmation (Figure 1). See also Supporting Information File 1, pages S49–S57 and Supporting Information File 2.

## Conclusion

We have described a method for the synthesis of 1,2-dihydro-2-iminoquinolines, tautomerized products of 2-tosylaminoquinolines, from 1-(*o*-acetamidophenyl)propargyl alcohols through copper-catalyzed ketenimine formation with various sulfonyl azides and concomitant intramolecular nucleophilic attack. The method is overall an intramolecular version of the Wang's protocol for the synthesis of differently substituted 1,2-dihydro-2-tosyliminoquinolines which cannot be synthesized by the latter method. The reaction was performed under mild conditions to obtain the products in good yields and with a broad spectrum of substitution patterns.

## Experimental

General information: All reagents and solvents were purchased from commercial sources and used without purification. NMR spectra were recorded with a 300 or 400 MHz spectrometer for ^1^H NMR, 75 or 100 MHz for ^13^C NMR spectroscopy. Chemical shifts are reported relative to tetramethylsilane in CDCl_3_ or to residual signals of undeuterasted solvent CDCl_3_/DMSO-*d*_6_ for ^1^H and ^13^C NMR spectroscopy. Multiplicities are reported as follows: singlet (s), doublet (d), broad singlet (bs), doublet of doublets (dd), doublet of triplets (dt), triplet (t), quartet (q), multiplet (m). HRMS spectra were recorded by using a QTof mass spectrometer. Column chromatography was performed with silica gel (100–200 mesh) as the stationary phase. All reactions were monitored by using TLC. The purity and characterization of compounds were further established by using HRMS.

**General procedure for the synthesis of 9 from 2 taking synthesis of 9a as an example:** To the substrate **2a** (190 mg, 1 mmol) dissolved in dichloroethane (5 mL) was added tosyl azide (236 mg, 1.2 mmol), copper iodide (19 mg, 0.1 mmol), and K_2_CO_3_ (207 mg, 1.5 mmol). The mixture was stirred at 50 °C for 12 h. The reaction mixture was then added water (5 mL) followed by brine solution (5 mL) and extracted with ethyl acetate (3 × 10 mL). The combined extracts were dried over Na_2_SO_4_, filtered and concentrated. The crude solid product was purified by column chromatography (silicagel, 20–30% EtOAc in hexanes) to get the pure product **9** (180 mg, 62% yield).

## Supporting Information

File 1 Analytical data.

File 2 X-ray structure.
