# Supplementary material for: Tandem Cu-catalyzed ketenimine formation and intramolecular nucleophile capture: Synthesis of 1,2-dihydro-2-iminoquinolines from 1-(o-acetamidophenyl)propargyl alcohols
Source: Beilstein J Org Chem. 2014 May 28;10:1255–60. doi: 10.3762/bjoc.10.125 (PMC4077525; doi:10.3762/bjoc.10.125)
Supplement: File 1 — Analytical data. [file Beilstein_J_Org_Chem-10-1255-s001.pdf]

Supporting Information

for

Tandem Cu-catalyzed ketenimine formation and intramolecular nucleophile capture: Synthesis of 1,2-dihydro-2-iminoquinolines from 1-(*o*-acetamidophenyl)propargyl alcohols

Gadi Ranjith Kumar[1,2,†], Yalla Kiran Kumar[2,†], Ruchir Kant[3]
and Maddi Sridhar Reddy*[1,2,§]

Address: [1]Medicinal & Process Chemistry Division, CSIR-Central Drug Research Institute, BS-10/1, Sector 10, Jankipuram extension, Sitapur Road, P.O. Box 173, Lucknow 226031, India, [2]Academy of Scientific and Innovative Research, New Delhi 110001, India and [3]Molecular & Structural Biology Division, CSIR-Central Drug Research Institute, BS-10/1, Sector 10, Jankipuram extension, Sitapur Road, P.O. Box 173, Lucknow 226031, India

Email: Maddi Sridhar Reddy* - msreddy@cdri.res.in

*Corresponding author

[†]Contributed equally.

[§]Tel +91-7897582222.

Analytical data

Table of contents

Spectroscopic data for all the compounds…..……………………..……..…...S2–S12

References...S12

Copies of ^{1}H and ^{13}C NMR spectra…..……………..………………………......S13–S48

Detailed write up for the X-ray structure of **9j**......……………………….......S49–S57

Spectroscopic data for all the compounds

***N*-(2-(1-Hydroxyprop-2-ynyl)phenyl)acetamide (2a):** 95% yield (180 mg); white solid; mp 110-112 ^{o}C; R_f = 0.20 (SiO_2, 30% EtOAc/Hexanes); ^{1}H NMR (400 MHz, DMSO-d_6) δ 9.29 (s, 1H), 7.60-7.56 (m, 2H), 7.31-7.27 (m, 1H), 7.18 (t, *J* = 7.3 Hz, 1H), 6.20 (d, *J* = 5.6 Hz, 1H), 5.57 (dd, *J* = 5.6, 2.2 Hz, 1H), 3.47 (d, *J* = 2.2 Hz, 1H), 2.07 (s, 3H); ^{13}C NMR (100 MHz, DMSO-d_6) δ 168.7, 135.9, 134.0, 128.5, 127.6, 125.1, 84.9, 76.2, 60.1, 24.1; IR (cm^{-1}) v 3390, 3021, 2401,1674, 1216; HRMS (ESI-TOF) calcd for $C_{11}H_{11}NO$ [M + H –H2O]$^{+}$ 172.0762, found 172.0739.

***N*-(5-Fluoro-2-(1-hydroxyprop-2-ynyl)phenyl)acetamide (2b):** 82% yield (170 mg); white solid; mp 130-132 ^{o}C; R_f = 0.20 (SiO_2, 30% EtOAc/Hexanes); ^{1}H NMR (400 MHz, DMSO-d_6) δ 9.34 (s, 1H), 7.61-7.55 (m, 2H), 7.03-6.96 (m, 1H), 6.33 (d, *J* = 5.6 Hz, 1H), 5.61 (dd, *J* = 5.6, 2.2 Hz, 1H), 3.52 (d, *J* = 2.2 Hz, 1H), 2.09 (s, 3H); ^{13}C NMR (100 MHz, DMSO-d_6) δ 168.9, 163.0, 160.6, 137.7, 137.6, 129.3, 129.2, 129.1, 111.3, 111.1, 110.9, 110.7, 84.5, 76.6, 59.7, 24.3; IR (cm^{-1}) v 3684, 3019, 2400, 1605, 1215; HRMS (ESI-TOF) calcd for $C_{11}H_{10}FNO$ [M + H – H2O]+ 190.0668, found 190.0644.

***N*-(2-(2-Hydroxybut-3-yn-2-yl)phenyl)acetamide (2c)** [1]**:** 79% yield (160mg); white solid; mp 133-135 ^{o}C; R_f = 0.25 (SiO_2, 30% EtOAc/Hexanes); ^{1}H NMR (400 MHz, $CDCl_3$) δ 9.27 (s, 1H), 8.13 (d, *J* = 8.0 Hz, 1H), 7.64 (dd, *J* = 8.0, 1.4Hz, 1H), 7.34-7.28 (m, 1H), 7.08 (t, *J* = 7.6 Hz, 1H), 3.35 (s, 1H), 2.76 (s, 1H), 2.15 (s, 3H), 1.88 (s, 3H); ^{13}C NMR (100 MHz, $CDCl_3$) δ 168.5, 136.3, 131.4, 129.1, 126.4, 124.0, 123.6, 86.0, 74.5, 71.2, 30.5, 25.0; IR (cm^{-1}) v 3685, 3019, 2400, 1632, 1215.

***N*-(2-(3-Hydroxypent-1-yn-3-yl)phenyl)acetamide (2d):** 75% yield (163 mg); white solid; mp 120-122 ^{o}C; R_f = 0.25 (SiO_2, 30% EtOAc/Hexanes); ^{1}H NMR (400 MHz, $CDCl_3$) δ 9.37 (s, 1H), 8.16 (d, *J* = 8.0 Hz, 1H), 7.66 (dd, *J* = 8.0, 1.3Hz, 1H), 7.33-7.27 (m, 1H), 7.07 (t, *J* = 7.5 Hz,

1H), 3.33 (s, 1H), 2.81 (s, 1H), 2.13 (s, 3H), 2.09-2.01 (m, 2H), 0.97 (t, *J* = 7.4 Hz, 3H); ^{13}C NMR (100 MHz, $CDCl_3$) *δ* 168.5, 136.3,130.2, 128.9, 127.9, 123.6,123.2, 84.7, 76.1, 75.9, 34.9, 25.1, 9.3; IR (cm^{-1}) *v* 3371, 3020, 2401, 1677, 1215; HRMS (ESI-TOF) calcd for $C_{13}H_{14}NO$ [M + H – $H_2O]^+$ 200.1075, found 200.1067.

***N*-(2-(3-Hydroxyhex-1-yn-3-yl)phenyl)acetamide (2e):** 70% yield (162 mg); white solid; mp 148-150 °C; R_f = 0.25 (SiO_2, 30% EtOAc/Hexanes); ^{1}H NMR (400 MHz, $CDCl_3$) *δ* 9.40 (s, 1H), 8.17 (d, *J* = 8.1 Hz, 1H), 7.66 (dd, *J* = 8.1, 1.2Hz, 1H), 7.32-7.27 (m, 1H), 7.06 (t, *J* = 7.6 Hz, 1H), 3.32 (s, 1H), 2.82 (s, 1H), 2.13 (s, 3H), 2.06-1.92 (m, 2H), 1.60-1.51 (m, 1H), 1.33-1.27 (m, 1H), 0.89 (t, *J* = 7.4 Hz, 3H); ^{13}C NMR (100 MHz, $CDCl_3$) *δ* 168.6, 136.2,130.5, 128.7, 127.7, 123.5,122.9, 85.1, 75.6, 75.3, 44.1, 25.1, 18.2,14.0; IR (cm^{-1}) *v* 3373, 3020, 2401, 1676, 1216; HRMS (ESI-TOF) calcd for $C_{14}H_{17}NO$ [M + H – $H_2O]^+$ 214.1232, found 214.1223.

***N*-(2-(3-Hydroxy-4-methylpent-1-yn-3-yl)-4-methylphenyl)acetamide (2f):** 72% yield (177 mg); white solid; mp 180-182 °C; R_f = 0.25 (SiO_2, 30% EtOAc/Hexanes); ^{1}H NMR (400 MHz, $CDCl_3$) *δ* 9.24 (s, 1H), 8.02 (d, *J* = 8.2 Hz, 1H), 7.46 (s, 1H), 7.10 (dd, *J* = 8.2, 1.6Hz, 1H), 3.09 (s, 1H), 2.83 (s, 1H), 2.36-2.28 (m, 4H), 2.12 (s, 3H), 1.16 (d, *J* = 6.5 Hz, 3H), 0.76 (d, *J* = 6.5 Hz, 3H); ^{13}C NMR (100 MHz, $CDCl_3$) *δ* 167.9, 133.4, 132.8, 129.8, 129.2, 123.2, 82.9, 80.1, 77.1, 36.5, 25.0, 21.0, 18.7, 17.1; IR (cm^{-1}) *v* 3681, 3019, 2400, 1626, 1215; HRMS (ESI-TOF) calcd for $C_{15}H_{18}NO$ [M + H – $H_2O]^+$ 228.1388, found 228.1380.

***N*-(2-(1-Hydroxy-1-phenylprop-2-ynyl)phenyl)acetamide (2g)** [1]**:** 85% yield (226 mg); white solid; mp 125-127 °C; R_f = 0.30 (SiO_2, 30% EtOAc/Hexanes); ^{1}H NMR (400 MHz, $CDCl_3$) *δ* 8.39 (s, 1H), 7.97 (d, *J* = 7.9 Hz, 1H), 7.73 (d, *J* = 7.6 Hz, 1H), 7.48-7.44 (m, 2H), 7.39-7.30 (m, 4H), 7.14 (t, *J* = 7.6 Hz, 1H), 3.61 (s, 1H), 2.96 (s, 1H), 1.79 (s, 3H); ^{13}C NMR (100 MHz, $CDCl_3$) *δ* 168.3, 142.8, 136.1, 132.1, 129.4, 128.5, 128.2, 127.9, 125.6, 124.3, 123.8, 84.7, 77.4, 74.4, 24.2; IR (cm^{-1}) *v* 3683, 3021, 2401, 1679, 1215.

***N*-(2-(1-Hydroxy-1-phenylprop-2-ynyl)-5-methylphenyl)acetamide (2h) [2]:** 80% yield (224 mg); white solid; mp 155-157 °C; R_f = 0.30 (SiO_2, 30% EtOAc/Hexanes); ^{1}H NMR (400 MHz, $CDCl_3$) δ 8.16 (s, 1H), 7.78 (d, J = 8.1 Hz, 1H), 7.58 (s, 1H), 7.46 (d, J = 6.9 Hz, 2H), 7.36-7.28 (m, 3H), 7.18 (d, J = 7.2 Hz, 1H), 3.52 (s, 1H), 2.96 (s, 1H), 2.35 (s, 3H), 1.76 (s, 3H); ^{13}C NMR (100 MHz, $CDCl_3$) δ 168.2, 142.9, 133.8, 133.4, 132.4, 130.0, 128.7, 128.5, 128.4, 125.7, 124.8, 84.9, 77.5, 74.5, 24.2, 21.2; IR (cm^{-1}) v 3403, 3021, 2401, 1638, 1215; HRMS (ESI-TOF) calcd for $C_{18}H_{17}NO$ [M + H – H_2O]$^+$ 262.1232, found 262.1232.

***N*-(2-(1-(2-Chlorophenyl)-1-hydroxyprop-2-ynyl)phenyl)acetamide (2i):** 75% yield (225 mg); white solid; mp 165-167 °C; R_f = 0.30 (SiO_2, 30% EtOAc/Hexanes); ^{1}H NMR (400 MHz, $CDCl_3$) δ 8.41 (s, 1H), 7.93 (d, J = 8.0 Hz, 1H), 7.69 (d, J = 7.7 Hz, 1H), 7.62-7.57 (m, 1H), 7.42-7.35 (m, 2H), 7.32-7.27, (m, 2H), 7.14 (t, J = 7.4 Hz, 1H), 4.48 (s, 1H), 2.97 (s, 1H), 1.85 (s, 3H); ^{13}C NMR (100 MHz, $CDCl_3$) δ 168.6, 139.0, 135.9, 132.3, 131.8, 131.1, 129.9, 129.6, 129.1, 128.3, 127.1, 124.9, 124.5, 82.9, 77.5, 74.8, 24.4; IR (cm^{-1}) v 3392, 3019, 2400, 1637, 1215; HRMS (ESI-TOF) calcd for $C_{17}H_{14}ClNO$ [M + H – H_2O]$^+$ 282.0685, found 282.0676.

***N*-(4-Chloro-2-(1-hydroxy-1-phenylprop-2-ynyl)phenyl)acetamide (2j) [2]:** 80% yield (240 mg); white solid; mp 160-162 °C; R_f = 0.30 (SiO_2, 30% EtOAc/Hexanes); ^{1}H NMR (400 MHz, $CDCl_3$) δ 8.35 (s, 1H), 7.93 (d, J = 8.7 Hz, 1H), 7.74 (s, 1H), 7.47-7.42 (m, 2H), 7.38-7.29 (m, 4H), 3.79 (s, 1H), 2.98 (s, 1H), 1.78 (s, 3H); ^{13}C NMR (100 MHz, $CDCl_3$+DMSO-d_6) δ 165.8, 141.8, 133.7, 133.6, 126.8, 126.4, 126.1, 124.1, 123.4, 83.5, 76.9, 71.6, 22.8; IR (cm^{-1}) v 3414, 3021, 1648, 1515, 1216; HRMS (ESI-TOF) calcd for $C_{17}H_{14}ClNO$ [M + H – H_2O]$^+$ 282.0685, found 282.0675.

***N*-(2-(1-(4-Fluorophenyl)-1-hydroxyprop-2-ynyl)-4-methylphenyl)acetamide (2k):** 70% yield (208 mg); white solid; mp 147-149 °C; R_f = 0.30 (SiO_2, 30% EtOAc/Hexanes); ^{1}H NMR (400 MHz, $CDCl_3$) δ 8.20 (s, 1H), 7.79 (d, J = 8.2 Hz, 1H), 7.55 (s, 1H), 7.46-7.40 (m, 2H), 7.18 (dd, J

= 8.2, 1.4Hz, 1H), 7.04-6.99 (m, 2H), 3.56 (s, 1H), 2.97 (s, 1H), 2.35 (s, 3H) 1.79 (s, 3H); ^{13}C NMR (100 MHz, $CDCl_3$) δ 168.3, 163.7, 161.3, 139.0, 133.9, 133.4, 132.2, 130.0, 128.5, 127.7, 127.6, 124.8, 115.5, 115.3, 84.9, 77.5, 74.1, 24.2, 21.2; IR (cm^{-1}) *v* 3398, 3020, 2401, 1671, 1216; HRMS (ESI-TOF) calcd for $C_{18}H_{15}FNO$ $[M + H – H_2O]^+$ 280.1137, found 280.1130.

***N*-(2-(1-Hydroxy-1-(4-methoxyphenyl)prop-2-ynyl)phenyl)acetamide (2l):** 82% yield (242 mg); white solid; mp 149-151°C; R_f = 0.30 (SiO_2, 30% EtOAc/Hexanes); ^{1}H NMR (400 MHz, $CDCl_3$) δ 8.47 (s, 1H), 8.00 (d, *J* = 8.0 Hz, 1H), 7.67 (d, *J* = 7.6 Hz, 1H), 7.40-7.35 (m, 3H), 7.12 (t, *J* = 7.6 Hz, 1H), 6.87-6.84 (m, 2H), 3.79 (s, 3H), 3.57 (s, 1H), 2.94 (s, 1H), 1.85 (s, 3H); ^{13}C NMR (100 MHz, $CDCl_3$) δ 168.4, 159.6, 136.3, 134.9, 132.2, 129.4, 127.9, 127.2, 124.2, 123.9, 113.9, 85.1, 77.0, 74.3, 55.4, 24.5; IR (cm^{-1}) *v* 3397, 3021, 2401, 1637, 1216; HRMS (ESI-TOF) calcd for $C_{18}H_{17}NO_2$ $[M + H – H_2O]^+$ 278.1181, found 278.1173.

1-(2-Acetamidophenyl)prop-2-ynyl acetate (3): 80% yield (185 mg); white solid; mp 118-120 °C; R_f = 0.60 (SiO_2, 30% EtOAc/Hexanes); ^{1}H NMR (400 MHz, $CDCl_3$) δ 8.41 (s, 1H), 7.89 (d, *J* = 8.0 Hz, 1H), 7.63 (dd, *J* = 7.8, 1.3 Hz, 1H), 7.41-7.36 (m, 1H), 7.18 (t, *J* = 7.8 Hz, 1H), 6.52 (d, *J* = 2.2 Hz, 1H), 2.71 (d, *J* = 2.2 Hz, 1H), 2.22 (s, 3H), 2.11 (s, 3H); ^{13}C NMR (100 MHz, $CDCl_3$) δ 170.7, 168.7, 135.6, 130.3, 129.6, 127.3, 125.3, 124.7, 79.3, 76.3, 62.9, 24.3, 20.9; IR (cm^{-1}) *v* 3355, 3020, 2402, 1688, 1217;.HRMS (ESI-TOF) calcd for $C_{13}H_{14}NO_3$ $[M + Na]^+$ 254.0793, found 254.0780.

(*E*)-3-(2-Acetamidophenyl)-*N*-tosylacrylamide (5): 46% yield (165 mg); white solid; mp 162-164 °C; R_f = 0.25 (SiO_2, 30% EtOAc/Hexanes); ^{1}H NMR (400 MHz, DMSO-d_6) δ 12.25 (s, 1H), 9.76 (s, 1H), 7.87 (d, *J* = 8.2 Hz, 2H), 7.67 (d, *J* = 15.7 Hz, 1H), 7.58 (d, *J* = 7.6 Hz, 1H), 7.46-7.37 (m, 4H), 7.23 (d, *J* = 7.6 Hz, 1H), 6.54 (d, *J* = 15.7 Hz, 1H), 2.41 (s, 3H), 2.06 (s, 3H); ^{13}C NMR (100 MHz, DMSO-d_6) δ 169.2, 163.8, 144.7, 140.2, 137.7, 137.0, 131.1, 130.0, 128.6,

128.2, 126.9, 126.9, 126.1, 120.1, 23.7, 21.5; IR (cm^{-1}) *v* 3385, 3021, 1626, 1415, 1216; HRMS (ESI-TOF) calcd for $C_{18}H_{19}N_2O_4S$ $[M + H]^+$ 359.1066, found 359.1057.

(*E*)-4-Methyl-*N*-(quinolin-2(1*H*)-ylidene)benzenesulfonamide (9a): 62% yield (185 mg); white solid; mp 145-147 ^oC; R_f = 0.20 (SiO_2, 30% EtOAc/Hexanes); ^1H NMR (400 MHz, $CDCl_3$) δ 11.92 (s, 1H), 7.91 (d, *J* = 8.3 Hz, 2H), 7.87 (d, *J* = 9.4 Hz, 1H), 7.65-7.60 (m, 2H), 7.49 (d, *J* = 8.6 Hz, 1H), 7.39-7.34 (m, 1H), 7.27 (d, *J* = 8.3 Hz, 2H), 7.00 (d, *J* = 9.4 Hz, 1H), 2.40 (s, 3H); ^{13}C NMR (100 MHz, $CDCl_3$) δ 154.6, 142.6, 140.9, 140.0, 136.7, 131.8, 129.4, 128.1, 126.3, 124.7, 121.4, 120.1, 117.6, 21.5; IR (cm^{-1}) *v* 3411, 3020, 1636, 1415, 1216; HRMS (ESI-TOF) calcd for $C_{16}H_{15}N_2O_2S$ $[M + H]^+$ 299.0854, found 299.0845.

(*E*)-*N*-(Quinolin-2(1*H*)-ylidene)-4-(trifluoromethyl)benzenesulfonamide (9b): 71% yield (250 mg); white solid; mp 180-182 ^oC; R_f = 0.20 (SiO_2, 30% EtOAc/Hexanes); ^1H NMR (400 MHz, DMSO-d_6) δ 13.43 (s, 1H), 8.30 (d, *J* = 9.5 Hz, 1H), 8.10 (d, *J* = 7.5 Hz, 2H), 7.92 (d, *J* = 8.3 Hz, 2H), 7.85 (d, *J* = 7.5 Hz, 1H), 7.74- 7.68 (m, 1H), 7.60 (d, *J* = 8.3 Hz, 2H), 7.44-7.38 (m, 1H); ^{13}C NMR (100 MHz, DMSO-d_6) δ 156.0, 148.0, 142.8, 137.8, 132.7, 132.1, 131.8, 128.7, 128.1, 127.3, 126.6, 126.5, 126.5, 125.4, 124.8, 122.7. 121.4, 119.9, 117.4, 115.9; IR (cm^{-1}) *v* 3389, 3021, 1636, 1408, 1215;. HRMS (ESI-TOF) calcd for $C_{16}H_{12}F_3N_2O_2S$ $[M + H]^+$ 353.0572, found 353.0542.

(*E*)-*N*-(Quinolin-2(1*H*)-ylidene)-2-(trifluoromethyl)benzenesulfonamide (9c): 55% yield (194 mg); white solid; mp 178-180 ^oC; R_f = 0.20 (SiO_2, 30% EtOAc/Hexanes); ^1H NMR (400 MHz, DMSO-d_6) δ 13.33 (s, 1H), 8.33-8.26 (m, 2H), 7.93 (d, *J* = 8.2 Hz, 1H), 7.88-7.83 (m, 2H), 7.78 (t, *J* = 7.8 Hz, 1H), 7.71 (t, *J* = 7.8 Hz, 1H), 7.62 (d, *J* = 8.2 Hz, 1H), 7.49 (d, *J* = 9.5 Hz, 1H) 7.44-7.38 (m, 1H); ^{13}C NMR (100 MHz, DMSO-d_6) δ 155.8, 142.6, 142.5, 137.8, 133.4, 132.6, 130.4, 128.7, 128.6, 128.5, 128.4, 128.4, 126.4, 126.1, 124.9, 124.8, 122.2, 121.3, 117.4, 116.4;

IR (cm^{-1}) *v* 3419, 3021, 1637, 1417, 1215; HRMS (ESI-TOF) calcd for $C_{16}H_{12}F_3N_2O_2S$ $[M + H]^+$ 353.0572, found 353.0542.

(*E*)-*N*-(Quinolin-2(1*H*)-ylidene)benzenesulfonamide (9d): 46% yield (131 mg); white solid; mp 170-172 °C; R_f = 0.20 (SiO_2, 30% EtOAc/Hexanes); ^1H NMR (400 MHz, $CDCl_3$) δ 11.91 (s, 1H), 8.03-7.99 (m, 2H), 7.87 (d, *J* = 9.4 Hz, 1H), 7.64-7.59 (m, 2H), 7.53-7.43 (m, 4H), 7.38-7.34 (m, 1H), 6.96 (d, *J* = 9.4 Hz, 1H); ^{13}C NMR (100 MHz, $CDCl_3$) δ 154.7, 142.9, 141.1, 136.6, 132.0, 131.9, 128.8, 128.1, 126.3, 124.8, 121.4, 120.3, 117.5; IR (cm^{-1}) *v* 3411, 3021, 1636, 1393, 1215; HRMS (ESI-TOF) calcd for $C_{15}H_{13}N_2O_2S$ $[M + H]^+$ 285.0698, found 285.0689.

(*E*)-*N*-(Quinolin-2(1*H*)-ylidene)methanesulfonamide (9e): 36% yield (80mg); white solid; mp 200-202 °C; R_f = 0.15 (SiO_2, 30% EtOAc/Hexanes); ^1H NMR (400 MHz, DMSO-d_6) δ 12.89 (s, 1H), 8.23 (d, *J* = 9.3 Hz, 1H), 7.84 (d, *J* = 7.9 Hz, 1H), 7.71-7.62 (m, 2H), 7.45-7.35 (m, 2H), 3.32 (s, 3H); ^{13}C NMR (100 MHz, DMSO-d_6) δ 154.9, 140.9, 131.8, 128.5, 124.4, 122.0, 119.5, 118.2, 115.9, 42.5; IR (cm^{-1}) *v* 3397, 3021, 1637, 1392, 1215; HRMS (ESI-TOF) calcd for C10H11N2O2S [M + H]+ 223.0541, found 223.0533.

(*E*)-*N*-(7-Fluoroquinolin-2(1*H*)-ylidene)-4-methylbenzenesulfonamide (9f): 49% yield (155 mg); white solid; mp 190-192 °C; R_f = 0.15 (SiO_2, 30% EtOAc/Hexanes); ^1H NMR (400 MHz, DMSO-d_6) δ 13.21 (s, 1H), 8.23 (d, *J* = 9.4 Hz, 1H), 7.91 (dd, *J* = 6.2, 2.5Hz, 1H), 7.86-7.76 (m, 2H), 7.44 (s, 1H), 7.37-7.24 (m, 4H), 2.34 (s, 3H); ^{13}C NMR (100 MHz, DMSO-d_6) δ 163.2, 162.7, 155.6, 142.5, 141.2, 131.5, 131.4, 129.7, 126.8, 118.6, 115.1, 113.6, 103.2, 21.4; IR (cm^{-1}) *v* 3411, 3021, 1637, 1412, 1216.HRMS (ESI-TOF) calcd for $C_{16}H_{14}FN_2O_2S$ $[M + H]^+$ 317.0760, found 317.0759.

(*E*)-*N*-(7-Fluoroquinolin-2(1*H*)-ylidene)-4-(trifluoromethyl)benzenesulfonamide (9g): 50% yield (185 mg); white solid; mp 196-198 °C; R_f = 0.15 (SiO_2, 30% EtOAc/Hexanes); ^1H NMR (400 MHz, DMSO-d_6) δ 13.43 (s, 1H), 8.30 (d, *J* = 9.5 Hz, 1H), 8.12 (d, *J* = 6.5 Hz, 2H), 7.99-

7.91 (m, 3H), 7.52, (s, 1H), 7.36-7.27 (m, 2H); ^{13}C NMR (100 MHz, DMSO-d_6) δ 165.3, 162.8, 156.0, 147.5, 142.1, 132.2, 131.9, 131.7, 131.6, 128.1, 127.5, 126.6, 126.6, 126.5, 125.4, 122.6, 119.9, 114.9, 113.9, 113.7; IR (cm^{-1}) v 3414, 3022, 1637, 1404, 1216;.HRMS (ESI-TOF) calcd for $C_{16}H_{11}F_4N_2O_2S$ [M + H]$^+$ 371.0477, found 371.0445.

(*E*)-*N*-(7-Fluoroquinolin-2(1*H*)-ylidene)-2-(trifluoromethyl)benzenesulfonamide (9h): 48% yield (178mg); white solid; mp 130-132 ^{o}C; R_f = 0.15 (SiO_2, 30% EtOAc/Hexanes); ^{1}H NMR (400 MHz, DMSO-d_6) δ 13.34 (s, 1H), 8.35-8.26 (m, 2H), 7.98-7.92 (m, 2H), 7.87 (t, J = 7.3 Hz, 1H), 7.79 (t, J = 7.3 Hz, 1H), 7.45 (d, J = 8.8 Hz, 1H), 7.36-7.30 (m, 2H); ^{13}C NMR (100 MHz, DMSO-d_6) δ 165.4, 162.9, 156.1, 142.4, 142.2, 139.3, 133.5, 132.7, 131.6, 130.4, 128.5, 128.4, 126.4, 126.1, 125.8, 124.9, 122.1, 118.4, 115.7, 113.7, 113.5, 103.2, 102.9; IR (cm^{-1}) v 3400, 3021, 1637, 1417, 1215;. HRMS (ESI-TOF) calcd for $C_{16}H_{11}F_4N_2O_2S$ [M + H]$^+$ 371.0477, found 371.0445.

(*E*)-*N*-(7-Fluoroquinolin-2(1*H*)-ylidene)benzenesulfonamide (9i): 45% yield (136 mg); white solid; mp 180-182 ^{o}C; R_f = 0.15 (SiO_2, 30% EtOAc/Hexanes); ^{1}H NMR (400 MHz, DMSO-d_6) δ 13.27 (s, 1H), 8.24 (d, J = 9.4 Hz, 1H), 7.94-7.89 (m, 3H), 7.59-7.52 (m, 4H), 7.28 (t, J = 8.5 Hz, 2H); ^{13}C NMR (100 MHz, DMSO-d_6) δ 165.6, 163.1, 154.6, 142.5, 140.4, 138.5, 138.4, 132.2, 130.3, 130.2, 128.8, 126.3, 119.2, 118.3, 113.9, 113.7, 104.3, 104.0; IR (cm^{-1}) v 3396, 3021, 1637, 1416, 1216. HRMS (ESI-TOF) calcd for $C_{15}H_{12}FN_2O_2S$ [M + H]$^+$ 303.0604, found 303.0572.

(*E*)-4-Methyl-*N*-(4-methylquinolin-2(1*H*)-ylidene)benzenesulfonamide (9j): 77% yield (241 mg); white solid; mp 128-130 ^{o}C; R_f = 0.25 (SiO_2, 30% EtOAc/Hexanes); ^{1}H NMR (400 MHz, $CDCl_3$) δ 11.79 (s, 1H), 7.87 (d, J = 8.2 Hz, 2H), 7.74 (d, J = 8.0 Hz, 1H), 7.63-7.57 (m, 1H), 7.59 (d, J = 8.0Hz, 1H), 7.39-7.34 (m, 1H), 7.24 (d, J = 8.2 Hz, 2H), 6.82 (s, 1H), 2.52 (s, 3H), 2.37 (s, 3H); ^{13}C NMR (100 MHz, $CDCl_3$) δ 154.2, 150.1, 142.5, 140.3, 136.2, 131.6, 129.3,

126.2, 124.6, 121.9, 119.6, 117.8, 21.7, 19.3; IR (cm^{-1}) v 3423, 3043, 1633, 1515, 1218; HRMS (ESI-TOF) calcd for $C_{17}H_{17}N_2O_2S$ $[M + H]^+$ 313.1011, found 313.1005.

(*E*)-N-(4-Methylquinolin-2(1*H*)-ylidene)benzenesulfonamide (9k): 58% yield (173 mg); white solid; mp 210-212 °C; R_f = 0.25 (SiO_2, 30% EtOAc/Hexanes); ^{1}H NMR (400 MHz, DMSO-d_6) δ 13.05 (s, 1H), 7.93-7.86 (m, 3H), 7.70-7.65 (m, 1H), 7.58-7.51 (m, 4H), 7.44 (s, 1H), 7.42-7.37 (m, 1H), 2.58 (s, 3H); ^{13}C NMR (100 MHz, DMSO-d_6) δ 155.2, 151.2, 144.3, 137.6, 132.3, 132.0, 129.3, 126.4, 125.5, 124.5, 121.5, 117.6, 115.4, 19.7; IR (cm^{-1}) v 3391, 3021, 1636, 1408, 1215 HRMS (ESI-TOF) calcd for $C_{16}H_{15}N_2O_2S$ $[M + H]^+$ 299.0854, found 299.0846.

(*E*)-*N*-(4-methylquinolin-2(1*H*)-ylidene)methanesulfonamide (9l): 48% yield (114mg); white solid; mp 115-117 °C; R_f = 0.25 (SiO_2, 30% EtOAc/Hexanes); ^{1}H NMR (400 MHz, $CDCl_3$) δ 11.51 (s, 1H), 7.75 (d, J = 7.9 Hz, 1H), 7.59 (t, J = 7.4 Hz, 1H), 7.41-7.34 (m, 2H), 6.74 (s, 1H), 3.01 (s, 3H), 2.55 (s, 3H); ^{13}C NMR (100 MHz, $CDCl_3$) δ 153.9, 149.7, 136.0, 131.4, 124.6, 124.5, 121.8, 120.2, 117.5, 42.7, 19.2; IR (cm^{-1}) v 3404, 3021, 1638, 1410, 1215;.HRMS (ESI-TOF) calcd for $C_{11}H_{13}N_2O_2S$ [M + H]+ 237.0698, found 237.0673.

(*E*)-*N*-(4-Ethylquinolin-2(1*H*)-ylidene)-4-methylbenzenesulfonamide (9m): 58% yield (189 mg); white solid; mp 158-160 °C; R_f = 0.25 (SiO_2, 30% EtOAc/Hexanes); ^{1}H NMR (400 MHz, $CDCl_3$) δ 11.82 (s, 1H), 7.88 (d, J = 8.0 Hz, 2H), 7.78 (d, J = 8.1 Hz, 1H), 7.59 (t, J = 7.5Hz, 1H), 7.43 (d, J = 8.1 Hz, 1H), 7.36 (t, J = 7.5 Hz, 1H), 7.25 (d, J = 8.0 Hz, 2H), 6.79 (s, 1H), 2.94-2.90 (m, 2H) 2.38 (s, 3H), 1.32 (t, J = 7.3 Hz, 3H); ^{13}C NMR (100 MHz, $CDCl_3$) δ 155.3, 154.4, 142.5, 140.3, 136.4, 131.4, 129.3, 126.3, 124.5, 124.1, 121.2, 118.0, 117.7, 25.3, 21.5, 12.8; IR (cm^{-1}) v 3423, 3020, 1633, 1399, 1216; HRMS (ESI-TOF) calcd for $C_{18}H_{19}N_2O_2S$ $[M + H]^+$ 327.1167, found 327.1157.

(*E*)-4-Methyl-*N*-(4-propylquinolin-2(1*H*)-ylidene)benzenesulfonamide (9n): 61% yield (208 mg); white solid; mp 130-132 °C; R_f = 0.25 (SiO_2, 30% EtOAc/Hexanes); ^{1}H NMR (400 MHz,

$CDCl_3$) δ 11.82 (s, 1H), 7.88 (d, *J* = 7.8 Hz, 2H), 7.78 (d, *J* = 8.0 Hz, 1H), 7.59 (t, *J* = 7.3Hz, 1H), 7.43 (d, *J* = 8.0 Hz, 1H), 7.36 (t, *J* = 7.3 Hz, 1H), 7.25 (d, *J* = 7.8 Hz, 2H), 6.78 (s, 1H), 2.84 (t, *J* = 7.0 Hz, 2H), 2.38 (s, 3H), 1.78-1.67 (m, 2H), 1.02 (t, *J* = 7.2 Hz, 3H); ^{13}C NMR (100 MHz, $CDCl_3$) δ 154.2, 153.9, 142.5, 140.3, 136.5, 131.4, 129.4, 126.3, 124.5, 124.3, 121.3, 118.8, 118.0, 34.4, 22.1, 21.5, 13.9; IR (cm^{-1}) *v* 3400, 3019, 1634, 1413, 1215; HRMS (ESI-TOF) calcd for $C_{19}H_{21}N_2O_2S$ [M + H]$^+$ 341.1324, found 341.1319.

(*E*)-*N*-(4-Isopropyl-6-methylquinolin-2(1*H*)-ylidene)-4-methylbenzenesulfonamide (9o): 67% yield (237 mg); white solid; mp 208-210 °C; R_f = 0.25 (SiO_2, 30% EtOAc/Hexanes); ^{1}H NMR (400 MHz, $CDCl_3$) δ 11.79 (s, 1H), 7.89 (d, *J* = 8.0 Hz, 2H), 7.61 (s, 1H), 7.42 (d, *J* = 8.2 Hz, 1H), 7.33 (d, *J* = 8.2Hz, 1H), 7.25 (d, *J* = 8.0 Hz, 2H), 6.77 (s, 1H), 3.49-3.39 (m, 1H), 2.47 (s, 3H), 2.38 (s, 3H), 1.31 (d, *J* = 6.6 Hz, 6H); ^{13}C NMR (100 MHz, $CDCl_3$) δ 159.3, 153.9, 142.5, 140.4, 134.5, 134.3, 132.6, 129.3, 126.3, 123.5, 120.7, 117.9, 116.5, 28.8, 22.3, 21.5, 21.4; IR (cm^{-1}) *v* 3428, 3021, 1635, 1521, 1215; HRMS (ESI-TOF) calcd for $C_{20}H_{23}N_2O_2S$ [M + H]$^+$ 355.1480, found 355.1470.

(*E*)-4-Methyl-*N*-(4-phenylquinolin-2(1*H*)-ylidene)benzenesulfonamide (9p): 61% yield (228 mg); white solid; mp 155-157 °C; R_f = 0.30 (SiO_2, 30% EtOAc/Hexanes); ^{1}H NMR (400 MHz, $CDCl_3$) δ 11.89 (s, 1H), 7.91 (d, *J* = 8.0 Hz, 2H), 7.64-7.60 (m, 2H), 7.55-7.46 (m, 4H), 7.43-7.41 (m, 2H), 7.31-7.27 (m, 3H), 6.86 (s, 1H), 2.39 (s, 3H); ^{13}C NMR (100 MHz, $CDCl_3$) δ 153.7, 153.4, 142.7, 140.1, 137.0, 136.2, 131.6,129.4, 129.3, 128.8, 128.8, 127.0, 126.3, 124.6, 121.0, 120.4, 117.8, 21.5; IR (cm^{-1}) *v* 3423, 3021, 1631, 1524, 1215; HRMS (ESI-TOF) calcd for $C_{22}H_{19}N_2O_2S$ [M + H]$^+$ 375.1167, found 375.1162.

(*E*)-4-Methyl-*N*-(7-methyl-4-phenylquinolin-2(1*H*)-ylidene)benzenesulfonamide (9q): 72% yield (280 mg); white solid; mp 215-217 °C; R_f = 0.30 (SiO_2, 30% EtOAc/Hexanes); ^{1}H NMR (400 MHz, $CDCl_3$) δ 11.85 (s, 1H), 7.90 (d, *J* = 8.0 Hz, 2H), 7.59-7.47 (m, 3H), 7.46-7.34 (m,

5H), 7.28-7.24 (m, 2H), 6.84 (s, 1H), 2.38(s, 3H), 2.37 (s, 3H); ^{13}C NMR (100 MHz, $CDCl_3$) δ 153.5, 153.2, 142.6, 140.3, 136.4, 135.2, 134.5, 133.2, 129.4, 129.3, 128.8, 128.7, 126.3, 121.0, 120.2, 117.7, 21.5, 21.2; IR (cm^{-1}) *v* 3426, 3021, 1637, 1416, 1284; HRMS (ESI-TOF) calcd for $C_{23}H_{21}N_2O_2S$ [M + H]$^+$ 389.1324, found 389.1319.

(*E*)-*N*-(4-(2-Chlorophenyl)quinolin-2(1*H*)-ylidene)-4-methylbenzenesulfonamide (9r): 69% yield (282 mg); white solid; mp 135-137 °C; R_f = 0.30 (SiO_2, 30% EtOAc/Hexanes); ^{1}H NMR (400 MHz, $CDCl_3$) δ 11.85 (s, 1H), 7.92 (d, *J* = 7.9 Hz, 2H), 7.61 (t, *J* = 7.2 Hz, 1H), 7.56-7.36 (m, 5H), 7.33-7.19 (m, 4H), 6.84 (s, 1H), 2.40 (s, 3H); ^{13}C NMR (100 MHz, $CDCl_3$) δ 153.7,150.9, 142.8, 139.9, 136.8, 135.0, 132.7, 131.7, 130.6, 130.5, 130.0, 129.5, 129.4, 127.1, 126.8, 126.4, 124.7, 121.2, 117.8, 21.5; IR (cm^{-1}) *v* 3429, 3021, 1631, 1526, 1215; HRMS (ESI-TOF) calcd for $C_{22}H_{18}ClN_2O_2S$ [M + H]+ 409.0778, found 409.0767.

(*E*)-*N*-(6-Chloro-4-phenylquinolin-2(1*H*)-ylidene)-4-methylbenzenesulfonamide (9s): 55% yield (224 mg); white solid; mp 165-167 °C; R_f = 0.30 (SiO_2, 30% EtOAc/Hexanes); ^{1}H NMR (400 MHz, $CDCl_3$) δ 11.63 (s, 1H), 7.89 (d, *J* = 8.0 Hz, 2H), 7.64-7.50 (m, 5H), 7.45 (d, *J* = 8.5 Hz, 1H), 7.42-7.35 (m, 2H), 7.28 (d, *J* = 8.0 Hz, 2H), 6.91 (s, 1H), 2.39 (s, 3H); ^{13}C NMR (100 MHz, $CDCl_3$) δ 153.2, 152.2, 143.0, 139.6, 136.2, 135.7, 131.8, 130.3, 129.6, 129.5,129.0, 128.7, 126.4, 126.1, 122.2, 120.2, 119.8, 21.5; IR (cm^{-1}) *v* 3427, 3020, 1663, 1427, 1286; HRMS (ESI-TOF) calcd for $C_{22}H_{18}ClN_2O_2S$ [M + H]$^+$ 409.0778, found 409.0769.

(*E*)-*N*-(4-(4-Fluorophenyl)-6-methylquinolin-2(1*H*)-ylidene)-4-methylbenzenesulfonamide (9t): 60% yield (244 mg); white solid; mp 205-207 °C; R_f = 0.30 (SiO_2, 30% EtOAc/Hexanes); ^{1}H NMR (400 MHz, $CDCl_3$) δ 11.86 (s, 1H), 7.92 (d, *J* = 8.2 Hz, 2H), 7.50-7.39 (m, 4H), 7.36 (s, 1H), 7.28 (d, *J* = 8.2 Hz, 2H), 7.23 (t, *J* = 8.6 Hz, 2H), 6.85 (s, 1H), 2.41 (s, 3H), 2.40 (s, 3H); ^{13}C NMR (100 MHz, $CDCl_3$) δ 164.6, 162.2, 153.4, 152.1, 142.8, 140.3, 135.3, 134.8, 133.4, 132.4, 130.8, 130.7, 129.5, 126.4, 126.2, 121.1, 120.7, 117.8, 116.2, 115.9, 21.6, 21.4; IR (cm^{-1}) *v*

3423, 3021, 1630, 1409, 1216.HRMS (ESI-TOF) calcd for $C_{23}H_{20}FN_2O_2S$ [M + H]$^+$ 407.1230, found 407.1217.

(*E*)-*N*-(4-(4-Methoxyphenyl)quinolin-2(1*H*)-ylidene)-4-methylbenzenesulfonamide (9u): 65% yield (263 mg); white solid; mp 130-132 °C; R_f = 0.30 (SiO_2, 30% EtOAc/Hexanes); ^{1}H NMR (400 MHz, $CDCl_3$) δ 11.89 (s, 1H), 7.91 (d, *J* = 7.6 Hz, 2H), 7.70 (d, *J* = 7.3 Hz, 1H), 7.62 (t, *J* = 6.8 Hz, 1H), 7.49 (d, *J* = 7.3 Hz, 1H), 7.41-7.23 (m, 5H), 7.03 (d, *J* = 7.6 Hz, 2H), 6.85 (s, 1H), 3.88 (s, 3H), 2.39 (s, 3H); ^{13}C NMR (100 MHz, $CDCl_3$) δ 160.6, 153.8, 153.2, 142.6, 140.2, 137.0, 131.5, 130.3, 129.4, 128.5, 127.0, 126.3, 124.5, 121.1, 120.1, 117.7, 114.3, 55.4, 21.5; IR (cm^{-1}) v 3431, 3021, 1627, 1522, 1215; HRMS (ESI-TOF) calcd for $C_{23}H_{21}N_2O_3S$ [M + H]$^+$ 405.1273, found 405.1261.

References

1, Kothandaraman, P.; Koh, B. Q.; Limpanuparb, T.; Hirao, H.; Chan, P. W. H. *Chem. Eur. J*. **2013,** *19*, 1978–1985.

2. Kothandaraman, P.; Lauw, S. J. L.; Chan, P. W. H. *Tetrahedron* **2013,** *69*, 7471–7480.

Copies of ^{1}H and ^{13}C NMR Spectra

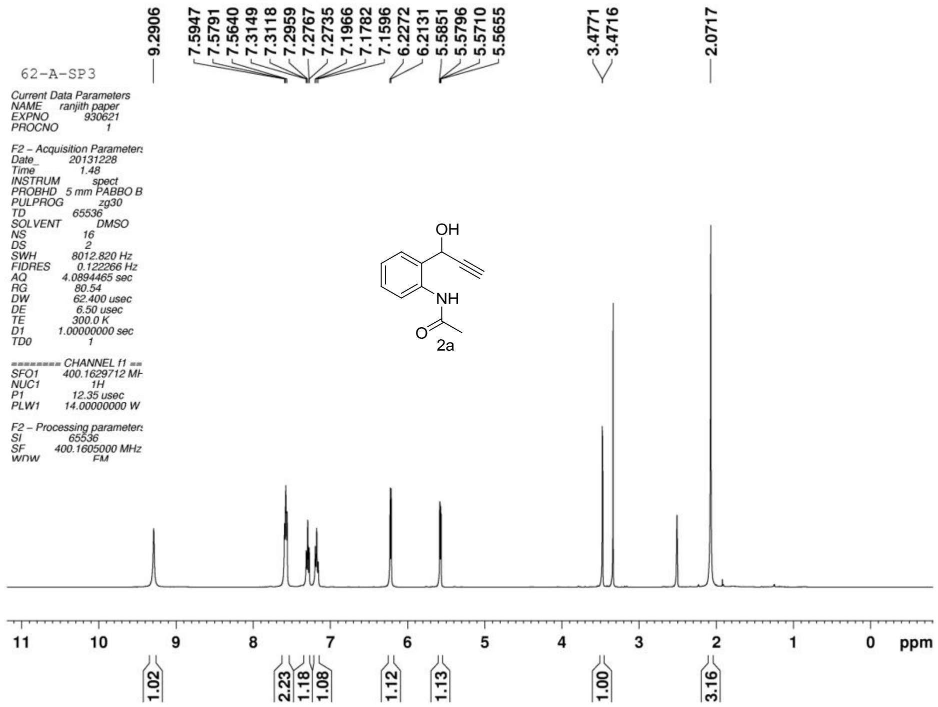

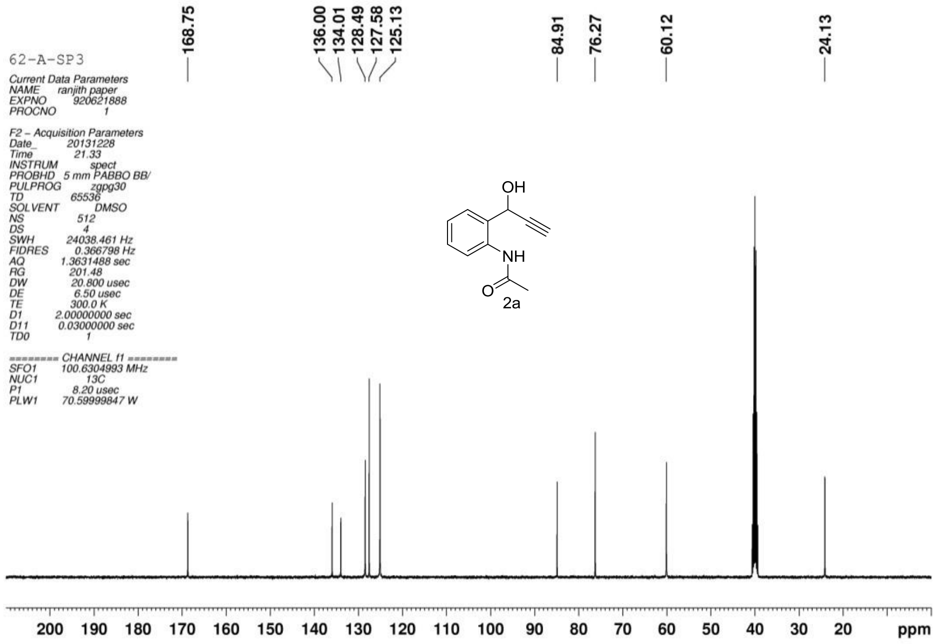

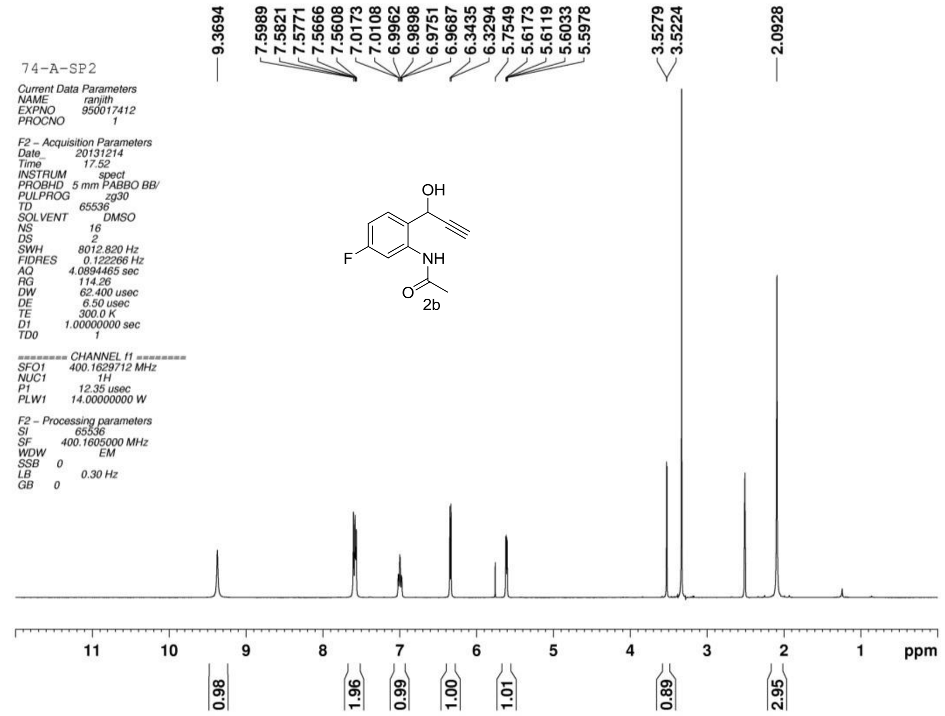
74-A-SP2
Current Data Parameters
NAME ranjith
EXPNO 950017412
PROCNO 1
F2 - Acquisition Parameters
Date_ 20131214
Time 17.52
INSTRUM spect
PROBHD 5 mm PABBO BB/
PULPROG zg30
TD 65536
SOLVENT DMSO
NS 16
DS 2
SWH 8012.820 Hz
FIDRES 0.122266 Hz
AQ 4.0894465 sec
RG 114.26
DW 62.400 usec
DE 6.50 usec
TE 300.0 K
D1 1.00000000 sec
TD0 1
======== CHANNEL f1 ========
SFO1 400.1629712 MHz
NUC1 1H
P1 12.35 usec
PLW1 14.00000000 W
F2 - Processing parameters
SI 65536
SF 400.1605000 MHz
WDW EM
SSB 0
LB 0.30 Hz
GB 0
OH
F
NH
O
2b
9.3694
7.5989
7.5821
7.5771
7.5666
7.5608
7.0173
7.0108
6.9962
6.9898
6.9751
6.9687
6.3435
6.3294
5.7549
5.6173
5.6119
5.6033
5.5978
3.5279
3.5224
2.0928
11 10 9 8 7 6 5 4 3 2 1 ppm
0.98
1.96
0.99
1.00
1.01
0.89
2.95

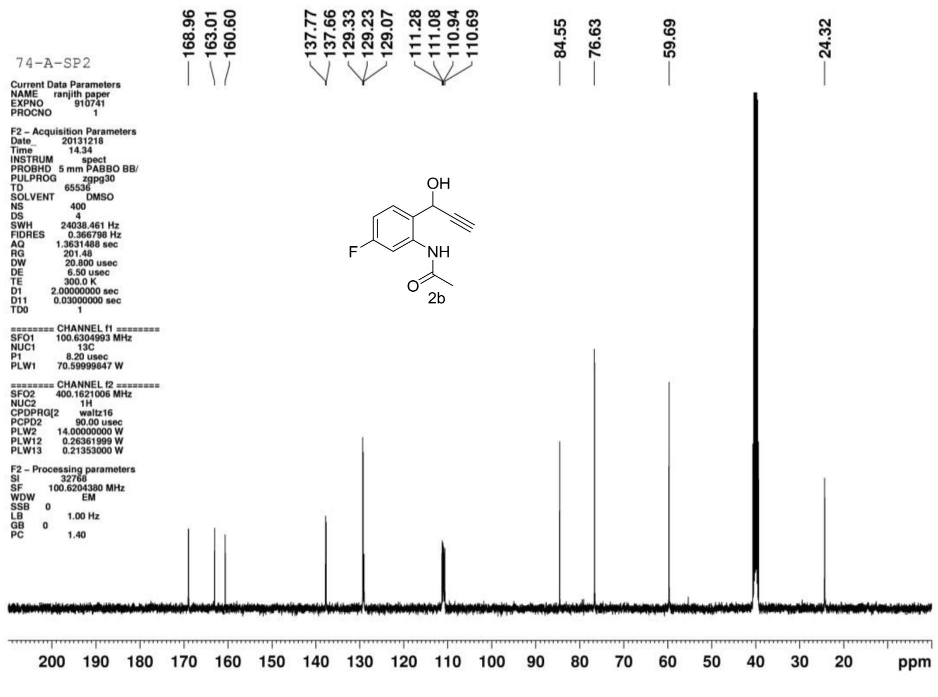
74-A-SP2
Current Data Parameters
NAME ranjith paper
EXPNO 910741
PROCNO 1
F2 - Acquisition Parameters
Date_ 20131218
Time 14.34
INSTRUM spect
PROBHD 5 mm PABBO BB/
PULPROG zgpg30
TD 65536
SOLVENT DMSO
NS 400
DS 4
SWH 24038.461 Hz
FIDRES 0.366798 Hz
AQ 1.3631488 sec
RG 201.48
DW 20.800 usec
DE 6.50 usec
TE 300.0 K
D1 2.00000000 sec
D11 0.03000000 sec
TD0 1
======== CHANNEL f1 ========
SFO1 100.6304993 MHz
NUC1 13C
P1 8.20 usec
PLW1 70.59999847 W
======== CHANNEL f2 ========
SFO2 400.1621006 MHz
NUC2 1H
CPDPRG[2 waltz16
PCPD2 90.00 usec
PLW2 14.00000000 W
PLW12 0.26361999 W
PLW13 0.21353000 W
F2 - Processing parameters
SI 32768
SF 100.6204380 MHz
WDW EM
SSB 0
LB 1.00 Hz
GB 0
PC 1.40
OH
F
NH
O
2b
168.96
163.01
160.60
137.77
137.66
129.33
129.23
129.07
111.28
111.08
110.94
110.69
84.55
76.63
59.69
24.32
200 190 180 170 160 150 140 130 120 110 100 90 80 70 60 50 40 30 20 ppm

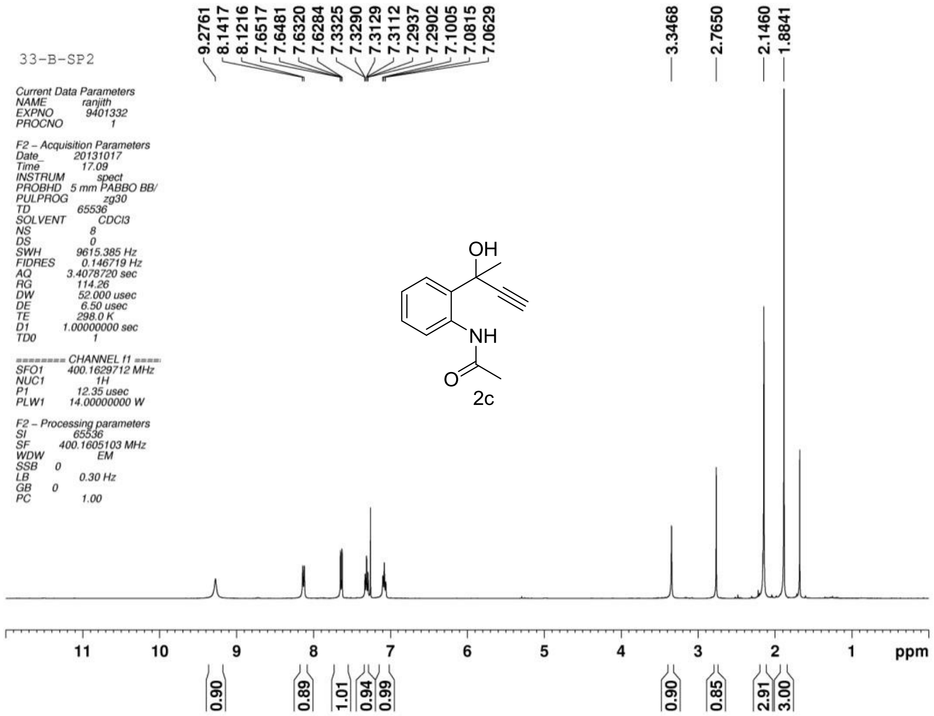
33-B-SP2
Current Data Parameters
NAME ranjith
EXPNO 9401332
PROCNO 1
F2 – Acquisition Parameters
Date_ 20131017
Time 17.09
INSTRUM spect
PROBHD 5 mm PABBO BB/
PULPROG zg30
TD 65536
SOLVENT CDCl3
NS 8
DS 0
SWH 9615.385 Hz
FIDRES 0.146719 Hz
AQ 3.4078720 sec
RG 114.26
DW 52.000 usec
DE 6.50 usec
TE 298.0 K
D1 1.00000000 sec
TD0 1
======= CHANNEL f1 =====
SFO1 400.1629712 MHz
NUC1 1H
P1 12.35 usec
PLW1 14.00000000 W
F2 – Processing parameters
SI 65536
SF 400.1605103 MHz
WDW EM
SSB 0
LB 0.30 Hz
GB 0
PC 1.00
9.2761
8.1417
8.1216
7.6517
7.6481
7.6320
7.6284
7.3325
7.3290
7.3129
7.3112
7.2937
7.2902
7.1005
7.0815
7.0629
3.3468
2.7650
2.1460
1.8841
OH
NH
O
2c
11
10
9
8
7
6
5
4
3
2
1
ppm
0.90
0.89
1.01
0.94
0.99
0.90
0.85
2.91
3.00

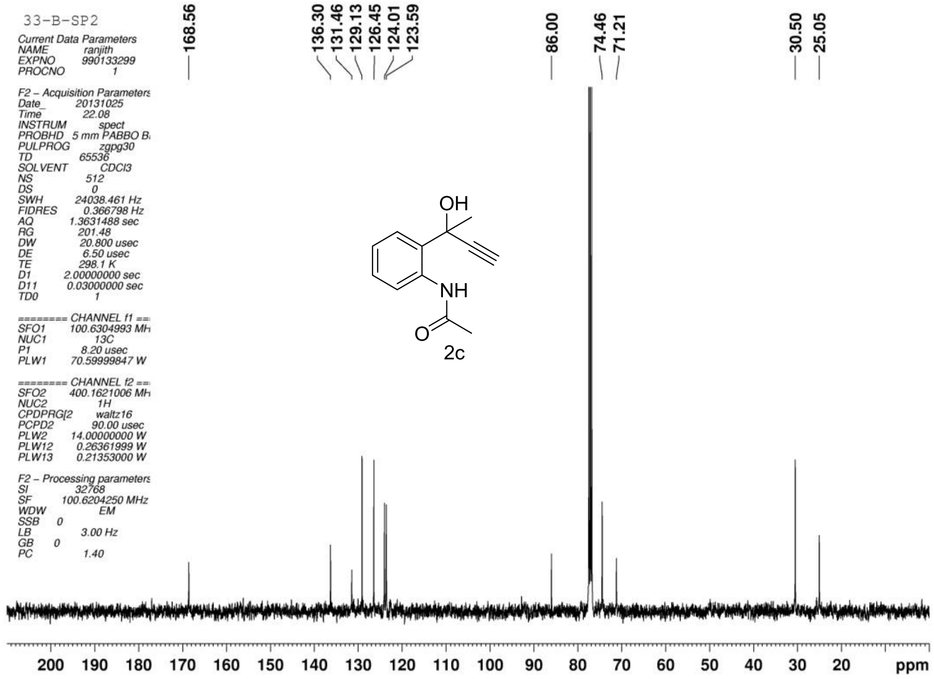
33-B-SP2
Current Data Parameters
NAME ranjith
EXPNO 990133299
PROCNO 1
F2 – Acquisition Parameters
Date_ 20131025
Time 22.08
INSTRUM spect
PROBHD 5 mm PABBO B
PULPROG zgpg30
TD 65536
SOLVENT CDCl3
NS 512
DS 0
SWH 24038.461 Hz
FIDRES 0.366798 Hz
AQ 1.3631488 sec
RG 201.48
DW 20.800 usec
DE 6.50 usec
TE 298.1 K
D1 2.00000000 sec
D11 0.03000000 sec
TD0 1
======= CHANNEL f1 ===
SFO1 100.6304993 MH
NUC1 13C
P1 8.20 usec
PLW1 70.59999847 W
======= CHANNEL f2 ===
SFO2 400.1621006 MH
NUC2 1H
CPDPRG[2 waltz16
PCPD2 90.00 usec
PLW2 14.00000000 W
PLW12 0.26361999 W
PLW13 0.21353000 W
F2 – Processing parameters
SI 32768
SF 100.6204250 MHz
WDW EM
SSB 0
LB 3.00 Hz
GB 0
PC 1.40
168.56
136.30
131.46
129.13
126.45
124.01
123.59
86.00
74.46
71.21
30.50
25.05
OH
NH
O
2c
200
190
180
170
160
150
140
130
120
110
100
90
80
70
60
50
40
30
20
ppm

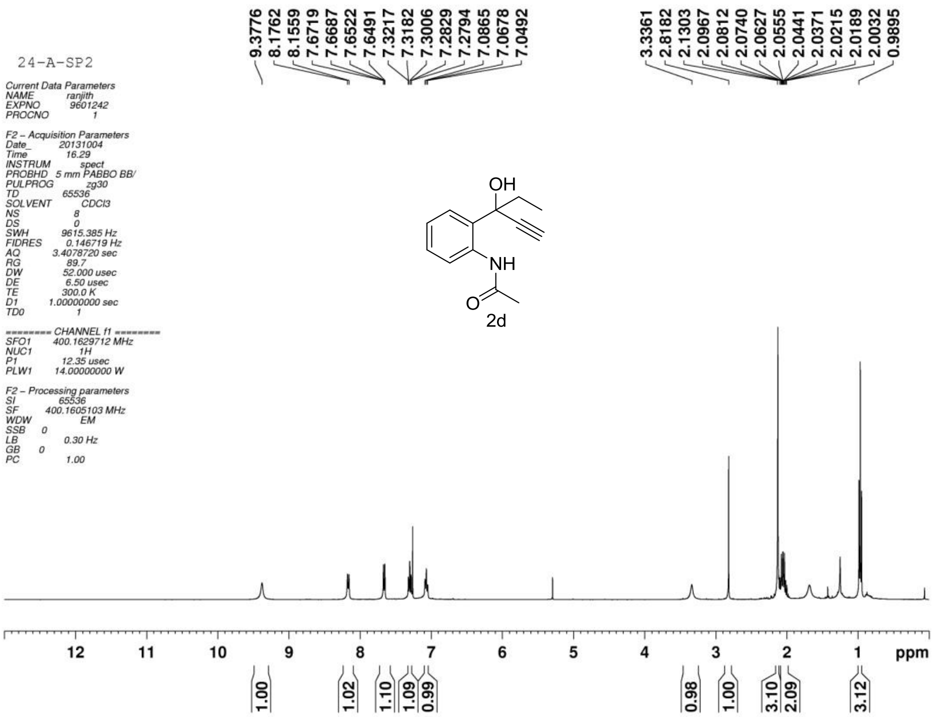
24-A-SP2
Current Data Parameters
NAME ranjith
EXPNO 9601242
PROCNO 1
F2 - Acquisition Parameters
Date_ 20131004
Time 16.29
INSTRUM spect
PROBHD 5 mm PABBO BB/
PULPROG zg30
TD 65536
SOLVENT CDCl3
NS 8
DS 0
SWH 9615.385 Hz
FIDRES 0.146719 Hz
AQ 3.4078720 sec
RG 89.7
DW 52.000 usec
DE 6.50 usec
TE 300.0 K
D1 1.00000000 sec
TD0 1
======== CHANNEL f1 ========
SFO1 400.1629712 MHz
NUC1 1H
P1 12.35 usec
PLW1 14.00000000 W
F2 - Processing parameters
SI 65536
SF 400.1605103 MHz
WDW EM
SSB 0
LB 0.30 Hz
GB 0
PC 1.00
9.3776
8.1762
8.1559
7.6719
7.6687
7.6522
7.6491
7.3217
7.3182
7.3006
7.2829
7.2794
7.0865
7.0678
7.0492
3.3361
2.8182
2.1303
2.0967
2.0812
2.0740
2.0627
2.0555
2.0441
2.0371
2.0215
2.0189
2.0032
0.9895
OH
NH
O
2d
12
11
10
9
8
7
6
5
4
3
2
1
ppm
1.00
1.02
1.10
1.09
0.99
0.98
1.00
3.10
2.09
3.12

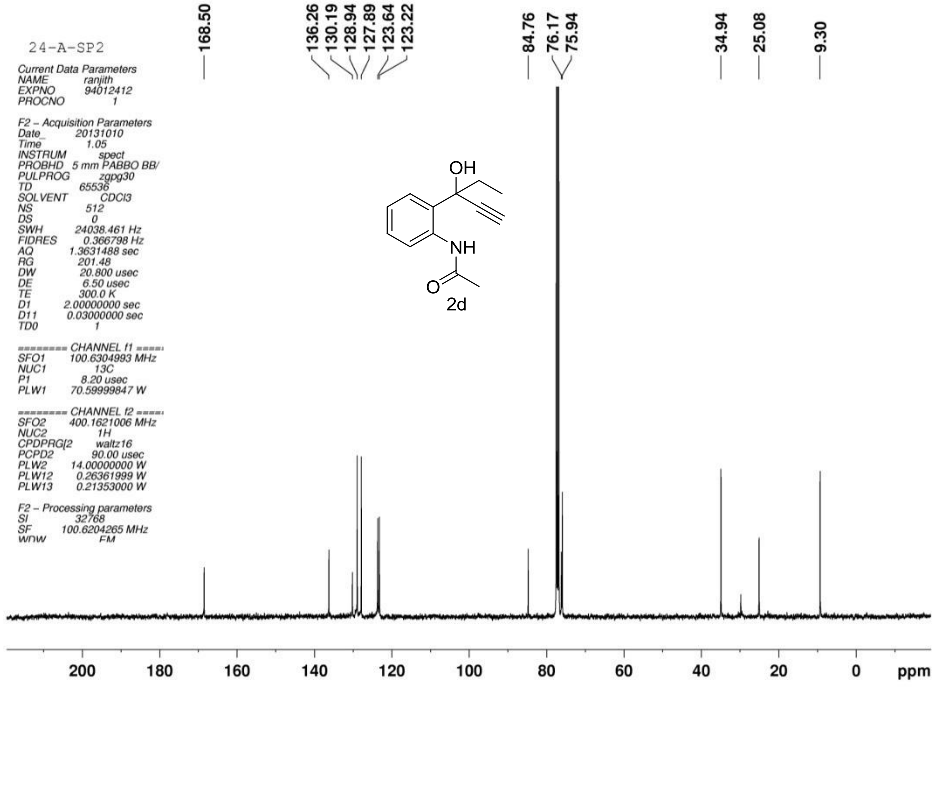
24-A-SP2
Current Data Parameters
NAME ranjith
EXPNO 94012412
PROCNO 1
F2 - Acquisition Parameters
Date_ 20131010
Time 1.05
INSTRUM spect
PROBHD 5 mm PABBO BB/
PULPROG zgpg30
TD 65536
SOLVENT CDCl3
NS 512
DS 0
SWH 24038.461 Hz
FIDRES 0.366798 Hz
AQ 1.3631488 sec
RG 201.48
DW 20.800 usec
DE 6.50 usec
TE 300.0 K
D1 2.00000000 sec
D11 0.03000000 sec
TD0 1
======== CHANNEL f1 ========
SFO1 100.6304993 MHz
NUC1 13C
P1 8.20 usec
PLW1 70.59999847 W
======== CHANNEL f2 ========
SFO2 400.1621006 MHz
NUC2 1H
CPDPRG[2 waltz16
PCPD2 90.00 usec
PLW2 14.00000000 W
PLW12 0.26361999 W
PLW13 0.21353000 W
F2 - Processing parameters
SI 32768
SF 100.6204265 MHz
168.50
136.26
130.19
128.94
127.89
123.64
123.22
84.76
76.17
75.94
34.94
25.08
9.30
OH
NH
O
2d
200
180
160
140
120
100
80
60
40
20
0
ppm

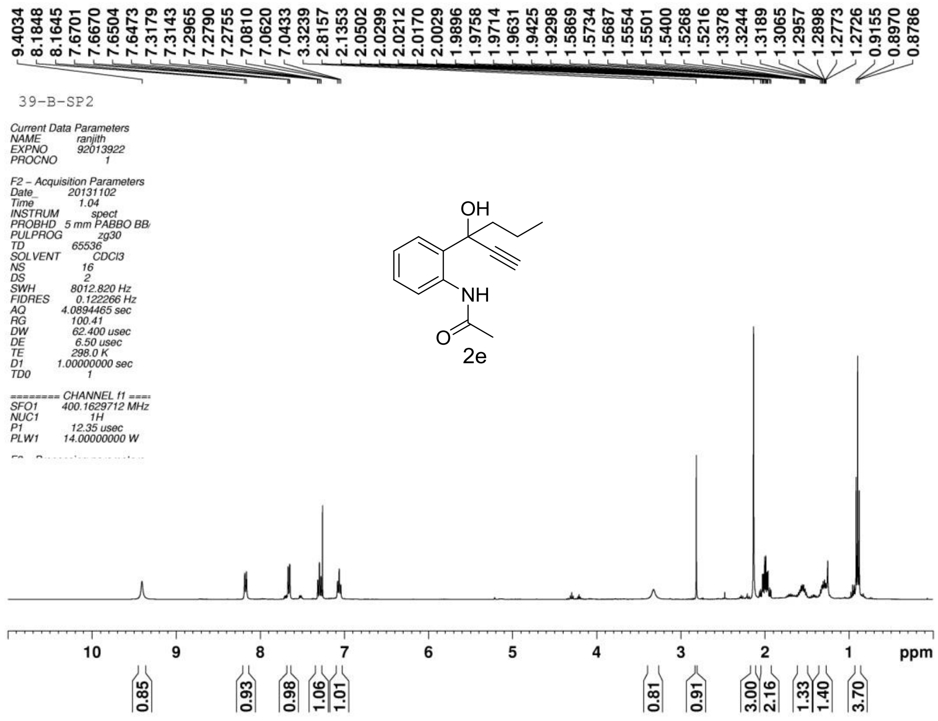
9.4034
8.1848
8.1645
7.6701
7.6670
7.6504
7.6473
7.3179
7.3143
7.2965
7.2790
7.2755
7.0810
7.0620
7.0433
3.3239
2.8157
2.1353
2.0502
2.0299
2.0212
2.0170
2.0029
1.9896
1.9758
1.9714
1.9631
1.9425
1.9298
1.5869
1.5734
1.5687
1.5554
1.5501
1.5400
1.5268
1.5216
1.3378
1.3244
1.3189
1.3065
1.2957
1.2898
1.2773
1.2726
0.9155
0.8970
0.8786
39-B-SP2
Current Data Parameters
NAME ranjith
EXPNO 92013922
PROCNO 1
F2 – Acquisition Parameters
Date_ 20131102
Time 1.04
INSTRUM spect
PULPROG zg30
TD 65536
SOLVENT CDCl3
NS 16
DS 2
SWH 8012.820 Hz
FIDRES 0.122266 Hz
AQ 4.0894465 sec
RG 100.41
DW 62.400 usec
DE 6.50 usec
TE 298.0 K
D1 1.00000000 sec
TD0 1
SFO1 400.1629712 MHz
NUC1 1H
P1 12.35 usec
PLW1 14.00000000 W
OH
NH
O
2e
10
9
8
7
6
5
4
3
2
1
ppm
0.85
0.93
0.98
1.06
1.01
0.81
0.91
3.00
2.16
1.33
1.40
3.70

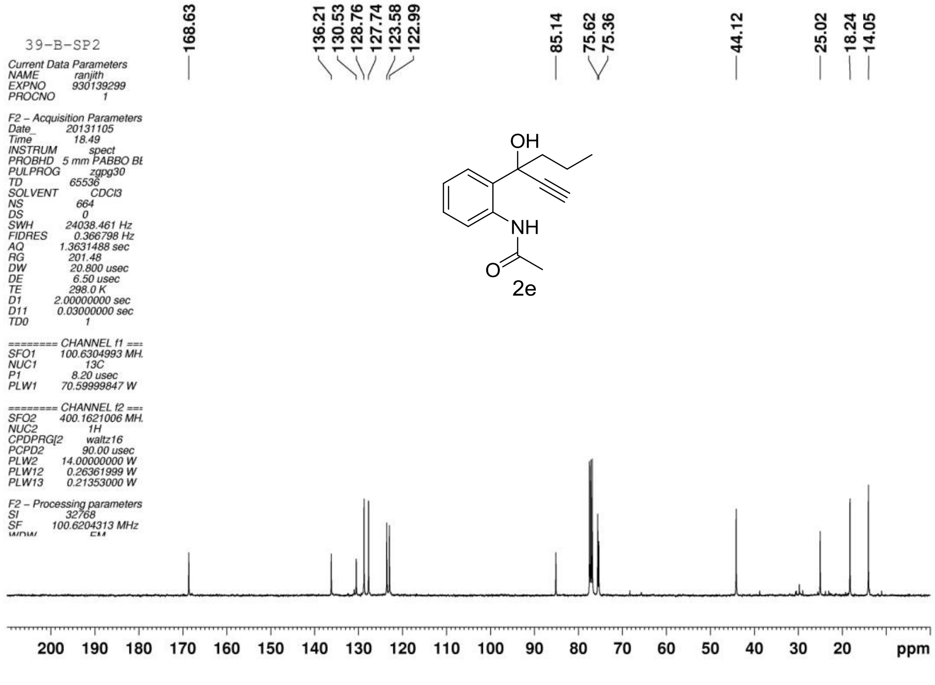
168.63
136.21
130.53
128.76
127.74
123.58
122.99
85.14
75.62
75.36
44.12
25.02
18.24
14.05
39-B-SP2
Current Data Parameters
NAME ranjith
EXPNO 930139299
PROCNO 1
F2 – Acquisition Parameters
Date_ 20131105
Time 18.49
INSTRUM spect
PULPROG zgpg30
TD 65536
SOLVENT CDCl3
NS 664
DS 0
SWH 24038.461 Hz
FIDRES 0.366798 Hz
AQ 1.3631488 sec
RG 201.48
DW 20.800 usec
DE 6.50 usec
TE 298.0 K
D1 2.00000000 sec
D11 0.03000000 sec
TD0 1
NUC1 13C
P1 8.20 usec
PLW1 70.59999847 W
NUC2 1H
CPDPRG[2 waltz16
PCPD2 90.00 usec
PLW2 14.00000000 W
PLW12 0.26361999 W
PLW13 0.21353000 W
F2 – Processing parameters
SI 32768
SF 100.6204313 MHz
OH
NH
O
2e
200
190
180
170
160
150
140
130
120
110
100
90
80
70
60
50
40
30
20
ppm

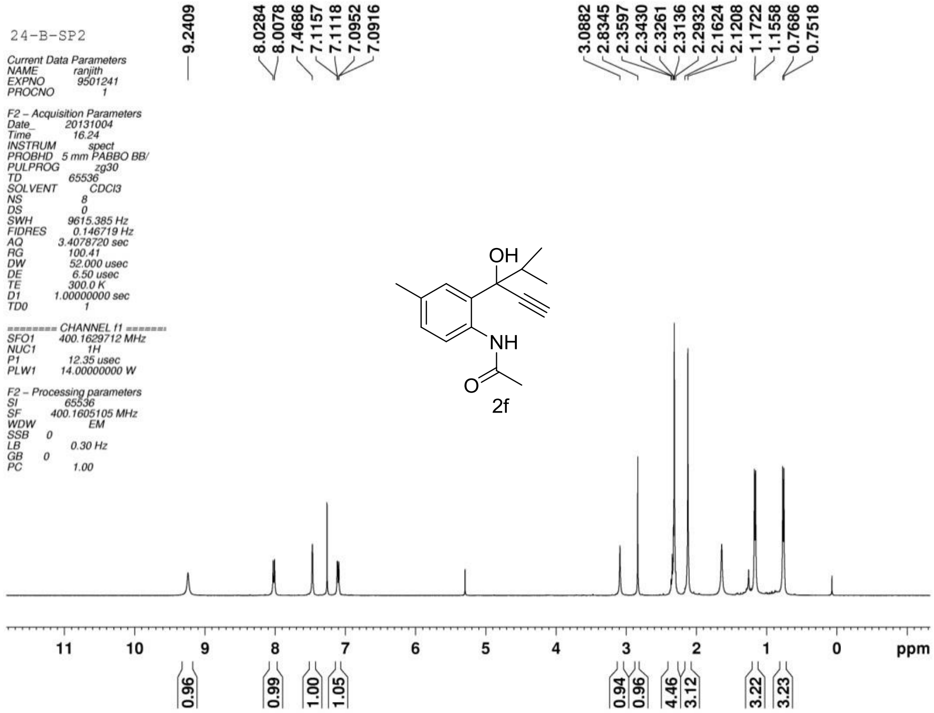
24-B-SP2
Current Data Parameters
NAME ranjith
EXPNO 9501241
PROCNO 1
F2 – Acquisition Parameters
Date_ 20131004
Time 16.24
INSTRUM spect
PROBHD 5 mm PABBO BB/
PULPROG zg30
TD 65536
SOLVENT CDCl3
NS 8
DS 0
SWH 9615.385 Hz
FIDRES 0.146719 Hz
AQ 3.4078720 sec
RG 100.41
DW 52.000 usec
DE 6.50 usec
TE 300.0 K
D1 1.00000000 sec
TD0 1
======= CHANNEL f1 ======
SFO1 400.1629712 MHz
NUC1 1H
P1 12.35 usec
PLW1 14.00000000 W
F2 – Processing parameters
SI 65536
SF 400.1605105 MHz
WDW EM
SSB 0
LB 0.30 Hz
GB 0
PC 1.00
9.2409
8.0284
8.0078
7.4686
7.1157
7.1118
7.0952
7.0916
3.0882
2.8345
2.3597
2.3430
2.3261
2.3136
2.2932
2.1624
2.1208
1.1722
1.1558
0.7686
0.7518
OH
NH
O
2f
11 10 9 8 7 6 5 4 3 2 1 0 ppm
0.96
0.99
1.00
1.05
0.94
0.96
4.46
3.12
3.22
3.23

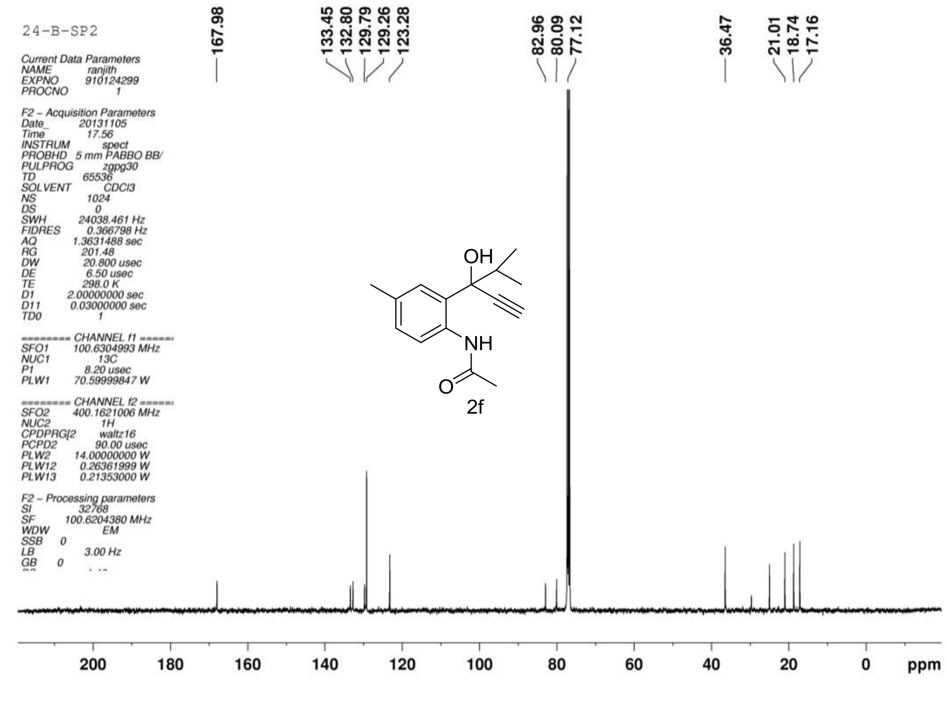
24-B-SP2
Current Data Parameters
NAME ranjith
EXPNO 910124299
PROCNO 1
F2 – Acquisition Parameters
Date_ 20131105
Time 17.56
INSTRUM spect
PROBHD 5 mm PABBO BB/
PULPROG zgpg30
TD 65536
SOLVENT CDCl3
NS 1024
DS 0
SWH 24038.461 Hz
FIDRES 0.366798 Hz
AQ 1.3631488 sec
RG 201.48
DW 20.800 usec
DE 6.50 usec
TE 298.0 K
D1 2.00000000 sec
D11 0.03000000 sec
TD0 1
======= CHANNEL f1 ======
SFO1 100.6304993 MHz
NUC1 13C
P1 8.20 usec
PLW1 70.59999847 W
======= CHANNEL f2 ======
SFO2 400.1621006 MHz
NUC2 1H
CPDPRG[2 waltz16
PCPD2 90.00 usec
PLW2 14.00000000 W
PLW12 0.26361999 W
PLW13 0.21353000 W
F2 – Processing parameters
SI 32768
SF 100.6204380 MHz
WDW EM
SSB 0
LB 3.00 Hz
GB 0
167.98
133.45
132.80
129.79
129.26
123.28
82.96
80.09
77.12
36.47
21.01
18.74
17.16
OH
NH
O
2f
200 180 160 140 120 100 80 60 40 20 0 ppm

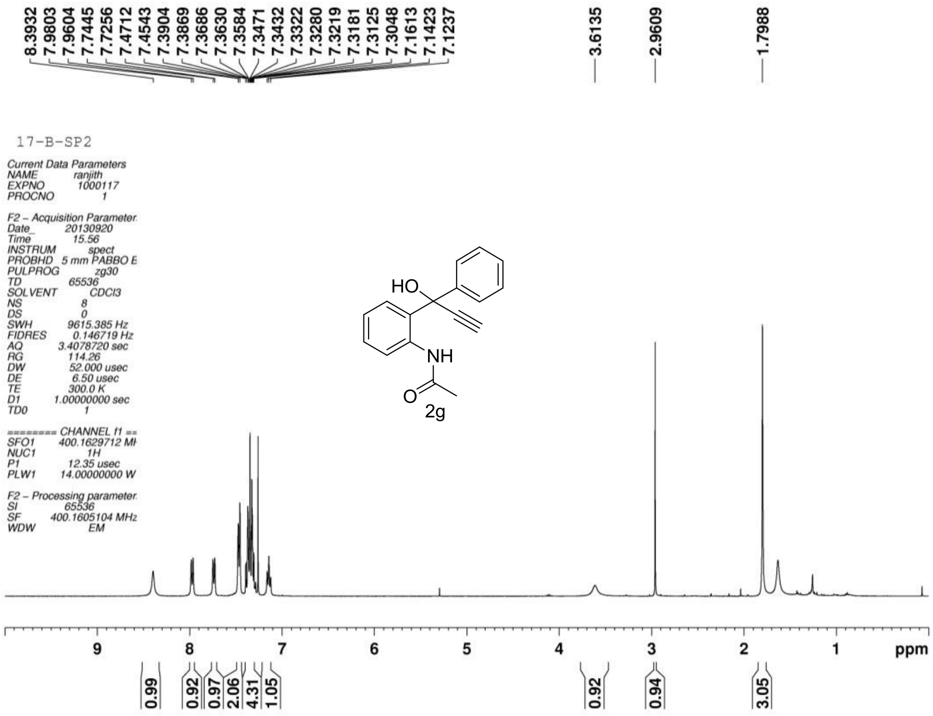
8.3932
7.9803
7.9604
7.7445
7.7256
7.4712
7.4543
7.3904
7.3869
7.3686
7.3630
7.3584
7.3471
7.3432
7.3322
7.3280
7.3219
7.3181
7.3125
7.3048
7.1613
7.1423
7.1237
3.6135
2.9609
1.7988
17-B-SP2
Current Data Parameters
NAME ranjith
EXPNO 1000117
PROCNO 1
F2 – Acquisition Parameter
Date_ 20130920
Time 15.56
INSTRUM spect
PROBHD 5 mm PABBO E
PULPROG zg30
TD 65536
SOLVENT CDCl3
NS 8
DS 0
SWH 9615.385 Hz
FIDRES 0.146719 Hz
AQ 3.4078720 sec
RG 114.26
DW 52.000 usec
DE 6.50 usec
TE 300.0 K
D1 1.00000000 sec
TD0 1
======== CHANNEL f1 ==
SFO1 400.1629712 MH
NUC1 1H
P1 12.35 usec
PLW1 14.00000000 W
F2 – Processing parameter
SI 65536
SF 400.1605104 MHz
WDW EM
HO
NH
O
2g
9
8
7
6
5
4
3
2
1
ppm
0.99
0.92
0.97
2.06
4.31
1.05
0.92
0.94
3.05

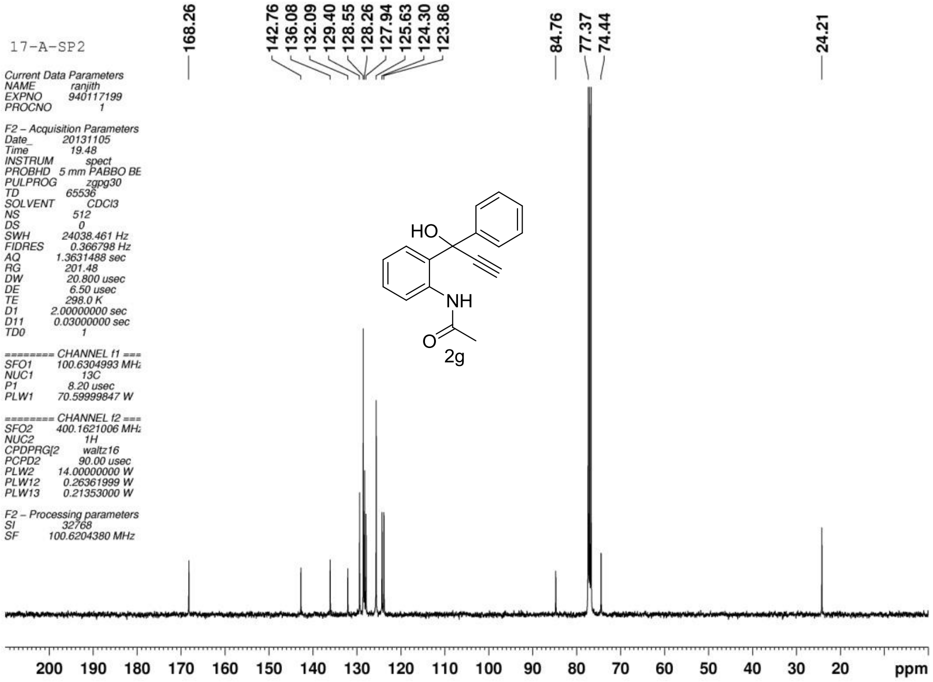
168.26
142.76
136.08
132.09
129.40
128.55
128.26
127.94
125.63
124.30
123.86
84.76
77.37
74.44
24.21
17-A-SP2
Current Data Parameters
NAME ranjith
EXPNO 940117199
PROCNO 1
F2 – Acquisition Parameters
Date_ 20131105
Time 19.48
INSTRUM spect
PROBHD 5 mm PABBO BE
PULPROG zgpg30
TD 65536
SOLVENT CDCl3
NS 512
DS 0
SWH 24038.461 Hz
FIDRES 0.366798 Hz
AQ 1.3631488 sec
RG 201.48
DW 20.800 usec
DE 6.50 usec
TE 298.0 K
D1 2.00000000 sec
D11 0.03000000 sec
TD0 1
======== CHANNEL f1 ===
SFO1 100.6304993 MHz
NUC1 13C
P1 8.20 usec
PLW1 70.59999847 W
======== CHANNEL f2 ===
SFO2 400.1621006 MHz
NUC2 1H
CPDPRG[2 waltz16
PCPD2 90.00 usec
PLW2 14.00000000 W
PLW12 0.26361999 W
PLW13 0.21353000 W
F2 – Processing parameters
SI 32768
SF 100.6204380 MHz
HO
NH
O
2g
200
190
180
170
160
150
140
130
120
110
100
90
80
70
60
50
40
30
20
ppm

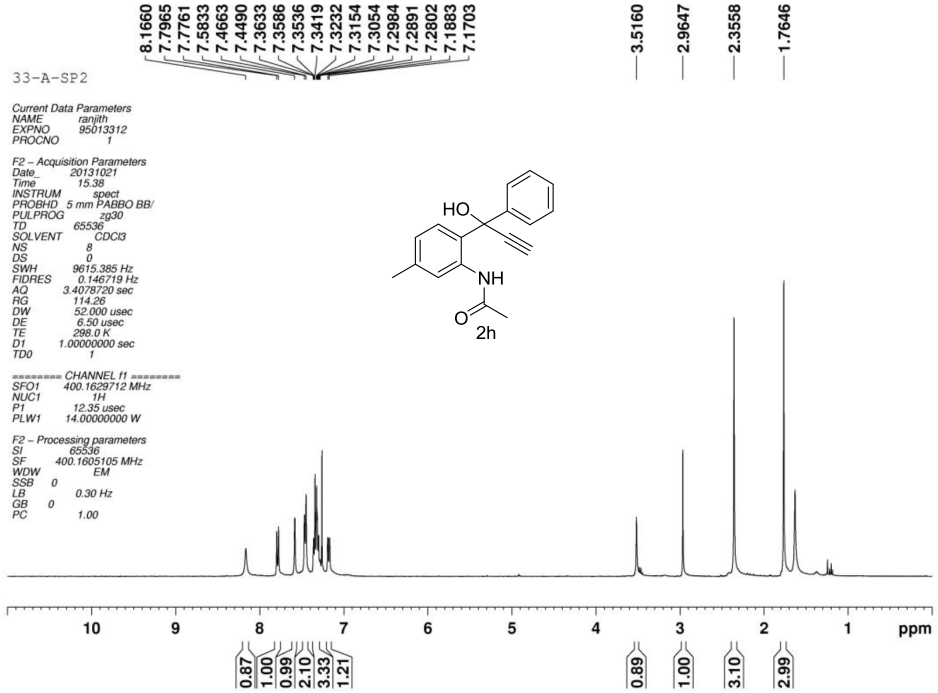
8.1660
7.7965
7.7761
7.5833
7.4663
7.4490
7.3633
7.3586
7.3536
7.3419
7.3232
7.3154
7.3054
7.2984
7.2891
7.2802
7.1883
7.1703
3.5160
2.9647
2.3558
1.7646
33-A-SP2
Current Data Parameters
NAME ranjith
EXPNO 95013312
PROCNO 1
F2 – Acquisition Parameters
Date_ 20131021
Time 15.38
INSTRUM spect
PROBHD 5 mm PABBO BB/
PULPROG zg30
TD 65536
SOLVENT CDCl3
NS 8
DS 0
SWH 9615.385 Hz
FIDRES 0.146719 Hz
AQ 3.4078720 sec
RG 114.26
DW 52.000 usec
DE 6.50 usec
TE 298.0 K
D1 1.00000000 sec
TD0 1
======== CHANNEL f1 ========
SFO1 400.1629712 MHz
NUC1 1H
P1 12.35 usec
PLW1 14.00000000 W
F2 – Processing parameters
SI 65536
SF 400.1605105 MHz
WDW EM
SSB 0
LB 0.30 Hz
GB 0
PC 1.00
HO
NH
O
2h
10
9
8
7
6
5
4
3
2
1
ppm
0.87
1.00
0.99
2.10
3.33
1.21
0.89
1.00
3.10
2.99

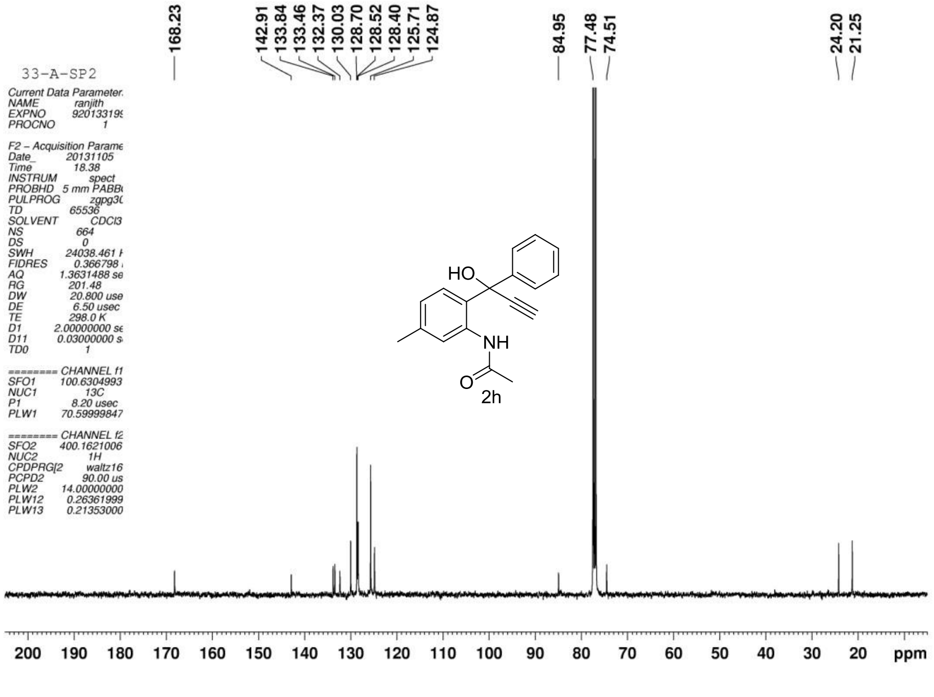
168.23
142.91
133.84
133.46
132.37
130.03
128.70
128.52
128.40
125.71
124.87
84.95
77.48
74.51
24.20
21.25
33-A-SP2
Current Data Parameter
NAME ranjith
EXPNO 92013319
PROCNO 1
F2 – Acquisition Parame
Date_ 20131105
Time 18.38
INSTRUM spect
PROBHD 5 mm PABB
PULPROG zgpg30
TD 65536
SOLVENT CDCl3
NS 664
DS 0
SWH 24038.461
FIDRES 0.366798
AQ 1.3631488 se
RG 201.48
DW 20.800 use
DE 6.50 usec
TE 298.0 K
D1 2.00000000 se
D11 0.03000000 s
TD0 1
======== CHANNEL f1
SFO1 100.6304993
NUC1 13C
P1 8.20 usec
PLW1 70.59999847
======== CHANNEL f2
SFO2 400.1621006
NUC2 1H
CPDPRG[2 waltz16
PCPD2 90.00 us
PLW2 14.00000000
PLW12 0.26361999
PLW13 0.21353000
HO
NH
O
2h
200
190
180
170
160
150
140
130
120
110
100
90
80
70
60
50
40
30
20
ppm

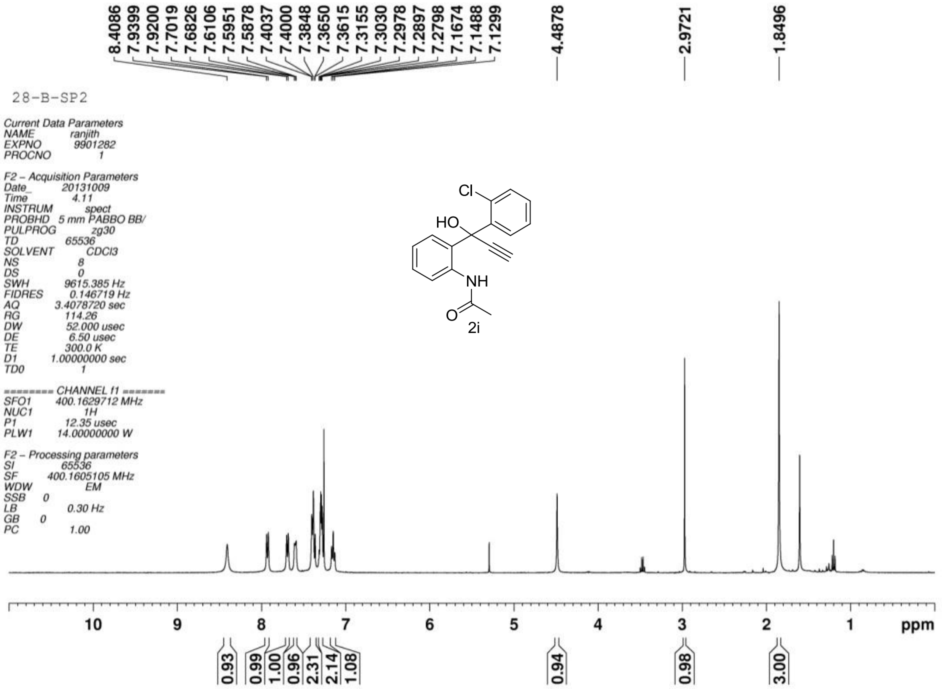
8.4086
7.9399
7.9200
7.7019
7.6826
7.6106
7.5951
7.5878
7.4037
7.4000
7.3848
7.3650
7.3615
7.3155
7.3030
7.2978
7.2897
7.2798
7.1674
7.1488
7.1299
4.4878
2.9721
1.8496
28-B-SP2
Current Data Parameters
NAME ranjith
EXPNO 9901282
PROCNO 1
F2 - Acquisition Parameters
Date_ 20131009
Time 4.11
INSTRUM spect
PROBHD 5 mm PABBO BB/
PULPROG zg30
TD 65536
SOLVENT CDCl3
NS 8
DS 0
SWH 9615.385 Hz
FIDRES 0.146719 Hz
AQ 3.4078720 sec
RG 114.26
DW 52.000 usec
DE 6.50 usec
TE 300.0 K
D1 1.00000000 sec
TD0 1
======== CHANNEL f1 ========
SFO1 400.1629712 MHz
NUC1 1H
P1 12.35 usec
PLW1 14.00000000 W
F2 - Processing parameters
SI 65536
SF 400.1605105 MHz
WDW EM
SSB 0
LB 0.30 Hz
GB 0
PC 1.00
Cl
HO
NH
O
2i
10
9
8
7
6
5
4
3
2
1
ppm
0.93
0.99
1.00
0.96
2.31
2.14
1.08
0.94
0.98
3.00

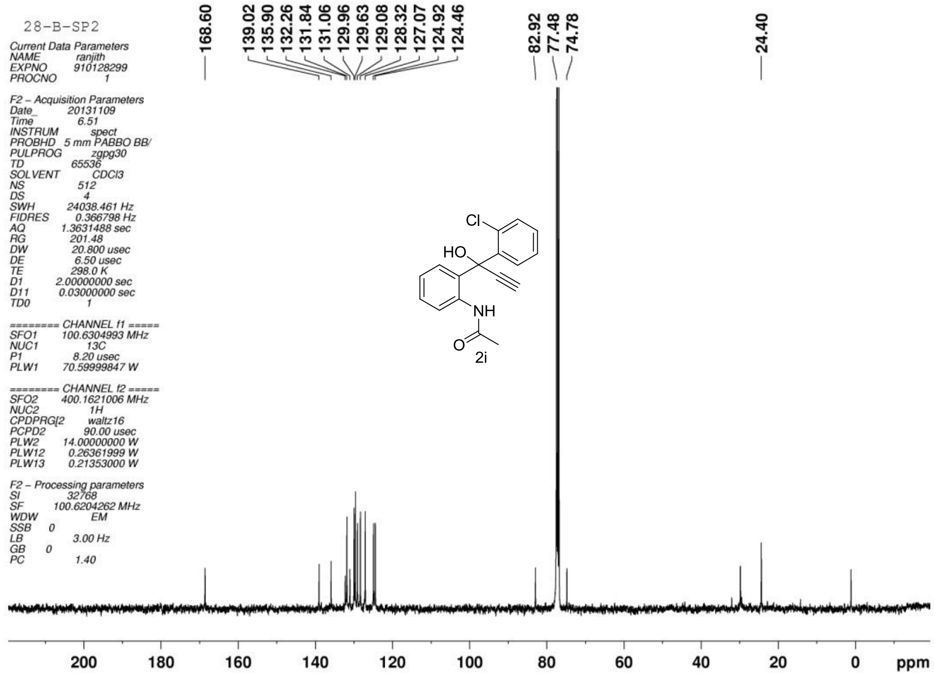
168.60
139.02
135.90
132.26
131.84
131.06
129.96
129.63
129.08
128.32
127.07
124.92
124.46
82.92
77.48
74.78
24.40
28-B-SP2
Current Data Parameters
NAME ranjith
EXPNO 910128299
PROCNO 1
F2 - Acquisition Parameters
Date_ 20131109
Time 6.51
INSTRUM spect
PROBHD 5 mm PABBO BB/
PULPROG zgpg30
TD 65536
SOLVENT CDCl3
NS 512
DS 4
SWH 24038.461 Hz
FIDRES 0.366798 Hz
AQ 1.3631488 sec
RG 201.48
DW 20.800 usec
DE 6.50 usec
TE 298.0 K
D1 2.00000000 sec
D11 0.03000000 sec
TD0 1
======== CHANNEL f1 =====
SFO1 100.6304993 MHz
NUC1 13C
P1 8.20 usec
PLW1 70.59999847 W
======== CHANNEL f2 =====
SFO2 400.1621006 MHz
NUC2 1H
CPDPRG[2 waltz16
PCPD2 90.00 usec
PLW2 14.00000000 W
PLW12 0.26361999 W
PLW13 0.21353000 W
F2 - Processing parameters
SI 32768
SF 100.6204262 MHz
WDW EM
SSB 0
LB 3.00 Hz
GB 0
PC 1.40
Cl
HO
NH
O
2i
200
180
160
140
120
100
80
60
40
20
0
ppm

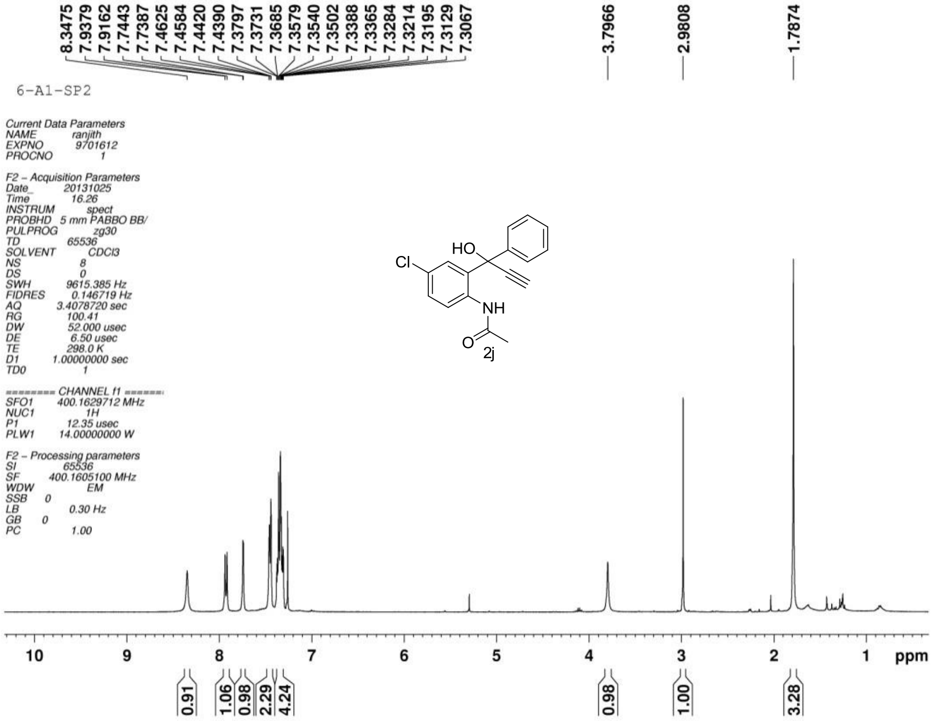
8.3475
7.9379
7.9162
7.7443
7.7387
7.4625
7.4584
7.4420
7.4390
7.3797
7.3731
7.3685
7.3579
7.3540
7.3502
7.3388
7.3365
7.3284
7.3214
7.3195
7.3129
7.3067
3.7966
2.9808
1.7874
6-A1-SP2
Current Data Parameters
NAME ranjith
EXPNO 9701612
PROCNO 1
F2 - Acquisition Parameters
Date_ 20131025
Time 16.26
INSTRUM spect
PROBHD 5 mm PABBO BB/
PULPROG zg30
TD 65536
SOLVENT CDCl3
NS 8
DS 0
SWH 9615.385 Hz
FIDRES 0.146719 Hz
AQ 3.4078720 sec
RG 100.41
DW 52.000 usec
DE 6.50 usec
TE 298.0 K
D1 1.00000000 sec
TD0 1
======= CHANNEL f1 ======
SFO1 400.1629712 MHz
NUC1 1H
P1 12.35 usec
PLW1 14.00000000 W
F2 - Processing parameters
SI 65536
SF 400.1605100 MHz
WDW EM
SSB 0
LB 0.30 Hz
GB 0
PC 1.00
Cl
HO
NH
O
2j
10
9
8
7
6
5
4
3
2
1
ppm
0.91
1.06
0.98
2.29
4.24
0.98
1.00
3.28

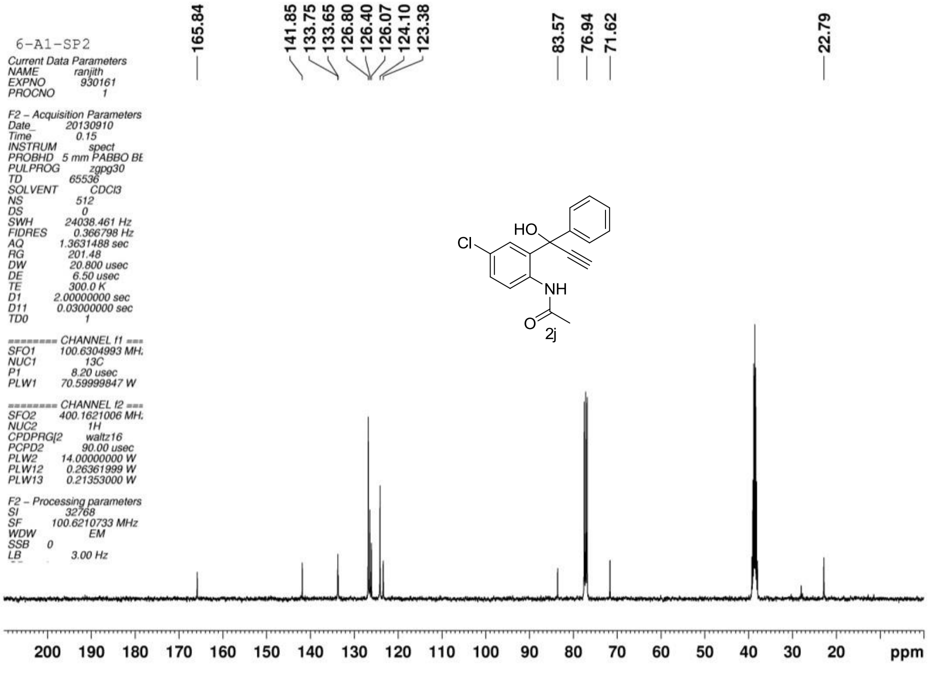
165.84
141.85
133.75
133.65
126.80
126.40
126.07
124.10
123.38
83.57
76.94
71.62
22.79
6-A1-SP2
Current Data Parameters
NAME ranjith
EXPNO 930161
PROCNO 1
F2 - Acquisition Parameters
Date_ 20130910
Time 0.15
INSTRUM spect
PROBHD 5 mm PABBO BE
PULPROG zgpg30
TD 65536
SOLVENT CDCl3
NS 512
DS 0
SWH 24038.461 Hz
FIDRES 0.366798 Hz
AQ 1.3631488 sec
RG 201.48
DW 20.800 usec
DE 6.50 usec
TE 300.0 K
D1 2.00000000 sec
D11 0.03000000 sec
TD0 1
======= CHANNEL f1 ===
SFO1 100.6304993 MH:
NUC1 13C
P1 8.20 usec
PLW1 70.59999847 W
======= CHANNEL f2 ===
SFO2 400.1621006 MH:
NUC2 1H
CPDPRG[2 waltz16
PCPD2 90.00 usec
PLW2 14.00000000 W
PLW12 0.26361999 W
PLW13 0.21353000 W
F2 - Processing parameters
SI 32768
SF 100.6210733 MHz
WDW EM
SSB 0
LB 3.00 Hz
Cl
HO
NH
O
2j
200
190
180
170
160
150
140
130
120
110
100
90
80
70
60
50
40
30
20
ppm

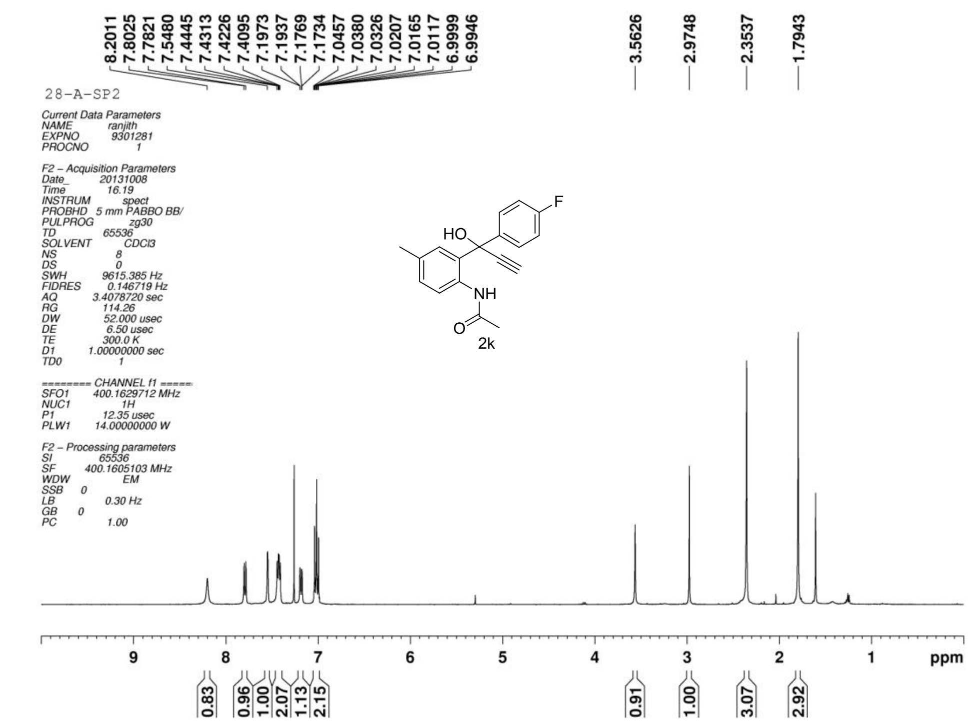
8.2011
7.8025
7.7821
7.5480
7.4445
7.4313
7.4226
7.4095
7.1973
7.1937
7.1769
7.1734
7.0457
7.0380
7.0326
7.0207
7.0165
7.0117
6.9999
6.9946
3.5626
2.9748
2.3537
1.7943
28-A-SP2
Current Data Parameters
NAME ranjith
EXPNO 9301281
PROCNO 1
F2 – Acquisition Parameters
Date_ 20131008
Time 16.19
INSTRUM spect
PROBHD 5 mm PABBO BB/
PULPROG zg30
TD 65536
SOLVENT CDCl3
NS 8
DS 0
SWH 9615.385 Hz
FIDRES 0.146719 Hz
AQ 3.4078720 sec
RG 114.26
DW 52.000 usec
DE 6.50 usec
TE 300.0 K
D1 1.00000000 sec
TD0 1
======= CHANNEL f1 =====
SFO1 400.1629712 MHz
NUC1 1H
P1 12.35 usec
PLW1 14.00000000 W
F2 – Processing parameters
SI 65536
SF 400.1605103 MHz
WDW EM
SSB 0
LB 0.30 Hz
GB 0
PC 1.00
HO
F
NH
O
2k
9
8
7
6
5
4
3
2
1
ppm
0.83
0.96
1.00
2.07
1.13
2.15
0.91
1.00
3.07
2.92

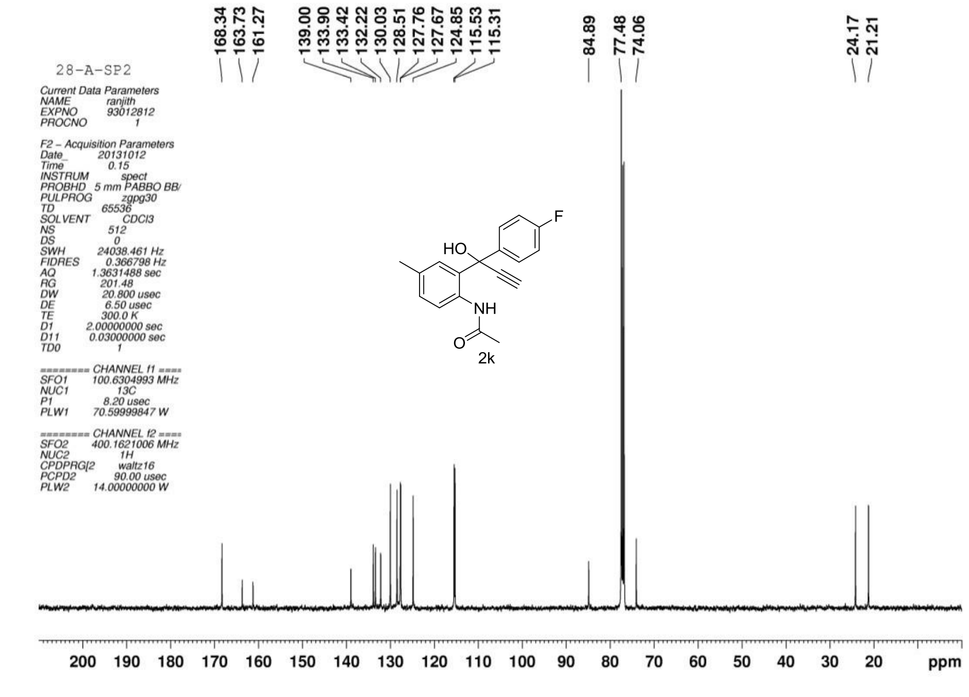
168.34
163.73
161.27
139.00
133.90
133.42
132.22
130.03
128.51
127.76
127.67
124.85
115.53
115.31
84.89
77.48
74.06
24.17
21.21
28-A-SP2
Current Data Parameters
NAME ranjith
EXPNO 93012812
PROCNO 1
F2 – Acquisition Parameters
Date_ 20131012
Time 0.15
INSTRUM spect
PROBHD 5 mm PABBO BB/
PULPROG zgpg30
TD 65536
SOLVENT CDCl3
NS 512
DS 0
SWH 24038.461 Hz
FIDRES 0.366798 Hz
AQ 1.3631488 sec
RG 201.48
DW 20.800 usec
DE 6.50 usec
TE 300.0 K
D1 2.00000000 sec
D11 0.03000000 sec
TD0 1
======= CHANNEL f1 ====
SFO1 100.6304993 MHz
NUC1 13C
P1 8.20 usec
PLW1 70.59999847 W
======= CHANNEL f2 ====
SFO2 400.1621006 MHz
NUC2 1H
CPDPRG[2 waltz16
PCPD2 90.00 usec
PLW2 14.00000000 W
HO
F
NH
O
2k
200
190
180
170
160
150
140
130
120
110
100
90
80
70
60
50
40
30
20
ppm

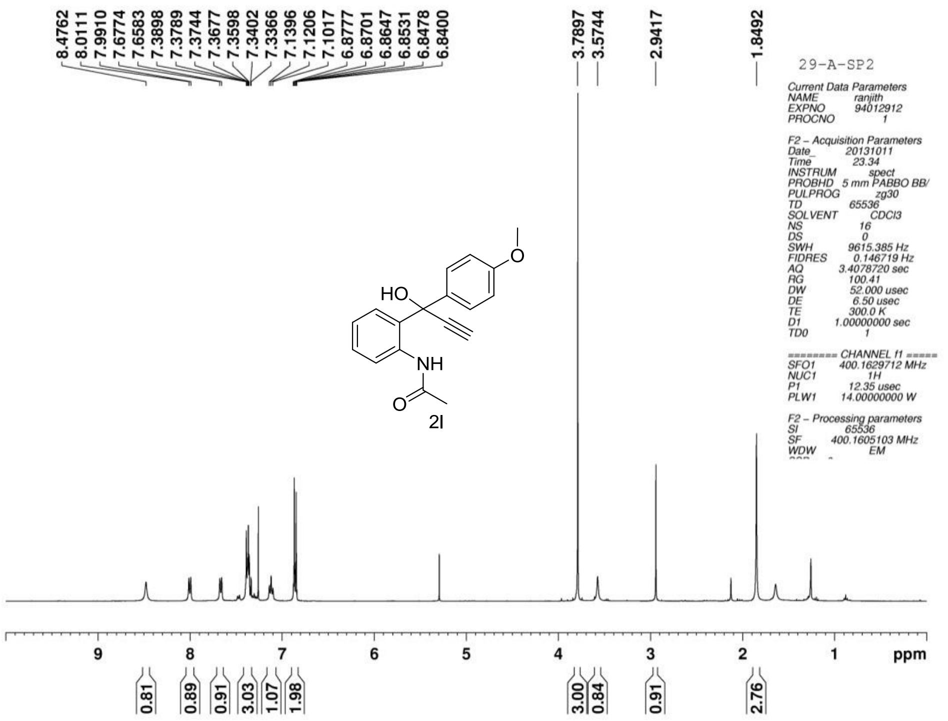
8.4762
8.0111
7.9910
7.6774
7.6583
7.3898
7.3789
7.3744
7.3677
7.3598
7.3402
7.3366
7.1396
7.1206
7.1017
6.8777
6.8701
6.8647
6.8531
6.8478
6.8400
3.7897
3.5744
2.9417
1.8492
29-A-SP2
Current Data Parameters
NAME ranjith
EXPNO 94012912
PROCNO 1
F2 - Acquisition Parameters
Date_ 20131011
Time 23.34
INSTRUM spect
PROBHD 5 mm PABBO BB/
PULPROG zg30
TD 65536
SOLVENT CDCl3
NS 16
DS 0
SWH 9615.385 Hz
FIDRES 0.146719 Hz
AQ 3.4078720 sec
RG 100.41
DW 52.000 usec
DE 6.50 usec
TE 300.0 K
D1 1.00000000 sec
TD0 1
======== CHANNEL f1 ======
SFO1 400.1629712 MHz
NUC1 1H
P1 12.35 usec
PLW1 14.00000000 W
F2 - Processing parameters
SI 65536
SF 400.1605103 MHz
WDW EM
HO
NH
O
2l
ppm
0.81
0.89
0.91
3.03
1.07
1.98
3.00
0.84
0.91
2.76

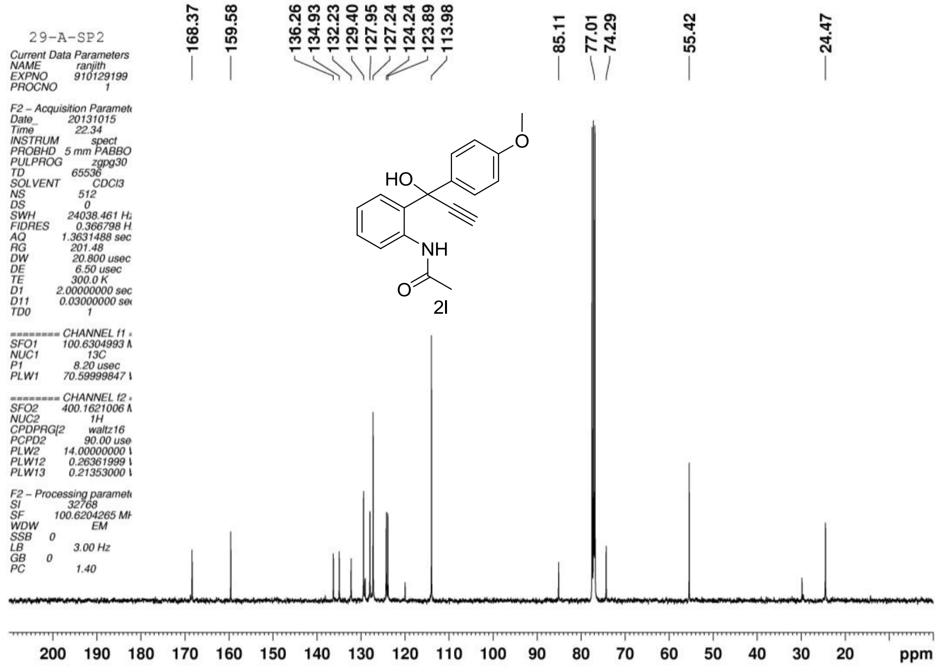
168.37
159.58
136.26
134.93
132.23
129.40
127.95
127.24
124.24
123.89
113.98
85.11
77.01
74.29
55.42
24.47
29-A-SP2
Current Data Parameters
NAME ranjith
EXPNO 910129199
PROCNO 1
F2 - Acquisition Paramete
Date_ 20131015
Time 22.34
INSTRUM spect
PROBHD 5 mm PABBO
PULPROG zgpg30
TD 65536
SOLVENT CDCl3
NS 512
DS 0
SWH 24038.461 Hz
FIDRES 0.366798 H
AQ 1.3631488 sec
RG 201.48
DW 20.800 usec
DE 6.50 usec
TE 300.0 K
D1 2.00000000 sec
D11 0.03000000 sec
TD0 1
======== CHANNEL f1
SFO1 100.6304993 M
NUC1 13C
P1 8.20 usec
PLW1 70.59999847 W
======== CHANNEL f2
SFO2 400.1621006 M
NUC2 1H
CPDPRG[2 waltz16
PCPD2 90.00 use
PLW2 14.00000000 W
PLW12 0.26361999 W
PLW13 0.21353000 W
F2 - Processing paramete
SI 32768
SF 100.6204265 MH
WDW EM
SSB 0
LB 3.00 Hz
GB 0
PC 1.40
HO
NH
O
2l
ppm

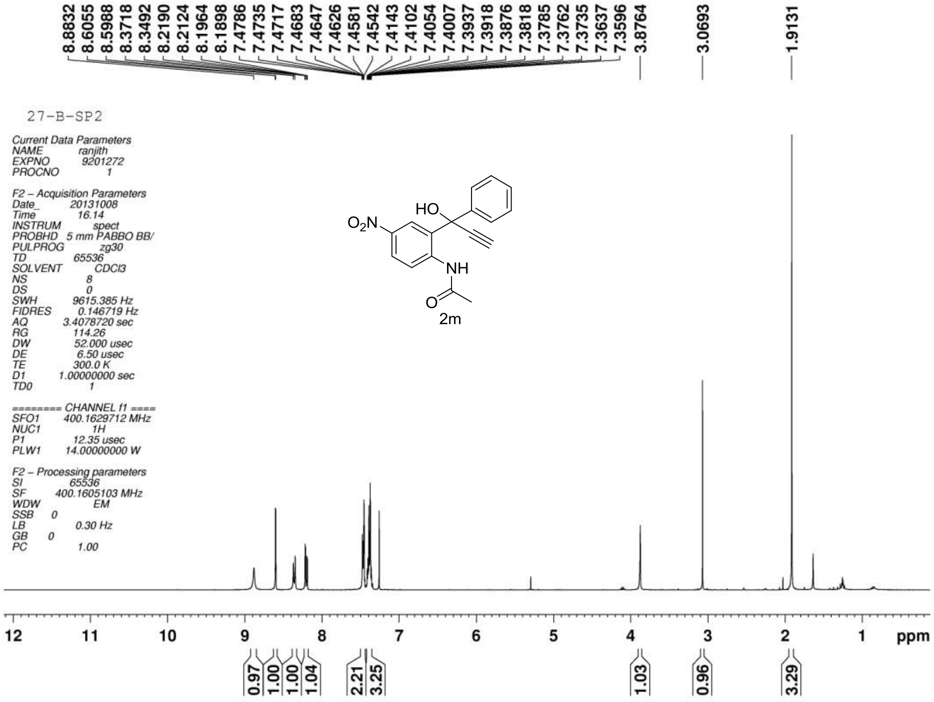
8.8832
8.6055
8.5988
8.3718
8.3492
8.2190
8.2124
8.1964
8.1898
7.4786
7.4735
7.4717
7.4683
7.4647
7.4626
7.4581
7.4542
7.4143
7.4102
7.4054
7.4007
7.3937
7.3918
7.3876
7.3818
7.3785
7.3762
7.3735
7.3637
7.3596
3.8764
3.0693
1.9131
27-B-SP2
Current Data Parameters
NAME ranjith
EXPNO 9201272
PROCNO 1
F2 - Acquisition Parameters
Date_ 20131008
Time 16.14
INSTRUM spect
PROBHD 5 mm PABBO BB/
PULPROG zg30
TD 65536
SOLVENT CDCl3
NS 8
DS 0
SWH 9615.385 Hz
FIDRES 0.146719 Hz
AQ 3.4078720 sec
RG 114.26
DW 52.000 usec
DE 6.50 usec
TE 300.0 K
D1 1.00000000 sec
TD0 1
======== CHANNEL f1 ====
SFO1 400.1629712 MHz
NUC1 1H
P1 12.35 usec
PLW1 14.00000000 W
F2 - Processing parameters
SI 65536
SF 400.1605103 MHz
WDW EM
SSB 0
LB 0.30 Hz
GB 0
PC 1.00
O2N
HO
NH
O
2m
12 11 10 9 8 7 6 5 4 3 2 1 ppm
0.97
1.00
1.00
1.04
2.21
3.25
1.03
0.96
3.29

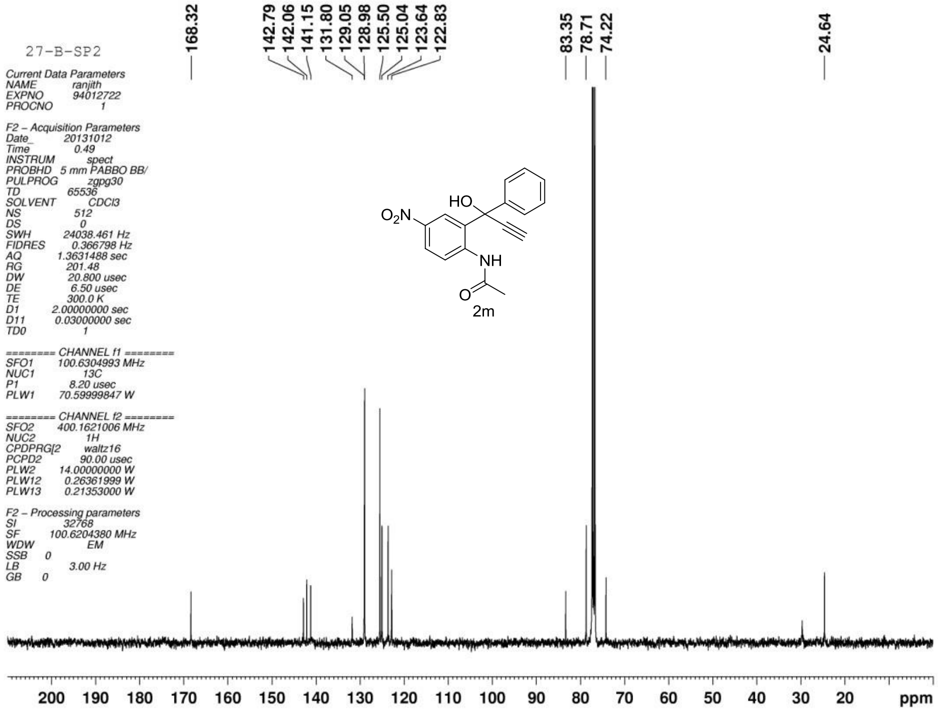
168.32
142.79
142.06
141.15
131.80
129.05
128.98
125.50
125.04
123.64
122.83
83.35
78.71
74.22
24.64
27-B-SP2
Current Data Parameters
NAME ranjith
EXPNO 94012722
PROCNO 1
F2 - Acquisition Parameters
Date_ 20131012
Time 0.49
INSTRUM spect
PROBHD 5 mm PABBO BB/
PULPROG zgpg30
TD 65536
SOLVENT CDCl3
NS 512
DS 0
SWH 24038.461 Hz
FIDRES 0.366798 Hz
AQ 1.3631488 sec
RG 201.48
DW 20.800 usec
DE 6.50 usec
TE 300.0 K
D1 2.00000000 sec
D11 0.03000000 sec
TD0 1
======== CHANNEL f1 ========
SFO1 100.6304993 MHz
NUC1 13C
P1 8.20 usec
PLW1 70.59999847 W
======== CHANNEL f2 ========
SFO2 400.1621006 MHz
NUC2 1H
CPDPRG[2 waltz16
PCPD2 90.00 usec
PLW2 14.00000000 W
PLW12 0.26361999 W
PLW13 0.21353000 W
F2 - Processing parameters
SI 32768
SF 100.6204380 MHz
WDW EM
SSB 0
LB 3.00 Hz
GB 0
O2N
HO
NH
O
2m
200 190 180 170 160 150 140 130 120 110 100 90 80 70 60 50 40 30 20 ppm

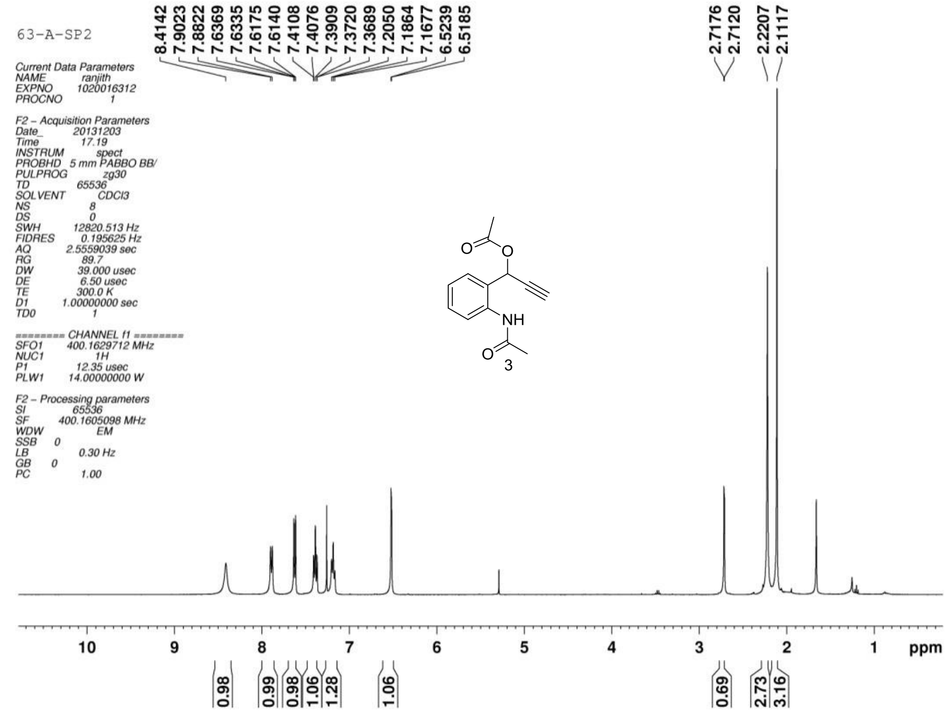
63-A-SP2
8.4142
7.9023
7.8822
7.6369
7.6335
7.6175
7.6140
7.4108
7.4076
7.3909
7.3720
7.3689
7.2050
7.1864
7.1677
6.5239
6.5185
2.7176
2.7120
2.2207
2.1117
Current Data Parameters
NAME ranjith
EXPNO 1020016312
PROCNO 1
F2 – Acquisition Parameters
Date_ 20131203
Time 17.19
INSTRUM spect
PROBHD 5 mm PABBO BB/
PULPROG zg30
TD 65536
SOLVENT CDCl3
NS 8
DS 0
SWH 12820.513 Hz
FIDRES 0.195625 Hz
AQ 2.5559039 sec
RG 89.7
DW 39.000 usec
DE 6.50 usec
TE 300.0 K
D1 1.00000000 sec
TD0 1
======= CHANNEL f1 =======
SFO1 400.1629712 MHz
NUC1 1H
P1 12.35 usec
PLW1 14.00000000 W
F2 – Processing parameters
SI 65536
SF 400.1605098 MHz
WDW EM
SSB 0
LB 0.30 Hz
GB 0
PC 1.00
NH
O
3
10
9
8
7
6
5
4
3
2
1
ppm
0.98
0.99
0.98
1.06
1.28
1.06
0.69
2.73
3.16

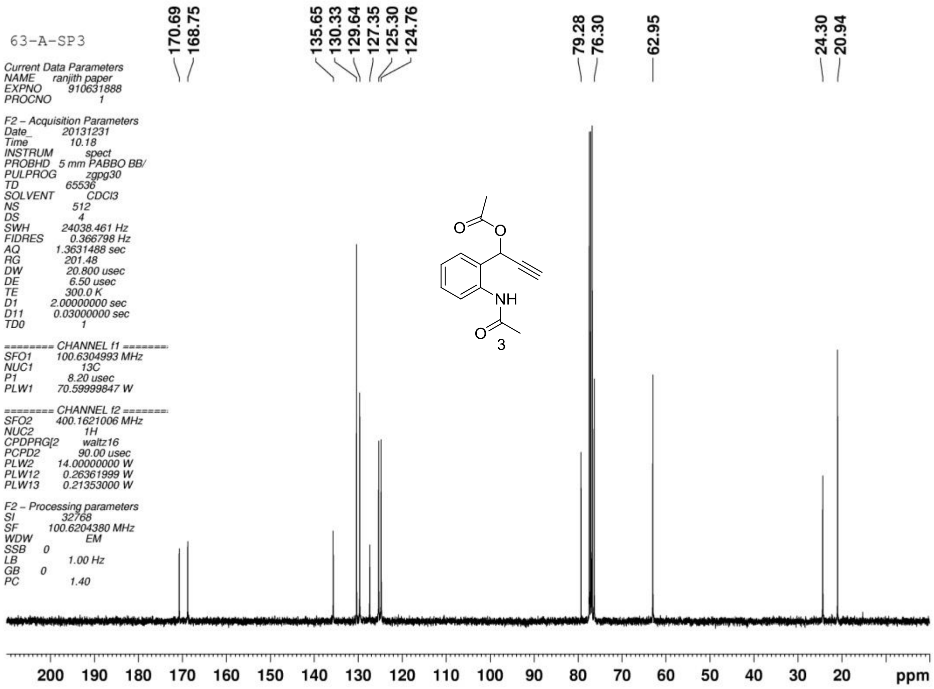
63-A-SP3
170.69
168.75
135.65
130.33
129.64
127.35
125.30
124.76
79.28
76.30
62.95
24.30
20.94
Current Data Parameters
NAME ranjith paper
EXPNO 910631888
PROCNO 1
F2 – Acquisition Parameters
Date_ 20131231
Time 10.18
INSTRUM spect
PROBHD 5 mm PABBO BB/
PULPROG zgpg30
TD 65536
SOLVENT CDCl3
NS 512
DS 4
SWH 24038.461 Hz
FIDRES 0.366798 Hz
AQ 1.3631488 sec
RG 201.48
DW 20.800 usec
DE 6.50 usec
TE 300.0 K
D1 2.00000000 sec
D11 0.03000000 sec
TD0 1
======= CHANNEL f1 =======
SFO1 100.6304993 MHz
NUC1 13C
P1 8.20 usec
PLW1 70.59999847 W
======= CHANNEL f2 =======
SFO2 400.1621006 MHz
NUC2 1H
CPDPRG[2 waltz16
PCPD2 90.00 usec
PLW2 14.00000000 W
PLW12 0.26361999 W
PLW13 0.21353000 W
F2 – Processing parameters
SI 32768
SF 100.6204380 MHz
WDW EM
SSB 0
LB 1.00 Hz
GB 0
PC 1.40
NH
O
3
200
190
180
170
160
150
140
130
120
110
100
90
80
70
60
50
40
30
20
ppm

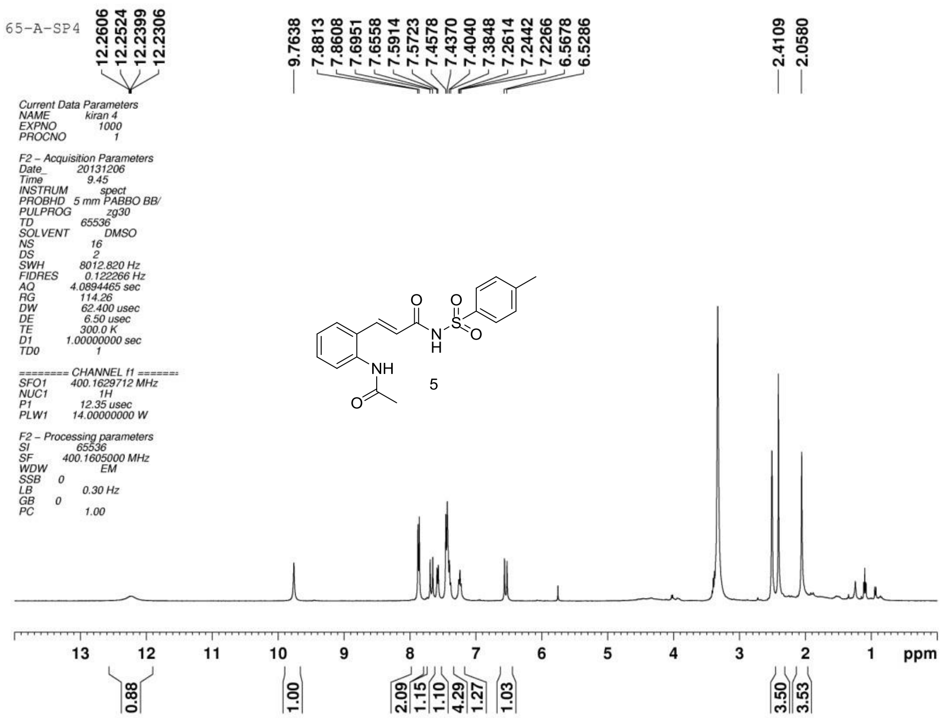
65-A-SP4
12.2606
12.2524
12.2399
12.2306
9.7638
7.8813
7.8608
7.6951
7.6558
7.5914
7.5723
7.4578
7.4370
7.4040
7.3848
7.2614
7.2442
7.2266
6.5678
6.5286
2.4109
2.0580
Current Data Parameters
NAME kiran 4
EXPNO 1000
PROCNO 1
F2 – Acquisition Parameters
Date_ 20131206
Time 9.45
INSTRUM spect
PROBHD 5 mm PABBO BB/
PULPROG zg30
TD 65536
SOLVENT DMSO
NS 16
DS 2
SWH 8012.820 Hz
FIDRES 0.122266 Hz
AQ 4.0894465 sec
RG 114.26
DW 62.400 usec
DE 6.50 usec
TE 300.0 K
D1 1.00000000 sec
TD0 1
======== CHANNEL f1 ========
SFO1 400.1629712 MHz
NUC1 1H
P1 12.35 usec
PLW1 14.00000000 W
F2 – Processing parameters
SI 65536
SF 400.1605000 MHz
WDW EM
SSB 0
LB 0.30 Hz
GB 0
PC 1.00
O
O
N
S
O
H
NH
O
5
13
12
11
10
9
8
7
6
5
4
3
2
1
ppm
0.88
1.00
2.09
1.15
1.10
4.29
1.27
1.03
3.50
3.53

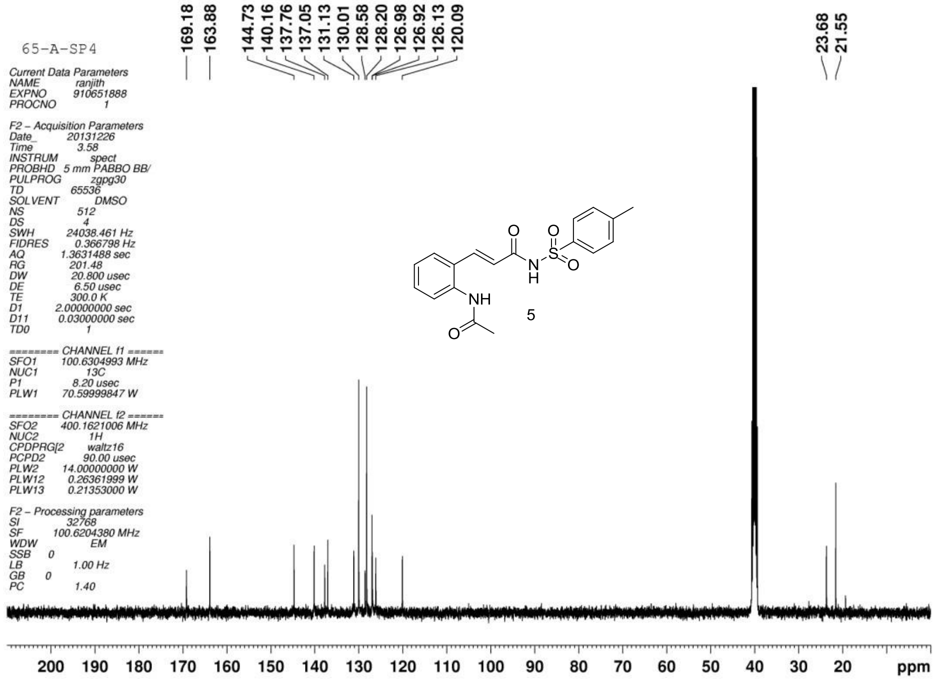
169.18
163.88
144.73
140.16
137.76
137.05
131.13
130.01
128.58
128.20
126.98
126.92
126.13
120.09
23.68
21.55
65-A-SP4
Current Data Parameters
NAME ranjith
EXPNO 910651888
PROCNO 1
F2 – Acquisition Parameters
Date_ 20131226
Time 3.58
INSTRUM spect
PROBHD 5 mm PABBO BB/
PULPROG zgpg30
TD 65536
SOLVENT DMSO
NS 512
DS 4
SWH 24038.461 Hz
FIDRES 0.366798 Hz
AQ 1.3631488 sec
RG 201.48
DW 20.800 usec
DE 6.50 usec
TE 300.0 K
D1 2.00000000 sec
D11 0.03000000 sec
TD0 1
======== CHANNEL f1 ========
SFO1 100.6304993 MHz
NUC1 13C
P1 8.20 usec
PLW1 70.59999847 W
======== CHANNEL f2 ========
SFO2 400.1621006 MHz
NUC2 1H
CPDPRG[2 waltz16
PCPD2 90.00 usec
PLW2 14.00000000 W
PLW12 0.26361999 W
PLW13 0.21353000 W
F2 – Processing parameters
SI 32768
SF 100.6204380 MHz
WDW EM
SSB 0
LB 1.00 Hz
GB 0
PC 1.40
O
O
N
S
O
H
NH
O
5
200
190
180
170
160
150
140
130
120
110
100
90
80
70
60
50
40
30
20
ppm

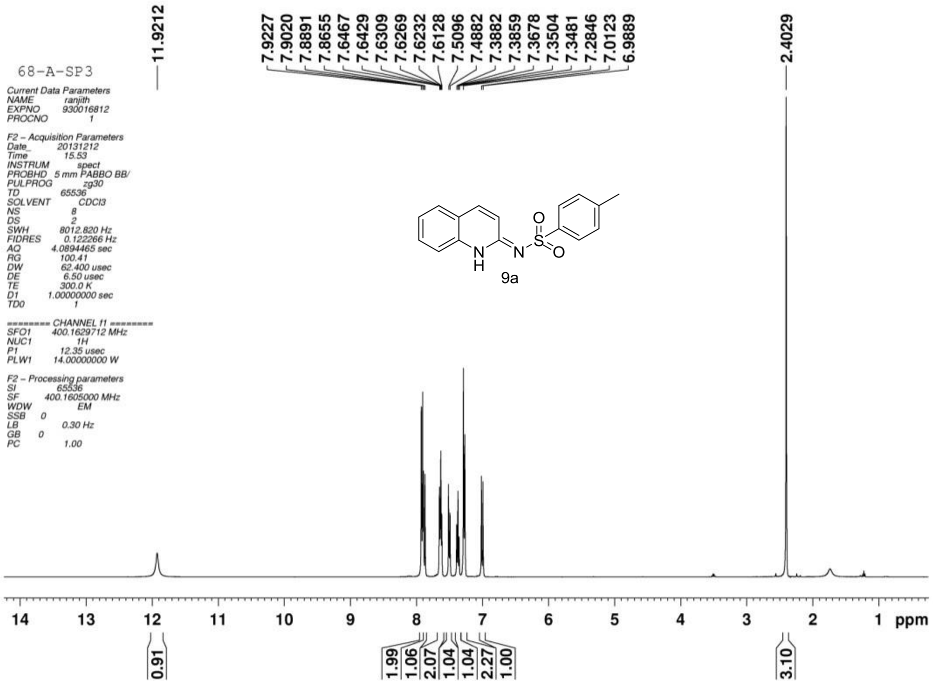
68-A-SP3
Current Data Parameters
NAME ranjith
EXPNO 930016812
PROCNO 1
F2 – Acquisition Parameters
Date_ 20131212
Time 15.53
INSTRUM spect
PROBHD 5 mm PABBO BB/
PULPROG zg30
TD 65536
SOLVENT CDCl3
NS 8
DS 2
SWH 8012.820 Hz
FIDRES 0.122266 Hz
AQ 4.0894465 sec
RG 100.41
DW 62.400 usec
DE 6.50 usec
TE 300.0 K
D1 1.00000000 sec
TD0 1
======== CHANNEL f1 ========
SFO1 400.1629712 MHz
NUC1 1H
P1 12.35 usec
PLW1 14.00000000 W
F2 – Processing parameters
SI 65536
SF 400.1605000 MHz
WDW EM
SSB 0
LB 0.30 Hz
GB 0
PC 1.00
11.9212
7.9227
7.9020
7.8891
7.8655
7.6467
7.6429
7.6309
7.6269
7.6232
7.6128
7.5096
7.4882
7.3882
7.3859
7.3678
7.3504
7.3481
7.2846
7.0123
6.9889
2.4029
9a
14 13 12 11 10 9 8 7 6 5 4 3 2 1 ppm
0.91
1.99
1.06
2.07
1.04
1.04
2.27
1.00
3.10

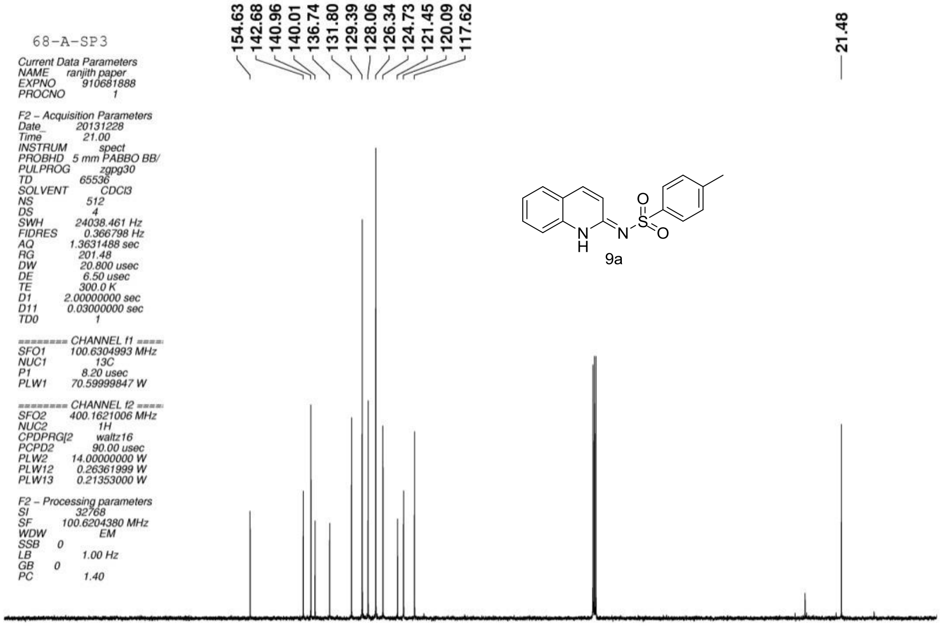
68-A-SP3
Current Data Parameters
NAME ranjith paper
EXPNO 910681888
PROCNO 1
F2 – Acquisition Parameters
Date_ 20131228
Time 21.00
INSTRUM spect
PROBHD 5 mm PABBO BB/
PULPROG zgpg30
TD 65536
SOLVENT CDCl3
NS 512
DS 4
SWH 24038.461 Hz
FIDRES 0.366798 Hz
AQ 1.3631488 sec
RG 201.48
DW 20.800 usec
DE 6.50 usec
TE 300.0 K
D1 2.00000000 sec
D11 0.03000000 sec
TD0 1
======== CHANNEL f1 ====
SFO1 100.6304993 MHz
NUC1 13C
P1 8.20 usec
PLW1 70.59999847 W
======== CHANNEL f2 ====
SFO2 400.1621006 MHz
NUC2 1H
CPDPRG[2 waltz16
PCPD2 90.00 usec
PLW2 14.00000000 W
PLW12 0.26361999 W
PLW13 0.21353000 W
F2 – Processing parameters
SI 32768
SF 100.6204380 MHz
WDW EM
SSB 0
LB 1.00 Hz
GB 0
PC 1.40
154.63
142.68
140.96
140.01
136.74
131.80
129.39
128.06
126.34
124.73
121.45
120.09
117.62
21.48
9a
200 190 180 170 160 150 140 130 120 110 100 90 80 70 60 50 40 30 20 ppm

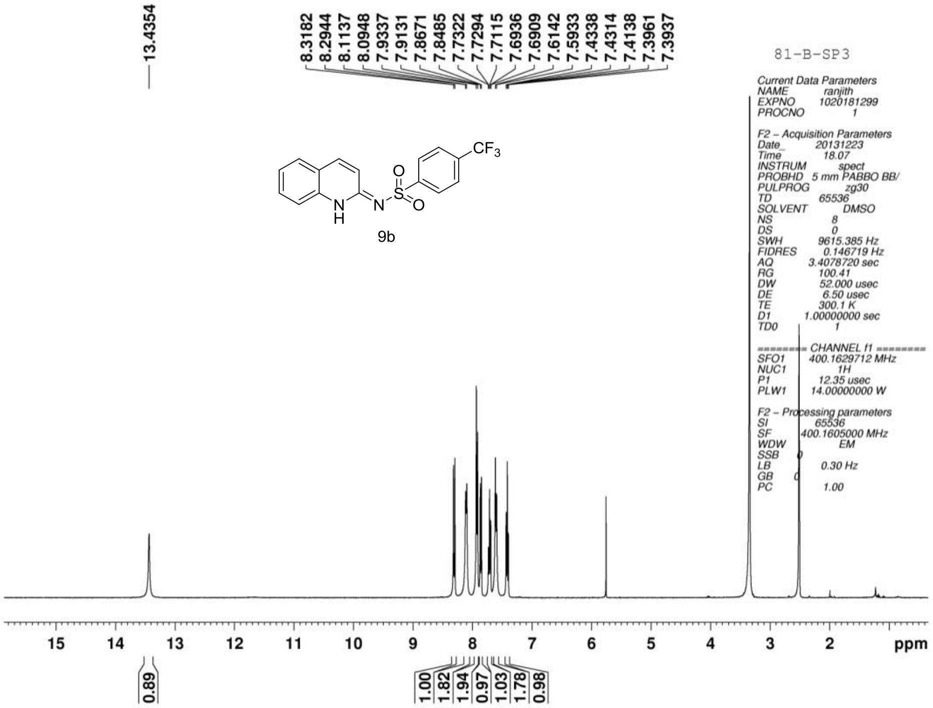
13.4354
8.3182 8.2944 8.1137 8.0948 7.9337 7.9131 7.8671 7.8485 7.7322 7.7294 7.7115 7.6936 7.6909 7.6142 7.5933 7.4338 7.4314 7.4138 7.3961 7.3937
81-B-SP3
Current Data Parameters
NAME ranjith
EXPNO 1020181299
PROCNO 1
F2 - Acquisition Parameters
Date_ 20131223
Time 18.07
INSTRUM spect
PROBHD 5 mm PABBO BB/
PULPROG zg30
TD 65536
SOLVENT DMSO
NS 8
DS 0
SWH 9615.385 Hz
FIDRES 0.146719 Hz
AQ 3.4078720 sec
RG 100.41
DW 52.000 usec
DE 6.50 usec
TE 300.1 K
D1 1.00000000 sec
TD0 1
======== CHANNEL f1 ========
SFO1 400.1629712 MHz
NUC1 1H
P1 12.35 usec
PLW1 14.00000000 W
F2 - Processing parameters
SI 65536
SF 400.1605000 MHz
WDW EM
SSB 0
LB 0.30 Hz
GB 0
PC 1.00
CF3
O
N
S
O
N
H
9b
15 14 13 12 11 10 9 8 7 6 5 4 3 2 ppm
0.89
1.00 1.82 1.94 0.97 1.03 1.78 0.98

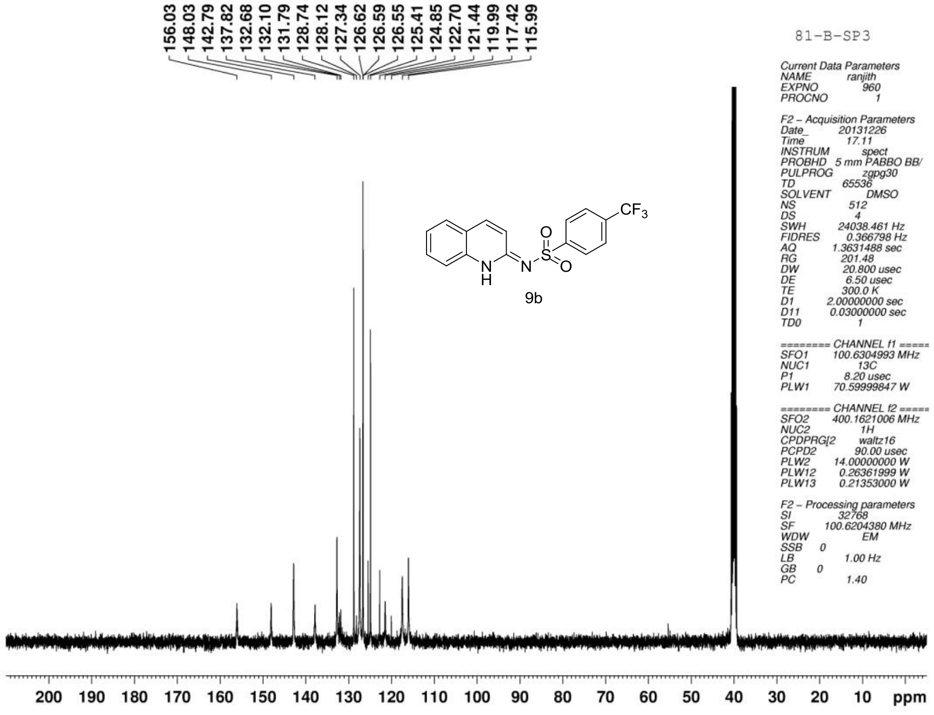
156.03 148.03 142.79 137.82 132.68 132.10 131.79 128.74 128.12 127.34 126.62 126.59 126.55 125.41 124.85 122.70 121.44 119.99 117.42 115.99
81-B-SP3
Current Data Parameters
NAME ranjith
EXPNO 960
PROCNO 1
F2 - Acquisition Parameters
Date_ 20131226
Time 17.11
INSTRUM spect
PROBHD 5 mm PABBO BB/
PULPROG zgpg30
TD 65536
SOLVENT DMSO
NS 512
DS 4
SWH 24038.461 Hz
FIDRES 0.366798 Hz
AQ 1.3631488 sec
RG 201.48
DW 20.800 usec
DE 6.50 usec
TE 300.0 K
D1 2.00000000 sec
D11 0.03000000 sec
TD0 1
======== CHANNEL f1 =====
SFO1 100.6304993 MHz
NUC1 13C
P1 8.20 usec
PLW1 70.59999847 W
======== CHANNEL f2 =====
SFO2 400.1621006 MHz
NUC2 1H
CPDPRG[2 waltz16
PCPD2 90.00 usec
PLW2 14.00000000 W
PLW12 0.26361999 W
PLW13 0.21353000 W
F2 - Processing parameters
SI 32768
SF 100.6204380 MHz
WDW EM
SSB 0
LB 1.00 Hz
GB 0
PC 1.40
CF3
O
N
S
O
N
H
9b
200 190 180 170 160 150 140 130 120 110 100 90 80 70 60 50 40 30 20 10 ppm

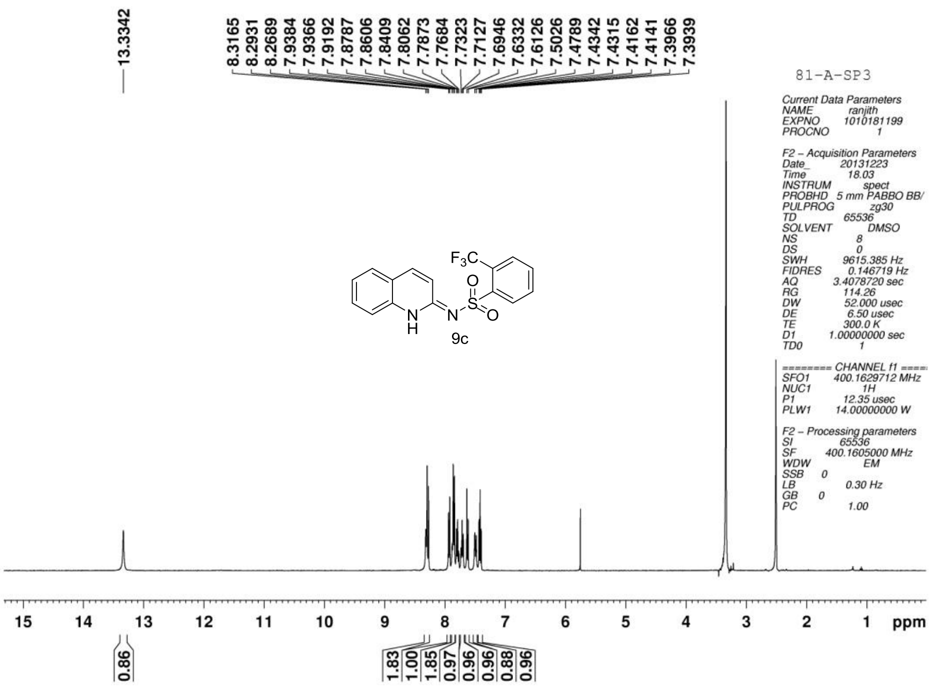
13.3342
8.3165 8.2931 8.2689 7.9384 7.9366 7.9192 7.8787 7.8606 7.8409 7.8062 7.7873 7.7684 7.7323 7.7127 7.6946 7.6332 7.6126 7.5026 7.4789 7.4342 7.4315 7.4162 7.4141 7.3966 7.3939
81-A-SP3
Current Data Parameters
NAME ranjith
EXPNO 1010181199
PROCNO 1
F2 – Acquisition Parameters
Date_ 20131223
Time 18.03
INSTRUM spect
PROBHD 5 mm PABBO BB/
PULPROG zg30
TD 65536
SOLVENT DMSO
NS 8
DS 0
SWH 9615.385 Hz
FIDRES 0.146719 Hz
AQ 3.4078720 sec
RG 114.26
DW 52.000 usec
DE 6.50 usec
TE 300.0 K
D1 1.00000000 sec
TD0 1
======== CHANNEL f1 ========
SFO1 400.1629712 MHz
NUC1 1H
P1 12.35 usec
PLW1 14.00000000 W
F2 – Processing parameters
SI 65536
SF 400.1605000 MHz
WDW EM
SSB 0
LB 0.30 Hz
GB 0
PC 1.00
9c
0.86
1.83 1.00 1.85 0.97 0.96 0.96 0.88 0.96
15 14 13 12 11 10 9 8 7 6 5 4 3 2 1 ppm

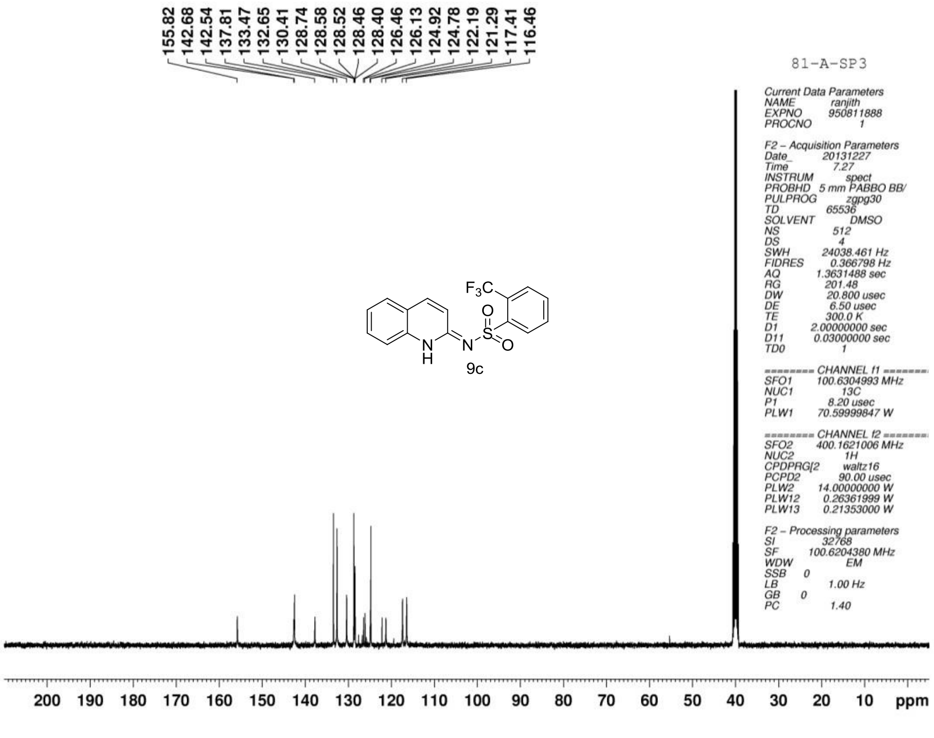
155.82 142.68 142.54 137.81 133.47 132.65 130.41 128.74 128.58 128.52 128.46 128.40 126.46 126.13 124.92 124.78 122.19 121.29 117.41 116.46
81-A-SP3
Current Data Parameters
NAME ranjith
EXPNO 950811888
PROCNO 1
F2 – Acquisition Parameters
Date_ 20131227
Time 7.27
INSTRUM spect
PROBHD 5 mm PABBO BB/
PULPROG zgpg30
TD 65536
SOLVENT DMSO
NS 512
DS 4
SWH 24038.461 Hz
FIDRES 0.366798 Hz
AQ 1.3631488 sec
RG 201.48
DW 20.800 usec
DE 6.50 usec
TE 300.0 K
D1 2.00000000 sec
D11 0.03000000 sec
TD0 1
======== CHANNEL f1 ========
SFO1 100.6304993 MHz
NUC1 13C
P1 8.20 usec
PLW1 70.59999847 W
======== CHANNEL f2 ========
SFO2 400.1621006 MHz
NUC2 1H
CPDPRG[2 waltz16
PCPD2 90.00 usec
PLW2 14.00000000 W
PLW12 0.26361999 W
PLW13 0.21353000 W
F2 – Processing parameters
SI 32768
SF 100.6204380 MHz
WDW EM
SSB 0
LB 1.00 Hz
GB 0
PC 1.40
9c
200 190 180 170 160 150 140 130 120 110 100 90 80 70 60 50 40 30 20 10 ppm

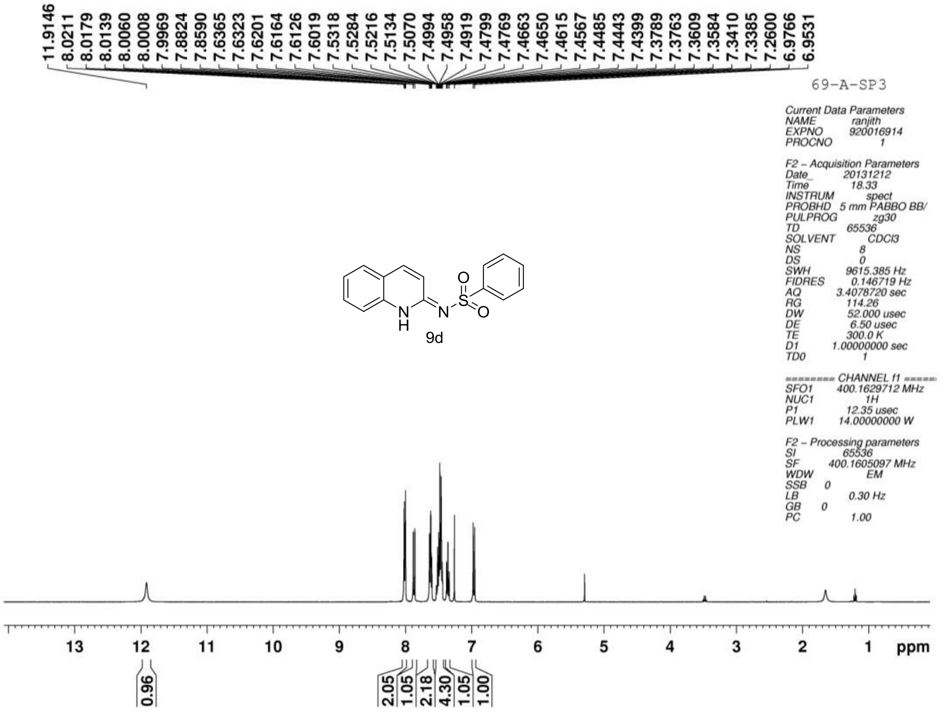

11.9146
8.0211
8.0179
8.0139
8.0060
8.0008
7.9969
7.8824
7.8590
7.6365
7.6323
7.6201
7.6164
7.6126
7.6019
7.5318
7.5284
7.5216
7.5134
7.5070
7.4994
7.4958
7.4919
7.4799
7.4769
7.4663
7.4650
7.4615
7.4567
7.4485
7.4443
7.4399
7.3789
7.3763
7.3609
7.3584
7.3410
7.3385
7.2600
6.9766
6.9531
69-A-SP3
Current Data Parameters
NAME ranjith
EXPNO 920016914
PROCNO 1
F2 - Acquisition Parameters
Date_ 20131212
Time 18.33
INSTRUM spect
PROBHD 5 mm PABBO BB/
PULPROG zg30
TD 65536
SOLVENT CDCl3
NS 8
DS 0
SWH 9615.385 Hz
FIDRES 0.146719 Hz
AQ 3.4078720 sec
RG 114.26
DW 52.000 usec
DE 6.50 usec
TE 300.0 K
D1 1.00000000 sec
TD0 1
======== CHANNEL f1 ========
SFO1 400.1629712 MHz
NUC1 1H
P1 12.35 usec
PLW1 14.00000000 W
F2 - Processing parameters
SI 65536
SF 400.1605097 MHz
WDW EM
SSB 0
LB 0.30 Hz
GB 0
PC 1.00
N
H
S
O
O
9d
13 12 11 10 9 8 7 6 5 4 3 2 1 ppm
0.96
2.05
1.05
2.18
4.30
1.05
1.00

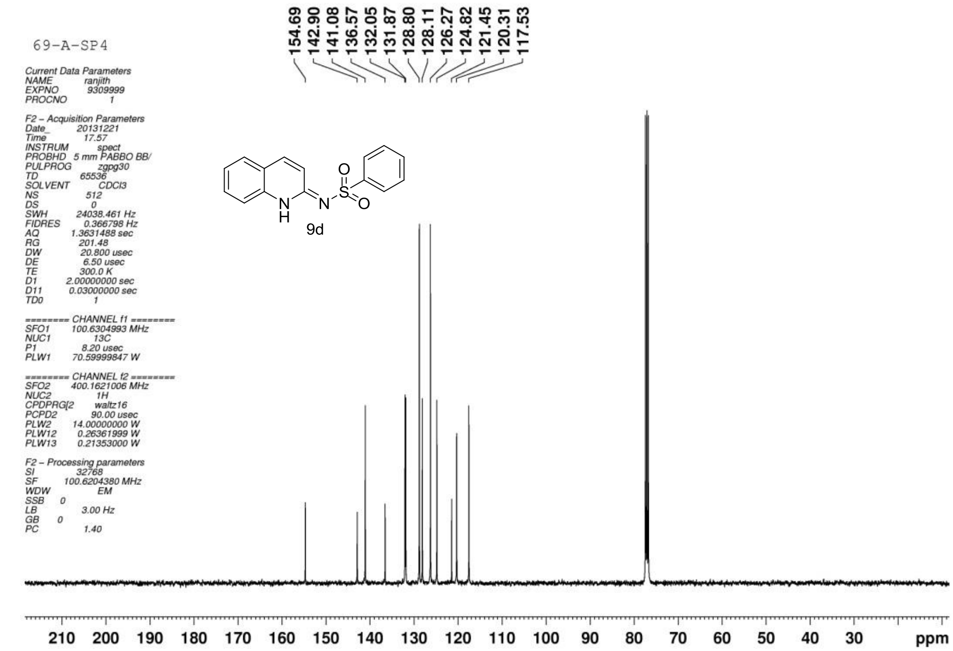

154.69
142.90
141.08
136.57
132.05
131.87
128.80
128.11
126.27
124.82
121.45
120.31
117.53
69-A-SP4
Current Data Parameters
NAME ranjith
EXPNO 9309999
PROCNO 1
F2 - Acquisition Parameters
Date_ 20131221
Time 17.57
INSTRUM spect
PROBHD 5 mm PABBO BB/
PULPROG zgpg30
TD 65536
SOLVENT CDCl3
NS 512
DS 0
SWH 24038.461 Hz
FIDRES 0.366798 Hz
AQ 1.3631488 sec
RG 201.48
DW 20.800 usec
DE 6.50 usec
TE 300.0 K
D1 2.00000000 sec
D11 0.03000000 sec
TD0 1
======== CHANNEL f1 ========
SFO1 100.6304993 MHz
NUC1 13C
P1 8.20 usec
PLW1 70.59999847 W
======== CHANNEL f2 ========
SFO2 400.1621006 MHz
NUC2 1H
CPDPRG[2 waltz16
PCPD2 90.00 usec
PLW2 14.00000000 W
PLW12 0.26361999 W
PLW13 0.21353000 W
F2 - Processing parameters
SI 32768
SF 100.6204380 MHz
WDW EM
SSB 0
LB 3.00 Hz
GB 0
PC 1.40
N
H
S
O
O
9d
210 200 190 180 170 160 150 140 130 120 110 100 90 80 70 60 50 40 30 ppm

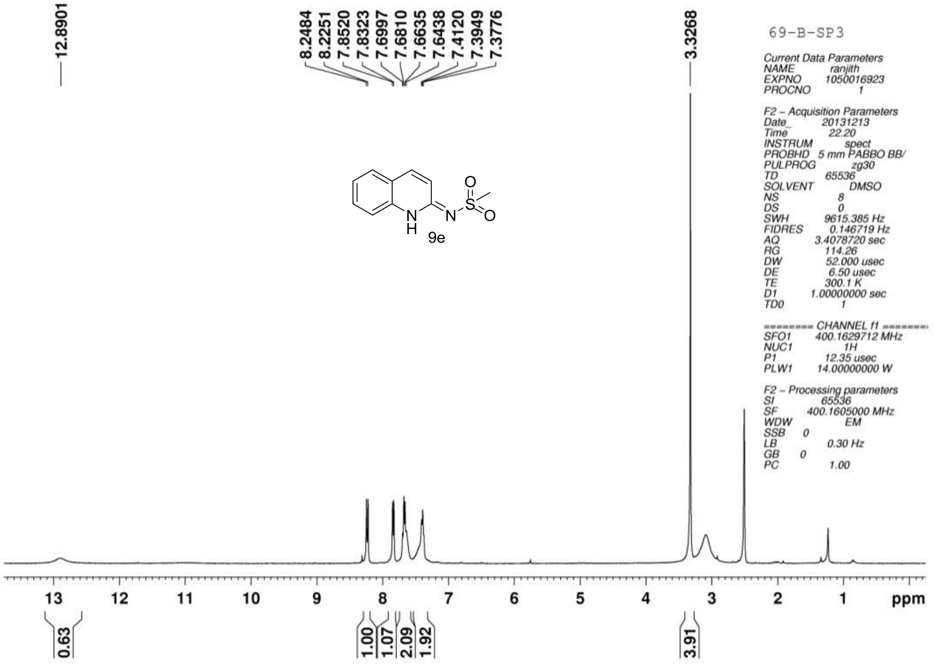
12.8901
8.2484
8.2251
7.8520
7.8323
7.6997
7.6810
7.6635
7.6438
7.4120
7.3949
7.3776
3.3268
69-B-SP3
Current Data Parameters
NAME ranjith
EXPNO 1050016923
PROCNO 1
F2 – Acquisition Parameters
Date_ 20131213
Time 22.20
INSTRUM spect
PROBHD 5 mm PABBO BB/
PULPROG zg30
TD 65536
SOLVENT DMSO
NS 8
DS 0
SWH 9615.385 Hz
FIDRES 0.146719 Hz
AQ 3.4078720 sec
RG 114.26
DW 52.000 usec
DE 6.50 usec
TE 300.1 K
D1 1.00000000 sec
TD0 1
======== CHANNEL f1 ========
SFO1 400.1629712 MHz
NUC1 1H
P1 12.35 usec
PLW1 14.00000000 W
F2 – Processing parameters
SI 65536
SF 400.1605000 MHz
WDW EM
SSB 0
LB 0.30 Hz
GB 0
PC 1.00
N
H
S
O
O
9e
13 12 11 10 9 8 7 6 5 4 3 2 1 ppm
0.63
1.00
1.07
2.09
1.92
3.91

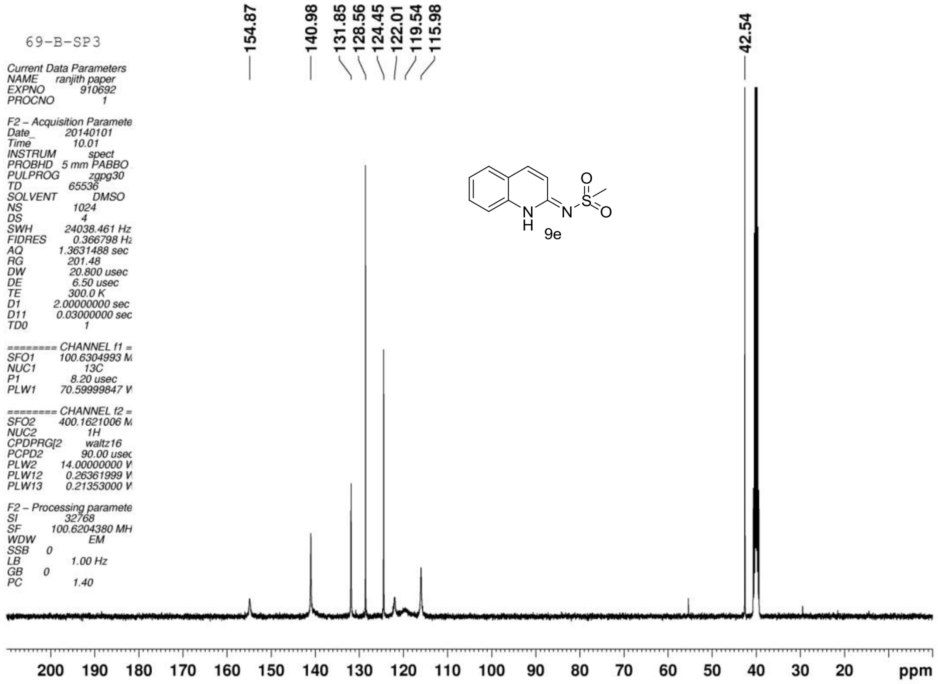
154.87
140.98
131.85
128.56
124.45
122.01
119.54
115.98
42.54
69-B-SP3
Current Data Parameters
NAME ranjith paper
EXPNO 910692
PROCNO 1
F2 – Acquisition Paramete
Date_ 20140101
Time 10.01
INSTRUM spect
PROBHD 5 mm PABBO
PULPROG zgpg30
TD 65536
SOLVENT DMSO
NS 1024
DS 4
SWH 24038.461 Hz
FIDRES 0.366798 Hz
AQ 1.3631488 sec
RG 201.48
DW 20.800 usec
DE 6.50 usec
TE 300.0 K
D1 2.00000000 sec
D11 0.03000000 sec
TD0 1
======== CHANNEL f1 =
SFO1 100.6304993 M
NUC1 13C
P1 8.20 usec
PLW1 70.59999847 W
======== CHANNEL f2 =
SFO2 400.1621006 M
NUC2 1H
CPDPRG[2 waltz16
PCPD2 90.00 usec
PLW2 14.00000000 W
PLW12 0.26361999 W
PLW13 0.21353000 W
F2 – Processing paramete
SI 32768
SF 100.6204380 MH
WDW EM
SSB 0
LB 1.00 Hz
GB 0
PC 1.40
N
H
S
O
O
9e
200 190 180 170 160 150 140 130 120 110 100 90 80 70 60 50 40 30 20 ppm

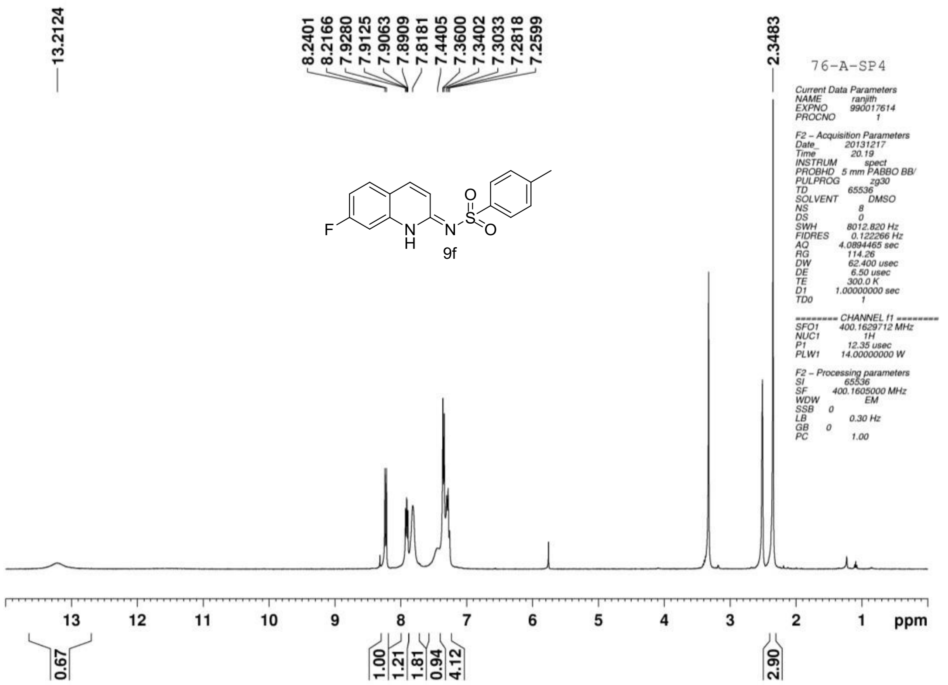
13.2124
8.2401
8.2166
7.9280
7.9125
7.9063
7.8909
7.8181
7.4405
7.3600
7.3402
7.3033
7.2818
7.2599
2.3483
76-A-SP4
Current Data Parameters
NAME ranjith
EXPNO 990017614
PROCNO 1
F2 - Acquisition Parameters
Date_ 20131217
Time 20.19
INSTRUM spect
PROBHD 5 mm PABBO BB/
PULPROG zg30
TD 65536
SOLVENT DMSO
NS 8
DS 0
SWH 8012.820 Hz
FIDRES 0.122266 Hz
AQ 4.0894465 sec
RG 114.26
DW 62.400 usec
DE 6.50 usec
TE 300.0 K
D1 1.00000000 sec
TD0 1
======= CHANNEL f1 =======
SFO1 400.1629712 MHz
NUC1 1H
P1 12.35 usec
PLW1 14.00000000 W
F2 - Processing parameters
SI 65536
SF 400.1605000 MHz
WDW EM
SSB 0
LB 0.30 Hz
GB 0
PC 1.00
F
N
H
N
S
O
O
9f
13 12 11 10 9 8 7 6 5 4 3 2 1 ppm
0.67
1.00
1.21
1.81
0.94
4.12
2.90

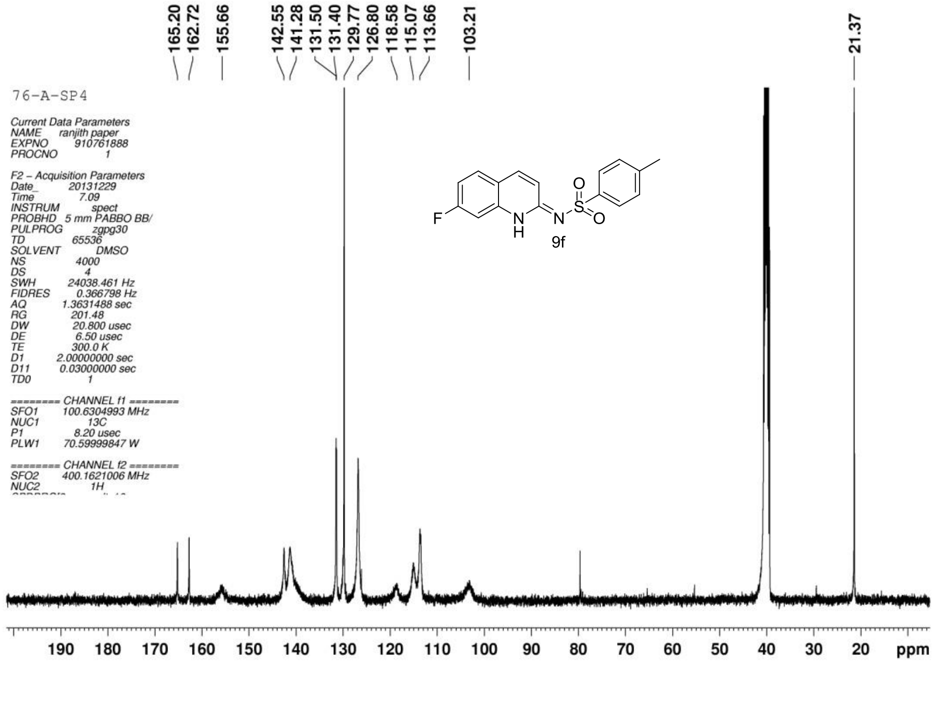
165.20
162.72
155.66
142.55
141.28
131.50
131.40
129.77
126.80
118.58
115.07
113.66
103.21
21.37
76-A-SP4
Current Data Parameters
NAME ranjith paper
EXPNO 910761888
PROCNO 1
F2 - Acquisition Parameters
Date_ 20131229
Time 7.09
INSTRUM spect
PROBHD 5 mm PABBO BB/
PULPROG zgpg30
TD 65536
SOLVENT DMSO
NS 4000
DS 4
SWH 24038.461 Hz
FIDRES 0.366798 Hz
AQ 1.3631488 sec
RG 201.48
DW 20.800 usec
DE 6.50 usec
TE 300.0 K
D1 2.00000000 sec
D11 0.03000000 sec
TD0 1
======= CHANNEL f1 =======
SFO1 100.6304993 MHz
NUC1 13C
P1 8.20 usec
PLW1 70.59999847 W
======= CHANNEL f2 =======
SFO2 400.1621006 MHz
NUC2 1H
F
N
H
N
S
O
O
9f
190 180 170 160 150 140 130 120 110 100 90 80 70 60 50 40 30 20 ppm

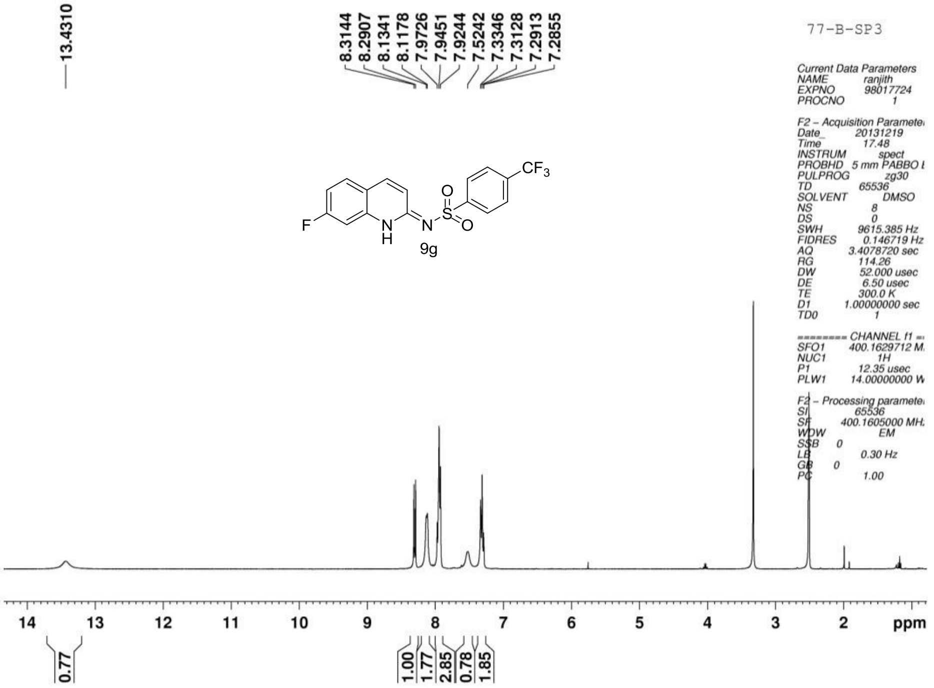
13.4310
8.3144
8.2907
8.1341
8.1178
7.9726
7.9451
7.9244
7.5242
7.3346
7.3128
7.2913
7.2855
77-B-SP3
Current Data Parameters
NAME ranjith
EXPNO 98017724
PROCNO 1
F2 - Acquisition Paramete
Date_ 20131219
Time 17.48
INSTRUM spect
PULPROG zg30
TD 65536
SOLVENT DMSO
NS 8
DS 0
SWH 9615.385 Hz
FIDRES 0.146719 Hz
AQ 3.4078720 sec
RG 114.26
DW 52.000 usec
DE 6.50 usec
TE 300.0 K
D1 1.00000000 sec
TD0 1
CHANNEL f1
SFO1 400.1629712 M
NUC1 1H
P1 12.35 usec
PLW1 14.00000000 W
F2 - Processing paramete
SI 65536
SF 400.1605000 MH
WDW EM
SSB 0
LB 0.30 Hz
GB 0
PC 1.00
CF3
F
N
H
N
S
O
O
9g
14 13 12 11 10 9 8 7 6 5 4 3 2 ppm
0.77
1.00
1.77
2.85
0.78
1.85

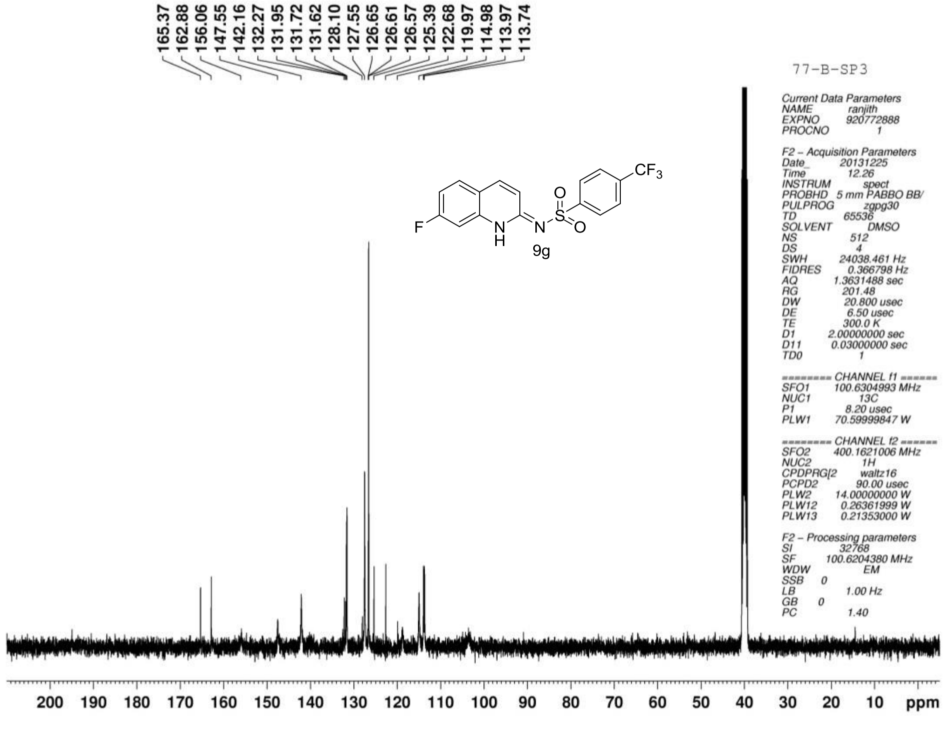
165.37
162.88
156.06
147.55
142.16
132.27
131.95
131.72
131.62
128.10
127.55
126.65
126.61
126.57
125.39
122.68
119.97
114.98
113.97
113.74
77-B-SP3
Current Data Parameters
NAME ranjith
EXPNO 920772888
PROCNO 1
F2 - Acquisition Parameters
Date_ 20131225
Time 12.26
INSTRUM spect
PROBHD 5 mm PABBO BB/
PULPROG zgpg30
TD 65536
SOLVENT DMSO
NS 512
DS 4
SWH 24038.461 Hz
FIDRES 0.366798 Hz
AQ 1.3631488 sec
RG 201.48
DW 20.800 usec
DE 6.50 usec
TE 300.0 K
D1 2.00000000 sec
D11 0.03000000 sec
TD0 1
CHANNEL f1
SFO1 100.6304993 MHz
NUC1 13C
P1 8.20 usec
PLW1 70.59999847 W
CHANNEL f2
SFO2 400.1621006 MHz
NUC2 1H
CPDPRG[2 waltz16
PCPD2 90.00 usec
PLW2 14.00000000 W
PLW12 0.26361999 W
PLW13 0.21353000 W
F2 - Processing parameters
SI 32768
SF 100.6204380 MHz
WDW EM
SSB 0
LB 1.00 Hz
GB 0
PC 1.40
CF3
F
N
H
N
S
O
O
9g
200 190 180 170 160 150 140 130 120 110 100 90 80 70 60 50 40 30 20 10 ppm

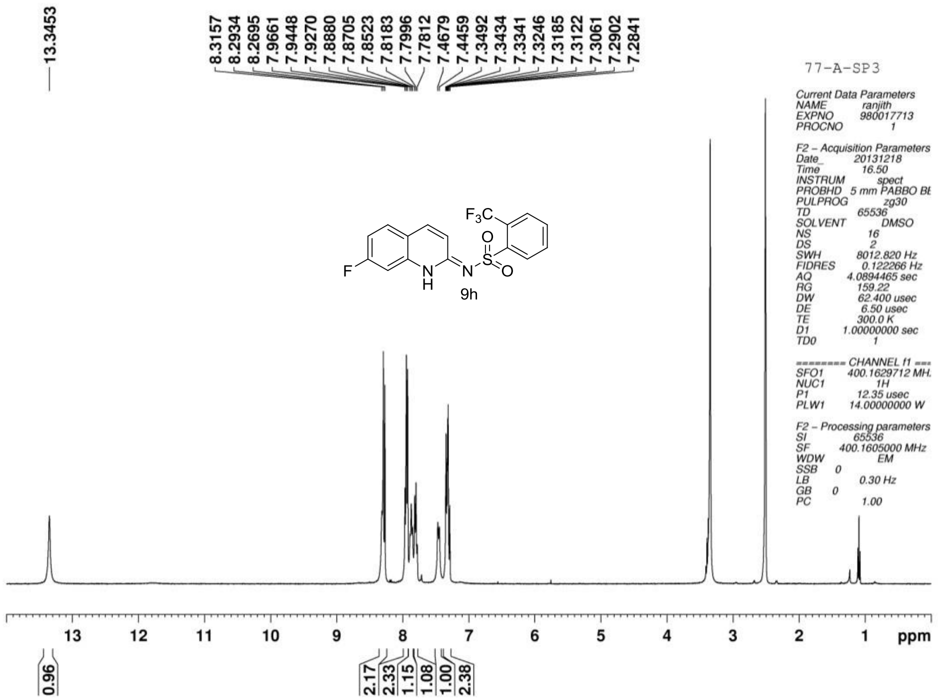
13.3453
8.3157
8.2934
8.2695
7.9661
7.9448
7.9270
7.8880
7.8705
7.8523
7.8183
7.7996
7.7812
7.4679
7.4459
7.3492
7.3434
7.3341
7.3246
7.3185
7.3122
7.3061
7.2902
7.2841
77-A-SP3
Current Data Parameters
NAME ranjith
EXPNO 980017713
PROCNO 1
F2 - Acquisition Parameters
Date_ 20131218
Time 16.50
INSTRUM spect
PROBHD 5 mm PABBO BB
PULPROG zg30
TD 65536
SOLVENT DMSO
NS 16
DS 2
SWH 8012.820 Hz
FIDRES 0.122266 Hz
AQ 4.0894465 sec
RG 159.22
DW 62.400 usec
DE 6.50 usec
TE 300.0 K
D1 1.00000000 sec
TD0 1
======= CHANNEL f1 ===
SFO1 400.1629712 MH
NUC1 1H
P1 12.35 usec
PLW1 14.00000000 W
F2 - Processing parameters
SI 65536
SF 400.1605000 MHz
WDW EM
SSB 0
LB 0.30 Hz
GB 0
PC 1.00
F
H
N
S
O
F_3C
9h
13 12 11 10 9 8 7 6 5 4 3 2 1 ppm
0.96
2.17
2.33
1.15
1.08
1.00
2.38

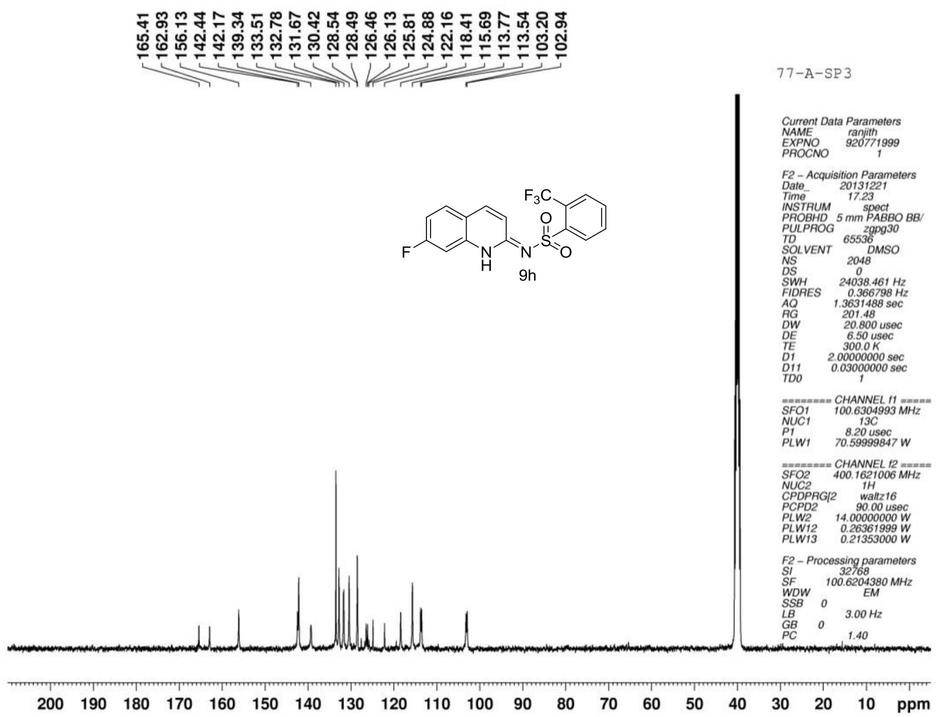
165.41
162.93
156.13
142.44
142.17
139.34
133.51
132.78
131.67
130.42
128.54
128.49
126.46
126.13
125.81
124.88
122.16
118.41
115.69
113.77
113.54
103.20
102.94
77-A-SP3
Current Data Parameters
NAME ranjith
EXPNO 920771999
PROCNO 1
F2 - Acquisition Parameters
Date_ 20131221
Time 17.23
INSTRUM spect
PROBHD 5 mm PABBO BB/
PULPROG zgpg30
TD 65536
SOLVENT DMSO
NS 2048
DS 0
SWH 24038.461 Hz
FIDRES 0.366798 Hz
AQ 1.3631488 sec
RG 201.48
DW 20.800 usec
DE 6.50 usec
TE 300.0 K
D1 2.00000000 sec
D11 0.03000000 sec
TD0 1
======= CHANNEL f1 =====
SFO1 100.6304993 MHz
NUC1 13C
P1 8.20 usec
PLW1 70.59999847 W
======= CHANNEL f2 =====
SFO2 400.1621006 MHz
NUC2 1H
CPDPRG[2 waltz16
PCPD2 90.00 usec
PLW2 14.00000000 W
PLW12 0.26361999 W
PLW13 0.21353000 W
F2 - Processing parameters
SI 32768
SF 100.6204380 MHz
WDW EM
SSB 0
LB 3.00 Hz
GB 0
PC 1.40
F
H
N
S
O
F_3C
9h
200 190 180 170 160 150 140 130 120 110 100 90 80 70 60 50 40 30 20 10 ppm

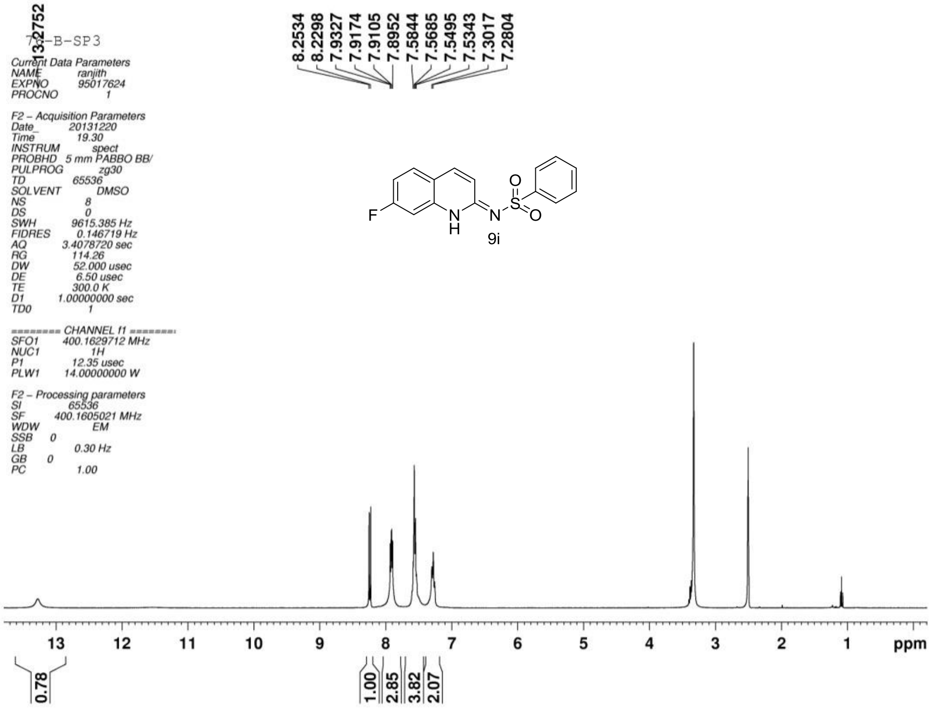
13.2752
76-B-SP3
8.2534
8.2298
7.9327
7.9174
7.9105
7.8952
7.5844
7.5685
7.5495
7.5343
7.3017
7.2804
Current Data Parameters
NAME ranjith
EXPNO 95017624
PROCNO 1
F2 – Acquisition Parameters
Date_ 20131220
Time 19.30
INSTRUM spect
PROBHD 5 mm PABBO BB/
PULPROG zg30
TD 65536
SOLVENT DMSO
NS 8
DS 0
SWH 9615.385 Hz
FIDRES 0.146719 Hz
AQ 3.4078720 sec
RG 114.26
DW 52.000 usec
DE 6.50 usec
TE 300.0 K
D1 1.00000000 sec
TD0 1
======= CHANNEL f1 =======
SFO1 400.1629712 MHz
NUC1 1H
P1 12.35 usec
PLW1 14.00000000 W
F2 – Processing parameters
SI 65536
SF 400.1605021 MHz
WDW EM
SSB 0
LB 0.30 Hz
GB 0
PC 1.00
F
N
H
O
S
O
N
9i
13
12
11
10
9
8
7
6
5
4
3
2
1
ppm
0.78
1.00
2.85
3.82
2.07

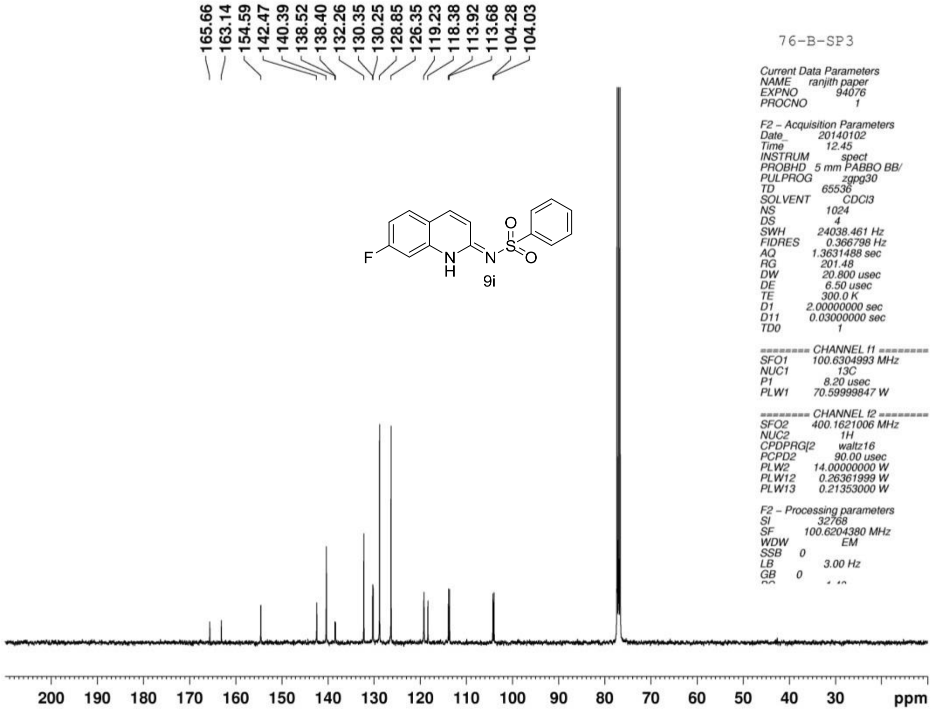
165.66
163.14
154.59
142.47
140.39
138.52
138.40
132.26
130.35
130.25
128.85
126.35
119.23
118.38
113.92
113.68
104.28
104.03
76-B-SP3
Current Data Parameters
NAME ranjith paper
EXPNO 94076
PROCNO 1
F2 – Acquisition Parameters
Date_ 20140102
Time 12.45
INSTRUM spect
PROBHD 5 mm PABBO BB/
PULPROG zgpg30
TD 65536
SOLVENT CDCl3
NS 1024
DS 4
SWH 24038.461 Hz
FIDRES 0.366798 Hz
AQ 1.3631488 sec
RG 201.48
DW 20.800 usec
DE 6.50 usec
TE 300.0 K
D1 2.00000000 sec
D11 0.03000000 sec
TD0 1
======= CHANNEL f1 =======
SFO1 100.6304993 MHz
NUC1 13C
P1 8.20 usec
PLW1 70.59999847 W
======= CHANNEL f2 =======
SFO2 400.1621006 MHz
NUC2 1H
CPDPRG[2 waltz16
PCPD2 90.00 usec
PLW2 14.00000000 W
PLW12 0.26361999 W
PLW13 0.21353000 W
F2 – Processing parameters
SI 32768
SF 100.6204380 MHz
WDW EM
SSB 0
LB 3.00 Hz
GB 0
F
N
H
O
S
O
N
9i
200
190
180
170
160
150
140
130
120
110
100
90
80
70
60
50
40
30
ppm

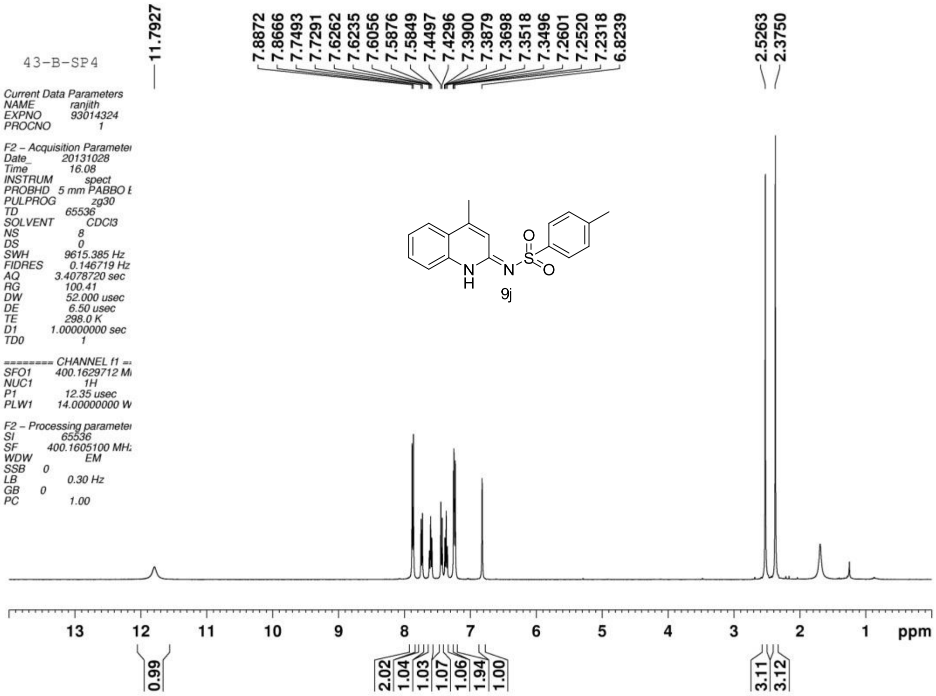
43-B-SP4
11.7927
7.8872
7.8666
7.7493
7.7291
7.6262
7.6235
7.6056
7.5876
7.5849
7.4497
7.4296
7.3900
7.3879
7.3698
7.3518
7.3496
7.2601
7.2520
7.2318
6.8239
2.5263
2.3750
Current Data Parameters
NAME ranjith
EXPNO 93014324
PROCNO 1
F2 – Acquisition Paramete
Date_ 20131028
Time 16.08
INSTRUM spect
PROBHD 5 mm PABBO
PULPROG zg30
TD 65536
SOLVENT CDCl3
NS 8
DS 0
SWH 9615.385 Hz
FIDRES 0.146719 Hz
AQ 3.4078720 sec
RG 100.41
DW 52.000 usec
DE 6.50 usec
TE 298.0 K
D1 1.00000000 sec
TD0 1
======= CHANNEL f1 ==
SFO1 400.1629712 M
NUC1 1H
P1 12.35 usec
PLW1 14.00000000 W
F2 – Processing paramete
SI 65536
SF 400.1605100 MH
WDW EM
SSB 0
LB 0.30 Hz
GB 0
PC 1.00
N
S
O
O
N
H
9j
13
12
11
10
9
8
7
6
5
4
3
2
1
ppm
0.99
2.02
1.04
1.03
1.07
1.06
1.94
1.00
3.11
3.12

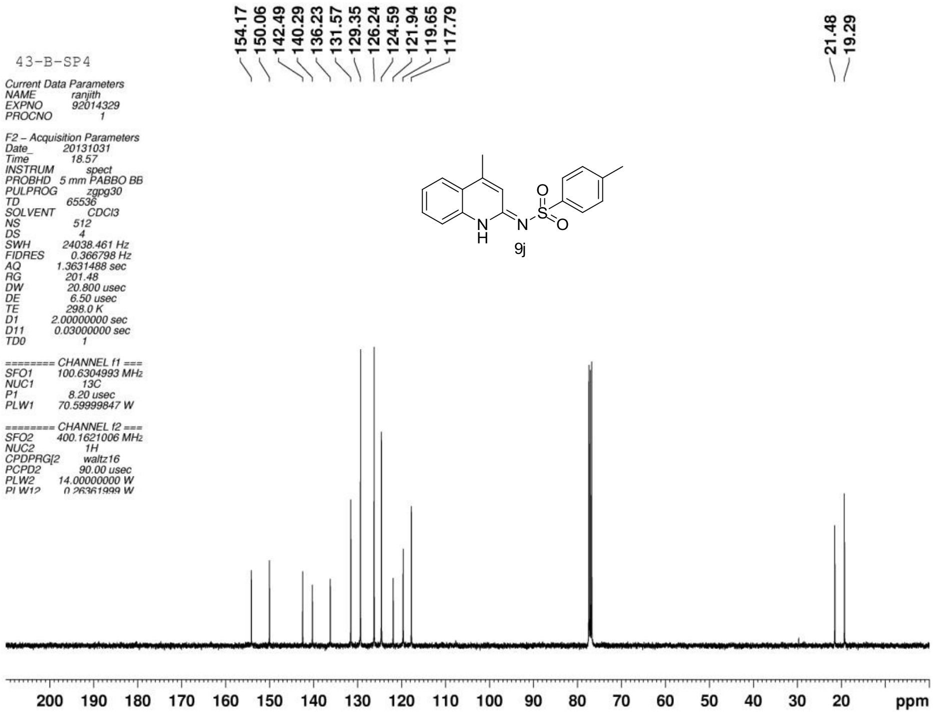
43-B-SP4
154.17
150.06
142.49
140.29
136.23
131.57
129.35
126.24
124.59
121.94
119.65
117.79
21.48
19.29
Current Data Parameters
NAME ranjith
EXPNO 92014329
PROCNO 1
F2 – Acquisition Parameters
Date_ 20131031
Time 18.57
INSTRUM spect
PROBHD 5 mm PABBO BB
PULPROG zgpg30
TD 65536
SOLVENT CDCl3
NS 512
DS 4
SWH 24038.461 Hz
FIDRES 0.366798 Hz
AQ 1.3631488 sec
RG 201.48
DW 20.800 usec
DE 6.50 usec
TE 298.0 K
D1 2.00000000 sec
D11 0.03000000 sec
TD0 1
======= CHANNEL f1 ===
SFO1 100.6304993 MHz
NUC1 13C
P1 8.20 usec
PLW1 70.59999847 W
======= CHANNEL f2 ===
SFO2 400.1621006 MHz
NUC2 1H
CPDPRG[2 waltz16
PCPD2 90.00 usec
PLW2 14.00000000 W
PLW12 0.26361999 W
N
S
O
O
N
H
9j
200
190
180
170
160
150
140
130
120
110
100
90
80
70
60
50
40
30
20
ppm

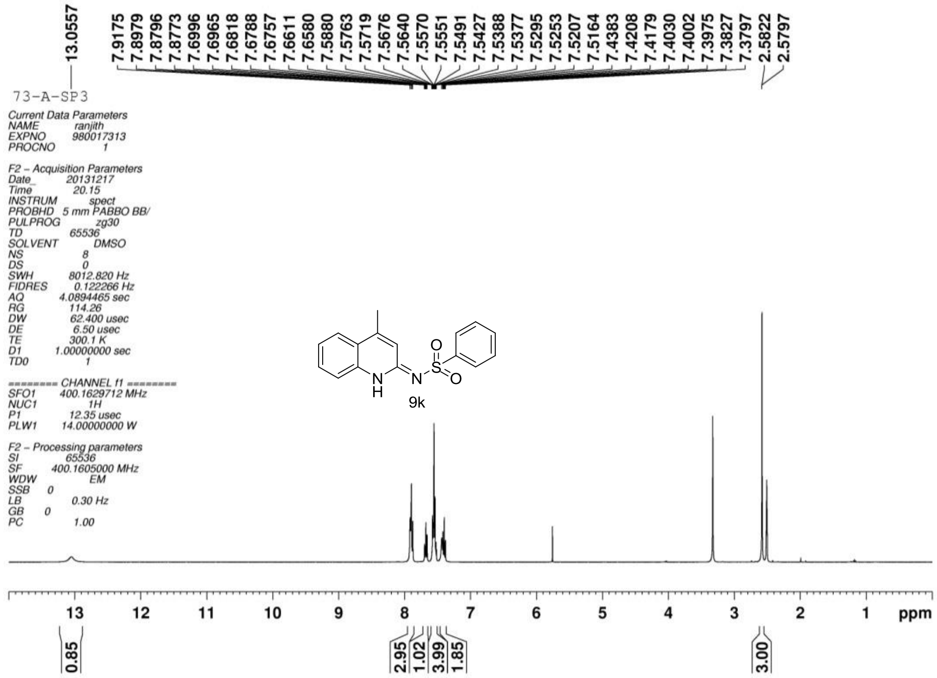
13.0557
7.9175 7.8979 7.8796 7.8773 7.6996 7.6965 7.6818 7.6788 7.6757 7.6611 7.6580 7.5880 7.5763 7.5719 7.5676 7.5640 7.5570 7.5551 7.5491 7.5427 7.5388 7.5377 7.5295 7.5253 7.5207 7.5164 7.4383 7.4208 7.4179 7.4030 7.4002 7.3975 7.3827 7.3797 2.5822 2.5797
73-A-SP3
Current Data Parameters
NAME ranjith
EXPNO 980017313
PROCNO 1
F2 – Acquisition Parameters
Date_ 20131217
Time 20.15
INSTRUM spect
PROBHD 5 mm PABBO BB/
PULPROG zg30
TD 65536
SOLVENT DMSO
NS 8
DS 0
SWH 8012.820 Hz
FIDRES 0.122266 Hz
AQ 4.0894465 sec
RG 114.26
DW 62.400 usec
DE 6.50 usec
TE 300.1 K
D1 1.00000000 sec
TD0 1
======== CHANNEL f1 ========
SFO1 400.1629712 MHz
NUC1 1H
P1 12.35 usec
PLW1 14.00000000 W
F2 – Processing parameters
SI 65536
SF 400.1605000 MHz
WDW EM
SSB 0
LB 0.30 Hz
GB 0
PC 1.00
9k
13 12 11 10 9 8 7 6 5 4 3 2 1 ppm
0.85
2.95 1.02 3.99 1.85
3.00

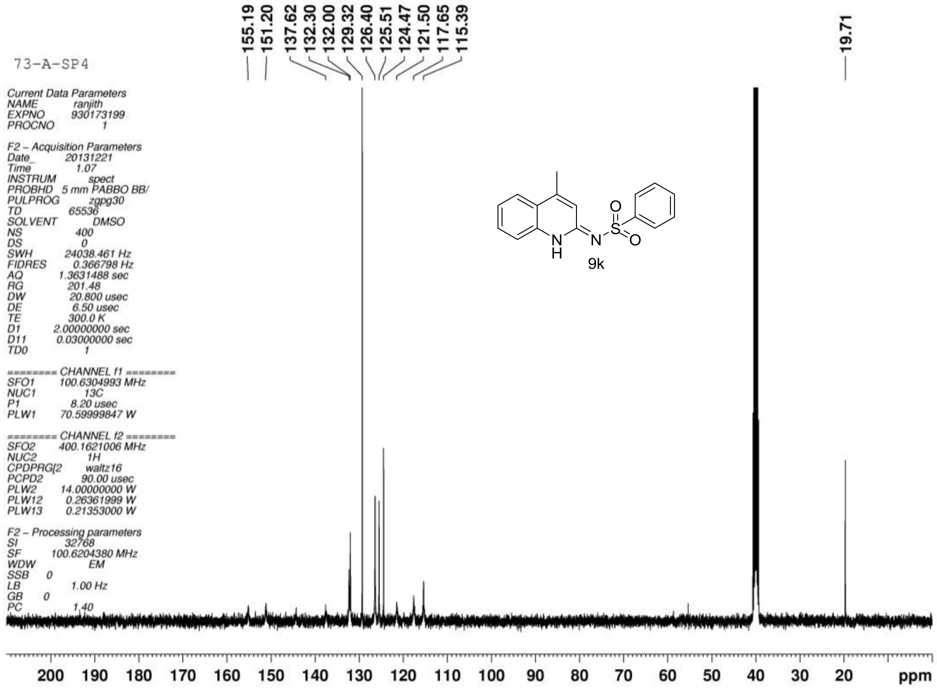
155.19 151.20 137.62 132.30 132.00 129.32 126.40 125.51 124.47 121.50 117.65 115.39
19.71
73-A-SP4
Current Data Parameters
NAME ranjith
EXPNO 930173199
PROCNO 1
F2 – Acquisition Parameters
Date_ 20131221
Time 1.07
INSTRUM spect
PROBHD 5 mm PABBO BB/
PULPROG zgpg30
TD 65536
SOLVENT DMSO
NS 400
DS 0
SWH 24038.461 Hz
FIDRES 0.366798 Hz
AQ 1.3631488 sec
RG 201.48
DW 20.800 usec
DE 6.50 usec
TE 300.0 K
D1 2.00000000 sec
D11 0.03000000 sec
TD0 1
======== CHANNEL f1 ========
SFO1 100.6304993 MHz
NUC1 13C
P1 8.20 usec
PLW1 70.59999847 W
======== CHANNEL f2 ========
SFO2 400.1621006 MHz
NUC2 1H
CPDPRG[2 waltz16
PCPD2 90.00 usec
PLW2 14.00000000 W
PLW12 0.26361999 W
PLW13 0.21353000 W
F2 – Processing parameters
SI 32768
SF 100.6204380 MHz
WDW EM
SSB 0
LB 1.00 Hz
GB 0
PC 1.40
9k
200 190 180 170 160 150 140 130 120 110 100 90 80 70 60 50 40 30 20 ppm

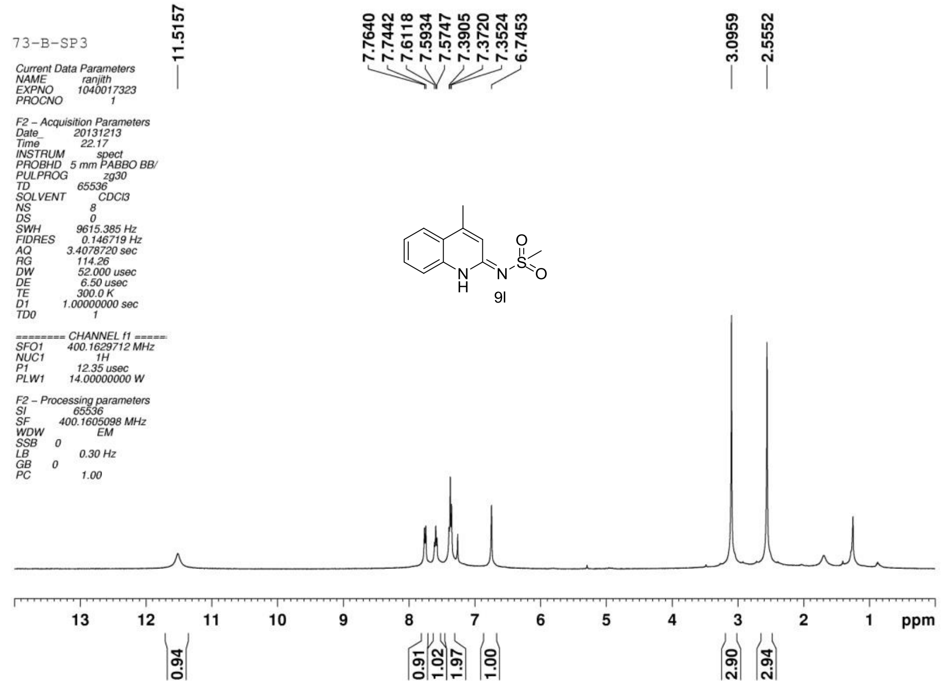
73-B-SP3
11.5157
7.7640
7.7442
7.6118
7.5934
7.5747
7.3905
7.3720
7.3524
6.7453
3.0959
2.5552
N
H
S
O
O
9l
13
12
11
10
9
8
7
6
5
4
3
2
1
ppm
0.94
0.91
1.02
1.97
1.00
2.90
2.94

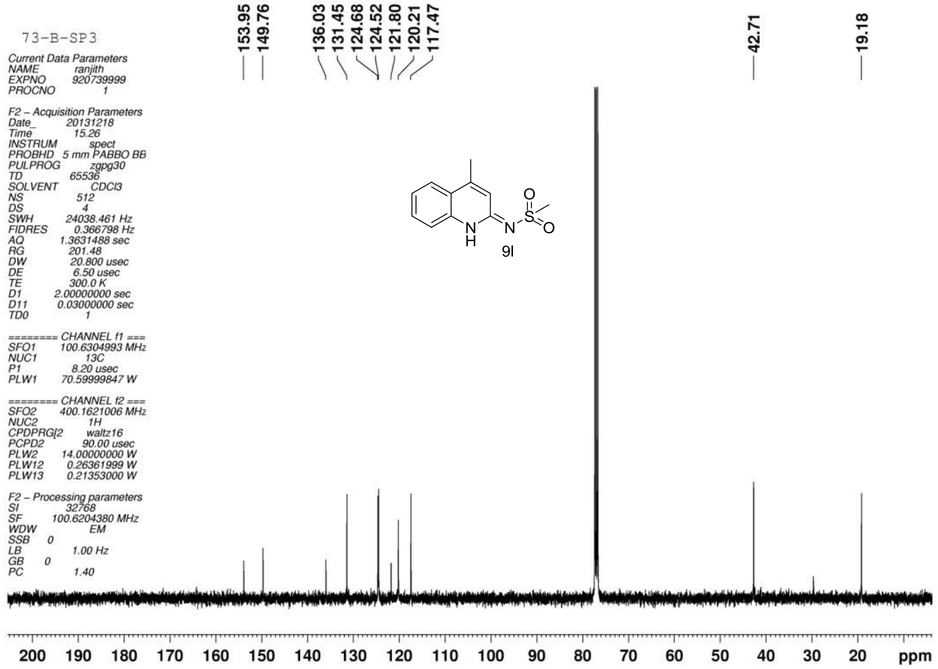
73-B-SP3
153.95
149.76
136.03
131.45
124.68
124.52
121.80
120.21
117.47
42.71
19.18
N
H
S
O
O
9l
200
190
180
170
160
150
140
130
120
110
100
90
80
70
60
50
40
30
20
ppm

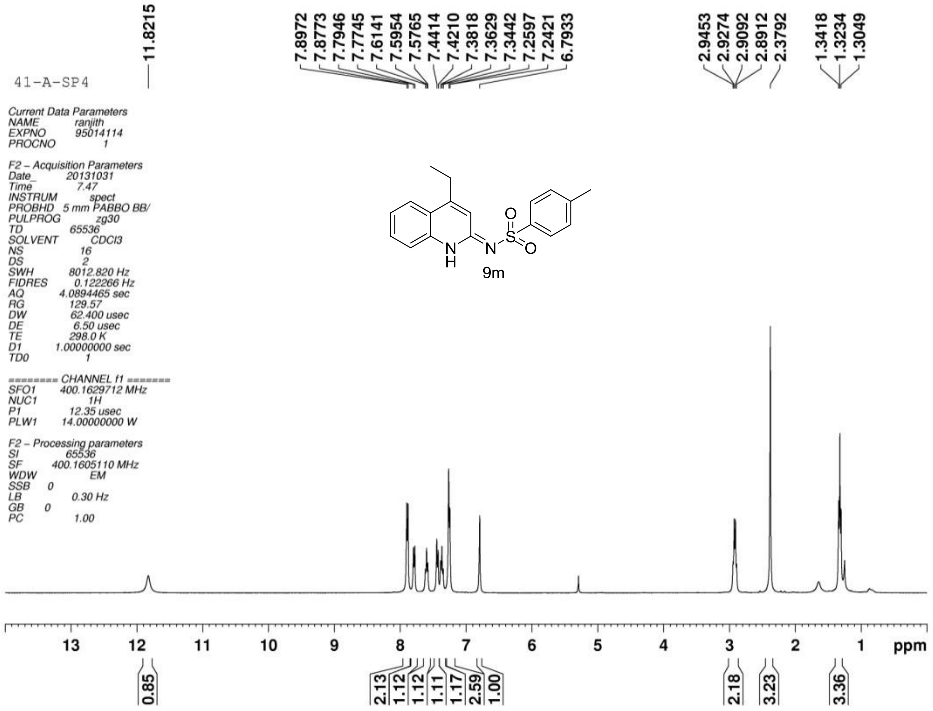
41-A-SP4
11.8215
7.8972
7.8773
7.7946
7.7745
7.6141
7.5954
7.5765
7.4414
7.4210
7.3818
7.3629
7.3442
7.2597
7.2421
6.7933
2.9453
2.9274
2.9092
2.8912
2.3792
1.3418
1.3234
1.3049
Current Data Parameters
NAME ranjith
EXPNO 95014114
PROCNO 1
F2 – Acquisition Parameters
Date_ 20131031
Time 7.47
INSTRUM spect
PROBHD 5 mm PABBO BB/
PULPROG zg30
TD 65536
SOLVENT CDCl3
NS 16
DS 2
SWH 8012.820 Hz
FIDRES 0.122266 Hz
AQ 4.0894465 sec
RG 129.57
DW 62.400 usec
DE 6.50 usec
TE 298.0 K
D1 1.00000000 sec
TD0 1
======== CHANNEL f1 ========
SFO1 400.1629712 MHz
NUC1 1H
P1 12.35 usec
PLW1 14.00000000 W
F2 – Processing parameters
SI 65536
SF 400.1605110 MHz
WDW EM
SSB 0
LB 0.30 Hz
GB 0
PC 1.00
9m
13 12 11 10 9 8 7 6 5 4 3 2 1 ppm
0.85
2.13
1.12
1.12
1.11
1.17
2.59
1.00
2.18
3.23
3.36

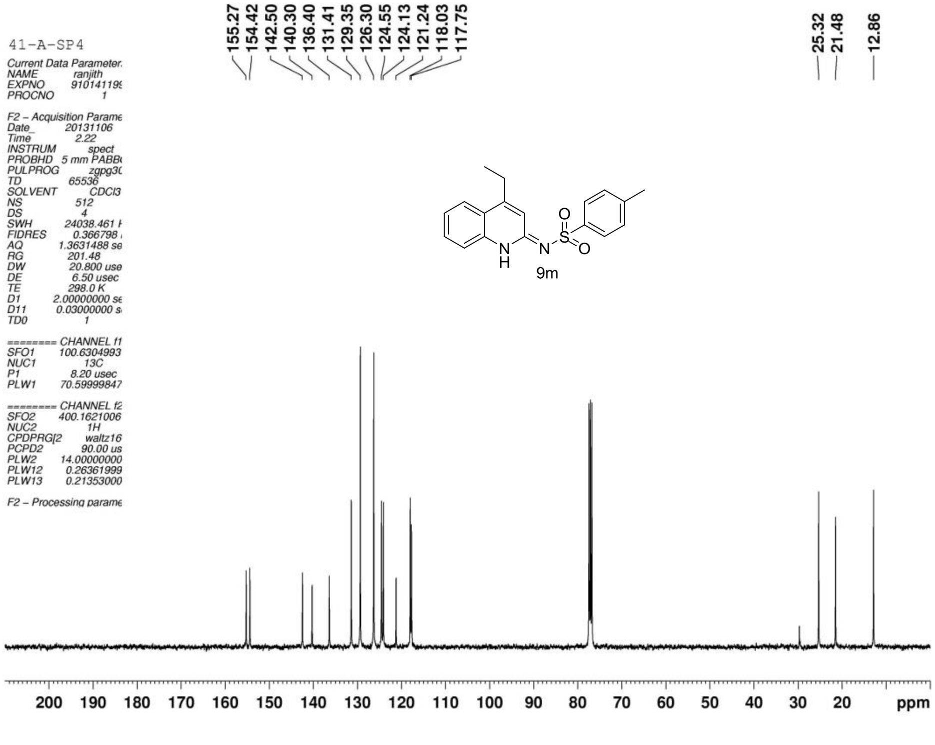
41-A-SP4
155.27
154.42
142.50
140.30
136.40
131.41
129.35
126.30
124.55
124.13
121.24
118.03
117.75
25.32
21.48
12.86
Current Data Parameter
NAME ranjith
EXPNO 91014119
PROCNO 1
F2 – Acquisition Parame
Date_ 20131106
Time 2.22
INSTRUM spect
PROBHD 5 mm PABB
PULPROG zgpg30
TD 65536
SOLVENT CDCl3
NS 512
DS 4
SWH 24038.461
FIDRES 0.366798
AQ 1.3631488 se
RG 201.48
DW 20.800 use
DE 6.50 usec
TE 298.0 K
D1 2.00000000 se
D11 0.03000000 s
TD0 1
======== CHANNEL f1
SFO1 100.6304993
NUC1 13C
P1 8.20 usec
PLW1 70.59999847
======== CHANNEL f2
SFO2 400.1621006
NUC2 1H
CPDPRG[2 waltz16
PCPD2 90.00 us
PLW2 14.00000000
PLW12 0.26361999
PLW13 0.21353000
F2 – Processing parame
9m
200 190 180 170 160 150 140 130 120 110 100 90 80 70 60 50 40 30 20 ppm

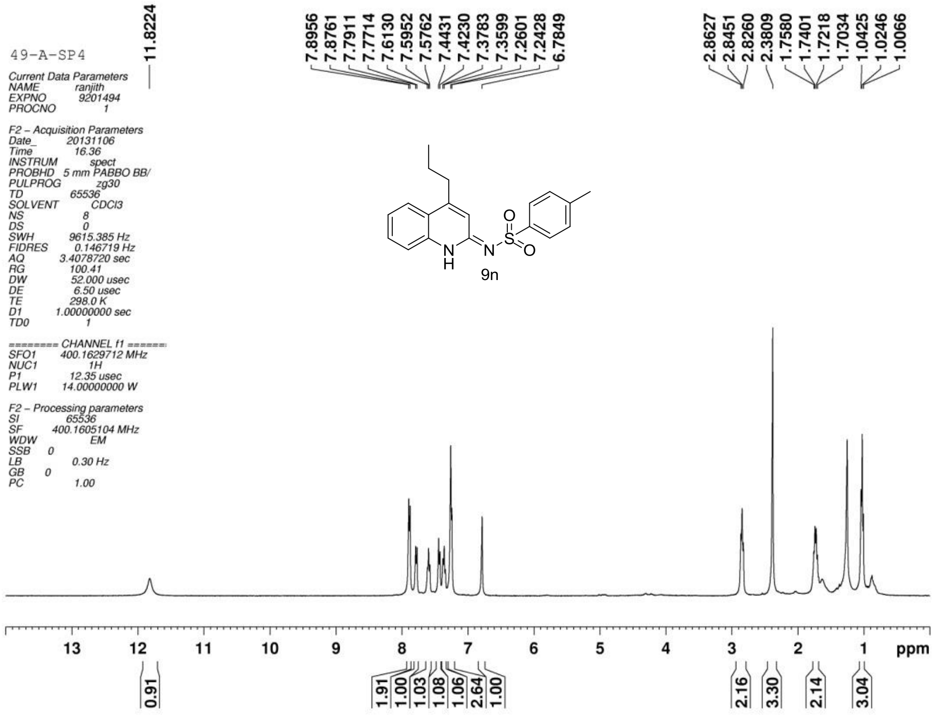
49-A-SP4
Current Data Parameters
NAME ranjith
EXPNO 9201494
PROCNO 1
F2 – Acquisition Parameters
Date_ 20131106
Time 16.36
INSTRUM spect
PROBHD 5 mm PABBO BB/
PULPROG zg30
TD 65536
SOLVENT CDCl3
NS 8
DS 0
SWH 9615.385 Hz
FIDRES 0.146719 Hz
AQ 3.4078720 sec
RG 100.41
DW 52.000 usec
DE 6.50 usec
TE 298.0 K
D1 1.00000000 sec
TD0 1
======== CHANNEL f1 ========
SFO1 400.1629712 MHz
NUC1 1H
P1 12.35 usec
PLW1 14.00000000 W
F2 – Processing parameters
SI 65536
SF 400.1605104 MHz
WDW EM
SSB 0
LB 0.30 Hz
GB 0
PC 1.00
11.8224
7.8956
7.8761
7.7911
7.7714
7.6130
7.5952
7.5762
7.4431
7.4230
7.3783
7.3599
7.2601
7.2428
6.7849
2.8627
2.8451
2.8260
2.3809
1.7580
1.7401
1.7218
1.7034
1.0425
1.0246
1.0066
N
S
O
O
N
H
9n
13 12 11 10 9 8 7 6 5 4 3 2 1 ppm
0.91
1.91
1.00
1.03
1.08
1.06
2.64
1.00
2.16
3.30
2.14
3.04

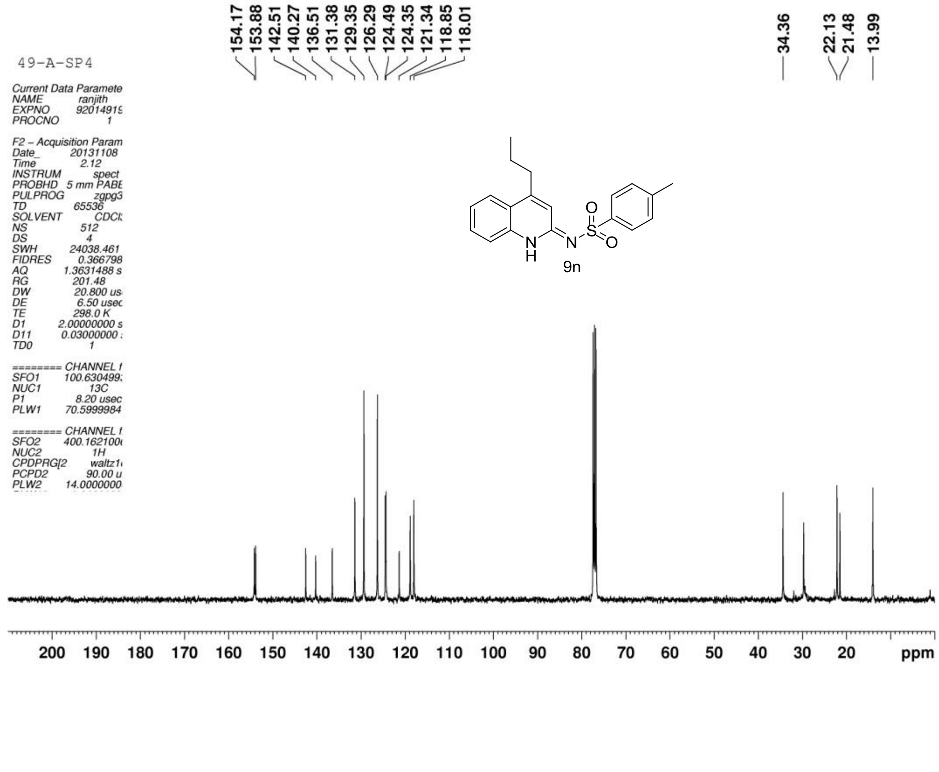
49-A-SP4
Current Data Paramete
NAME ranjith
EXPNO 92014919
PROCNO 1
F2 – Acquisition Param
Date_ 20131108
Time 2.12
INSTRUM spect
PROBHD 5 mm PABE
PULPROG zgpg3
TD 65536
SOLVENT CDCl
NS 512
DS 4
SWH 24038.461
FIDRES 0.366798
AQ 1.3631488 s
RG 201.48
DW 20.800 us
DE 6.50 usec
TE 298.0 K
D1 2.00000000 s
D11 0.03000000
TD0 1
======== CHANNEL f
SFO1 100.630499
NUC1 13C
P1 8.20 usec
PLW1 70.5999984
======== CHANNEL f
SFO2 400.16210
NUC2 1H
CPDPRG[2 waltz1
PCPD2 90.00 u
PLW2 14.0000000
154.17
153.88
142.51
140.27
136.51
131.38
129.35
126.29
124.49
124.35
121.34
118.85
118.01
34.36
22.13
21.48
13.99
N
S
O
O
N
H
9n
200 190 180 170 160 150 140 130 120 110 100 90 80 70 60 50 40 30 20 ppm

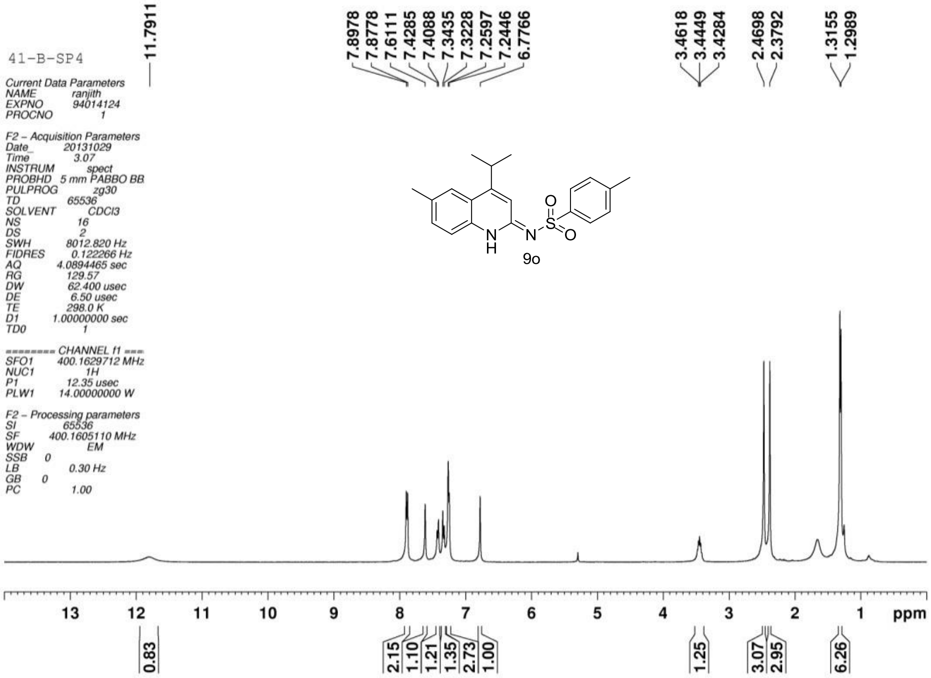
41-B-SP4
11.7911
7.8978
7.8778
7.6111
7.4285
7.4088
7.3435
7.3228
7.2597
7.2446
6.7766
3.4618
3.4449
3.4284
2.4698
2.3792
1.3155
1.2989
Current Data Parameters
NAME ranjith
EXPNO 94014124
PROCNO 1
F2 – Acquisition Parameters
Date_ 20131029
Time 3.07
INSTRUM spect
PROBHD 5 mm PABBO BB
PULPROG zg30
TD 65536
SOLVENT CDCl3
NS 16
DS 2
SWH 8012.820 Hz
FIDRES 0.122266 Hz
AQ 4.0894465 sec
RG 129.57
DW 62.400 usec
DE 6.50 usec
TE 298.0 K
D1 1.00000000 sec
TD0 1
======== CHANNEL f1 ===
SFO1 400.1629712 MHz
NUC1 1H
P1 12.35 usec
PLW1 14.00000000 W
F2 – Processing parameters
SI 65536
SF 400.1605110 MHz
WDW EM
SSB 0
LB 0.30 Hz
GB 0
PC 1.00
N
H
N
S
O
O
9o
13
12
11
10
9
8
7
6
5
4
3
2
1
ppm
0.83
2.15
1.10
1.21
1.35
2.73
1.00
1.25
3.07
2.95
6.26

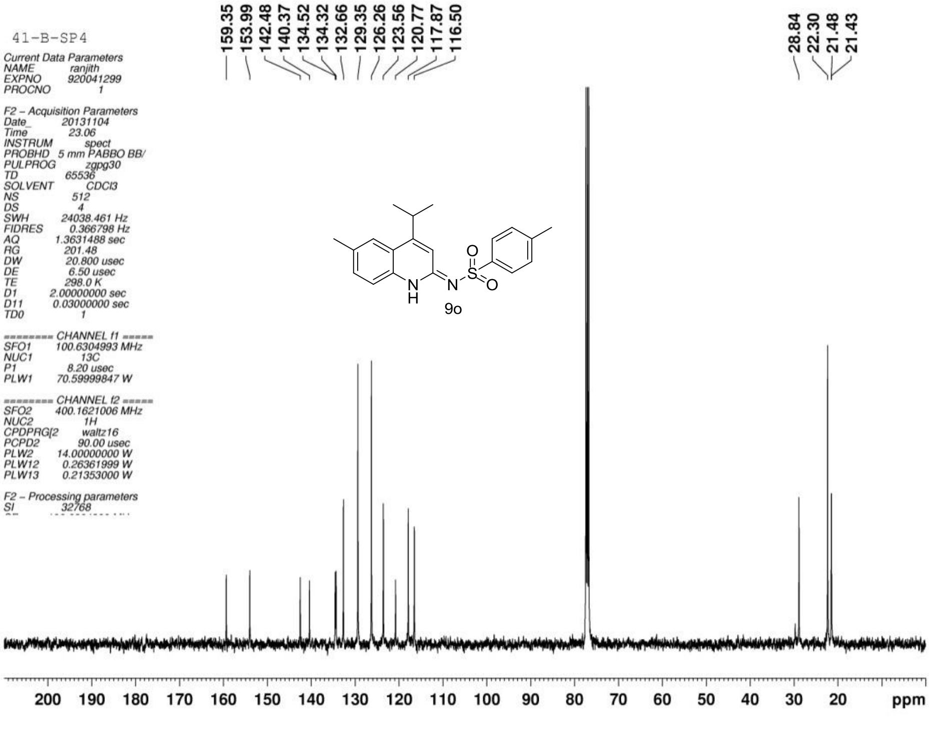
41-B-SP4
159.35
153.99
142.48
140.37
134.52
134.32
132.66
129.35
126.26
123.56
120.77
117.87
116.50
28.84
22.30
21.48
21.43
Current Data Parameters
NAME ranjith
EXPNO 920041299
PROCNO 1
F2 – Acquisition Parameters
Date_ 20131104
Time 23.06
INSTRUM spect
PROBHD 5 mm PABBO BB/
PULPROG zgpg30
TD 65536
SOLVENT CDCl3
NS 512
DS 4
SWH 24038.461 Hz
FIDRES 0.366798 Hz
AQ 1.3631488 sec
RG 201.48
DW 20.800 usec
DE 6.50 usec
TE 298.0 K
D1 2.00000000 sec
D11 0.03000000 sec
TD0 1
======== CHANNEL f1 =====
SFO1 100.6304993 MHz
NUC1 13C
P1 8.20 usec
PLW1 70.59999847 W
======== CHANNEL f2 =====
SFO2 400.1621006 MHz
NUC2 1H
CPDPRG[2 waltz16
PCPD2 90.00 usec
PLW2 14.00000000 W
PLW12 0.26361999 W
PLW13 0.21353000 W
F2 – Processing parameters
SI 32768
N
H
N
S
O
O
9o
200
190
180
170
160
150
140
130
120
110
100
90
80
70
60
50
40
30
20
ppm

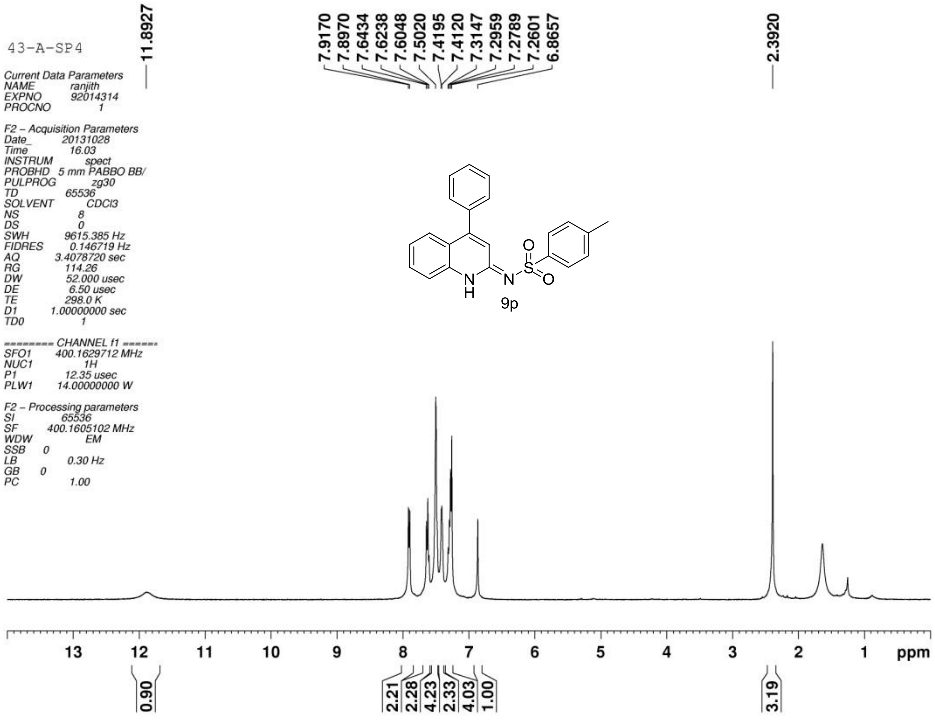
43-A-SP4
Current Data Parameters
NAME ranjith
EXPNO 92014314
PROCNO 1
F2 – Acquisition Parameters
Date_ 20131028
Time 16.03
INSTRUM spect
PROBHD 5 mm PABBO BB/
PULPROG zg30
TD 65536
SOLVENT CDCl3
NS 8
DS 0
SWH 9615.385 Hz
FIDRES 0.146719 Hz
AQ 3.4078720 sec
RG 114.26
DW 52.000 usec
DE 6.50 usec
TE 298.0 K
D1 1.00000000 sec
TD0 1
======== CHANNEL f1 ======
SFO1 400.1629712 MHz
NUC1 1H
P1 12.35 usec
PLW1 14.00000000 W
F2 – Processing parameters
SI 65536
SF 400.1605102 MHz
WDW EM
SSB 0
LB 0.30 Hz
GB 0
PC 1.00
11.8927
7.9170
7.8970
7.6434
7.6238
7.6048
7.5020
7.4195
7.4120
7.3147
7.2959
7.2789
7.2601
6.8657
2.3920
O
S
N
O
N
H
9p
13 12 11 10 9 8 7 6 5 4 3 2 1 ppm
0.90
2.21
2.28
4.23
2.33
4.03
1.00
3.19

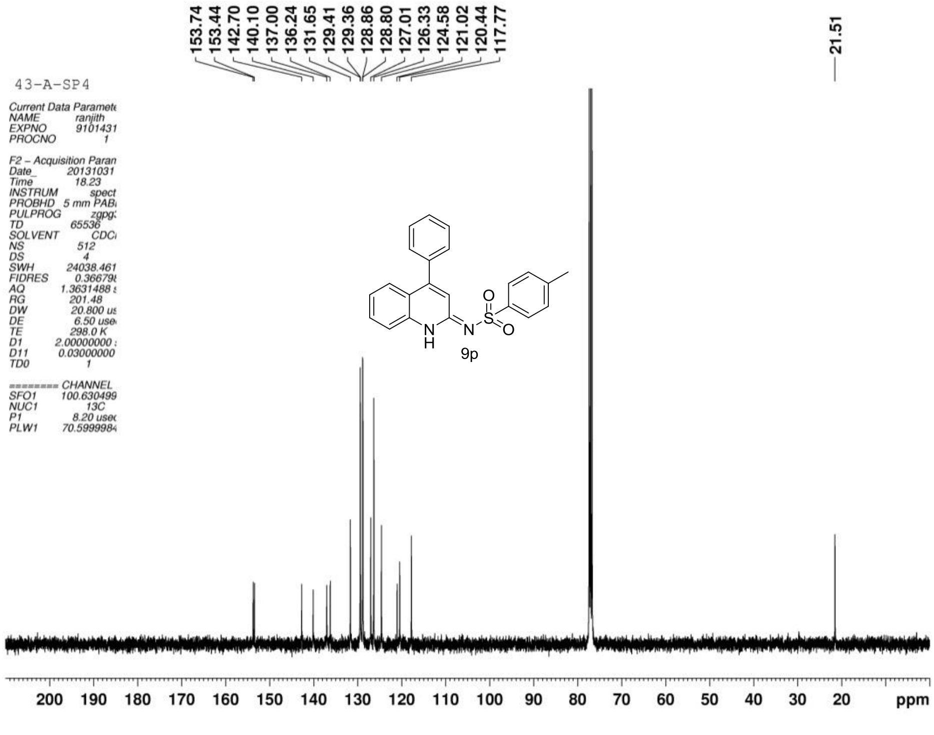
43-A-SP4
Current Data Paramete
NAME ranjith
EXPNO 9101431
PROCNO 1
F2 – Acquisition Paran
Date_ 20131031
Time 18.23
INSTRUM spect
PROBHD 5 mm PAB
PULPROG zgpg
TD 65536
SOLVENT CDC
NS 512
DS 4
SWH 24038.461
FIDRES 0.36679
AQ 1.3631488
RG 201.48
DW 20.800 us
DE 6.50 use
TE 298.0 K
D1 2.00000000
D11 0.03000000
TD0 1
======== CHANNEL
SFO1 100.630499
NUC1 13C
P1 8.20 usec
PLW1 70.599998
153.74
153.44
142.70
140.10
137.00
136.24
131.65
129.41
129.36
128.86
128.80
127.01
126.33
124.58
121.02
120.44
117.77
21.51
O
S
N
O
N
H
9p
200 190 180 170 160 150 140 130 120 110 100 90 80 70 60 50 40 30 20 ppm

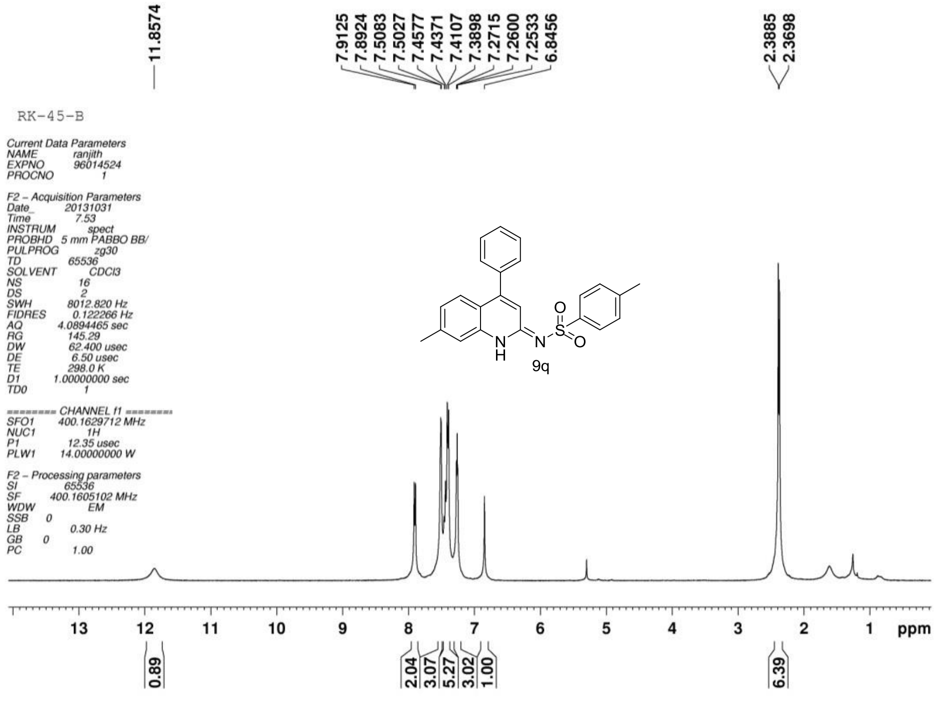
11.8574
7.9125
7.8924
7.5083
7.5027
7.4577
7.4371
7.4107
7.3898
7.2715
7.2600
7.2533
6.8456
2.3885
2.3698
RK-45-B
Current Data Parameters
NAME ranjith
EXPNO 96014524
PROCNO 1
F2 – Acquisition Parameters
Date_ 20131031
Time 7.53
INSTRUM spect
PROBHD 5 mm PABBO BB/
PULPROG zg30
TD 65536
SOLVENT CDCl3
NS 16
DS 2
SWH 8012.820 Hz
FIDRES 0.122266 Hz
AQ 4.0894465 sec
RG 145.29
DW 62.400 usec
DE 6.50 usec
TE 298.0 K
D1 1.00000000 sec
TD0 1
======= CHANNEL f1 =======
SFO1 400.1629712 MHz
NUC1 1H
P1 12.35 usec
PLW1 14.00000000 W
F2 – Processing parameters
SI 65536
SF 400.1605102 MHz
WDW EM
SSB 0
LB 0.30 Hz
GB 0
PC 1.00
N
H
N
S
O
O
9q
13 12 11 10 9 8 7 6 5 4 3 2 1 ppm
0.89
2.04
3.07
5.27
3.02
1.00
6.39

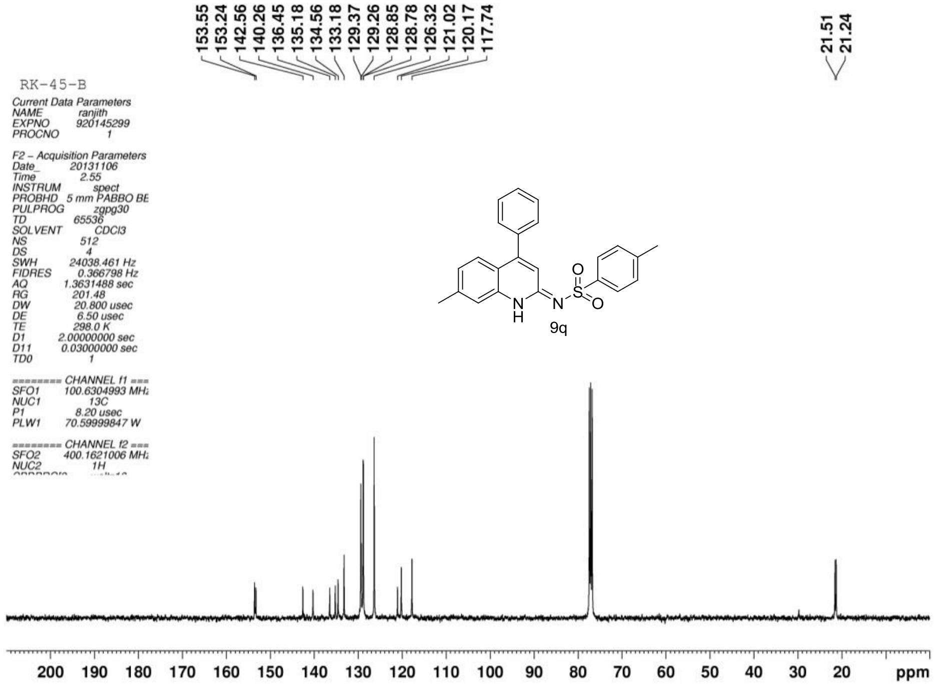
153.55
153.24
142.56
140.26
136.45
135.18
134.56
133.18
129.37
129.26
128.85
128.78
126.32
121.02
120.17
117.74
21.51
21.24
RK-45-B
Current Data Parameters
NAME ranjith
EXPNO 920145299
PROCNO 1
F2 – Acquisition Parameters
Date_ 20131106
Time 2.55
INSTRUM spect
PROBHD 5 mm PABBO BE
PULPROG zgpg30
TD 65536
SOLVENT CDCl3
NS 512
DS 4
SWH 24038.461 Hz
FIDRES 0.366798 Hz
AQ 1.3631488 sec
RG 201.48
DW 20.800 usec
DE 6.50 usec
TE 298.0 K
D1 2.00000000 sec
D11 0.03000000 sec
TD0 1
======= CHANNEL f1 ===
SFO1 100.6304993 MH:
NUC1 13C
P1 8.20 usec
PLW1 70.59999847 W
======= CHANNEL f2 ===
SFO2 400.1621006 MH:
NUC2 1H
N
H
N
S
O
O
9q
200 190 180 170 160 150 140 130 120 110 100 90 80 70 60 50 40 30 20 ppm

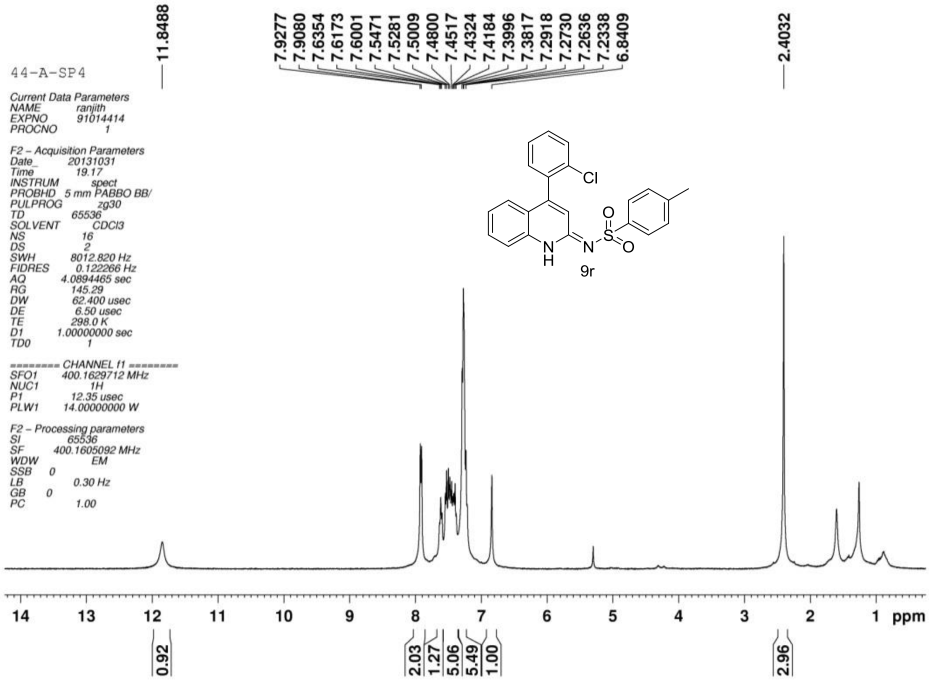
44-A-SP4
11.8488
7.9277
7.9080
7.6354
7.6173
7.6001
7.5471
7.5281
7.5009
7.4800
7.4517
7.4324
7.4184
7.3996
7.3817
7.2918
7.2730
7.2636
7.2338
6.8409
2.4032
Current Data Parameters
NAME ranjith
EXPNO 91014414
PROCNO 1
F2 – Acquisition Parameters
Date_ 20131031
Time 19.17
INSTRUM spect
PROBHD 5 mm PABBO BB/
PULPROG zg30
TD 65536
SOLVENT CDCl3
NS 16
DS 2
SWH 8012.820 Hz
FIDRES 0.122266 Hz
AQ 4.0894465 sec
RG 145.29
DW 62.400 usec
DE 6.50 usec
TE 298.0 K
D1 1.00000000 sec
TD0 1
======= CHANNEL f1 =======
SFO1 400.1629712 MHz
NUC1 1H
P1 12.35 usec
PLW1 14.00000000 W
F2 – Processing parameters
SI 65536
SF 400.1605092 MHz
WDW EM
SSB 0
LB 0.30 Hz
GB 0
PC 1.00
Cl
N
S
O
O
N
H
9r
14 13 12 11 10 9 8 7 6 5 4 3 2 1 ppm
0.92
2.03
1.27
5.06
5.49
1.00
2.96

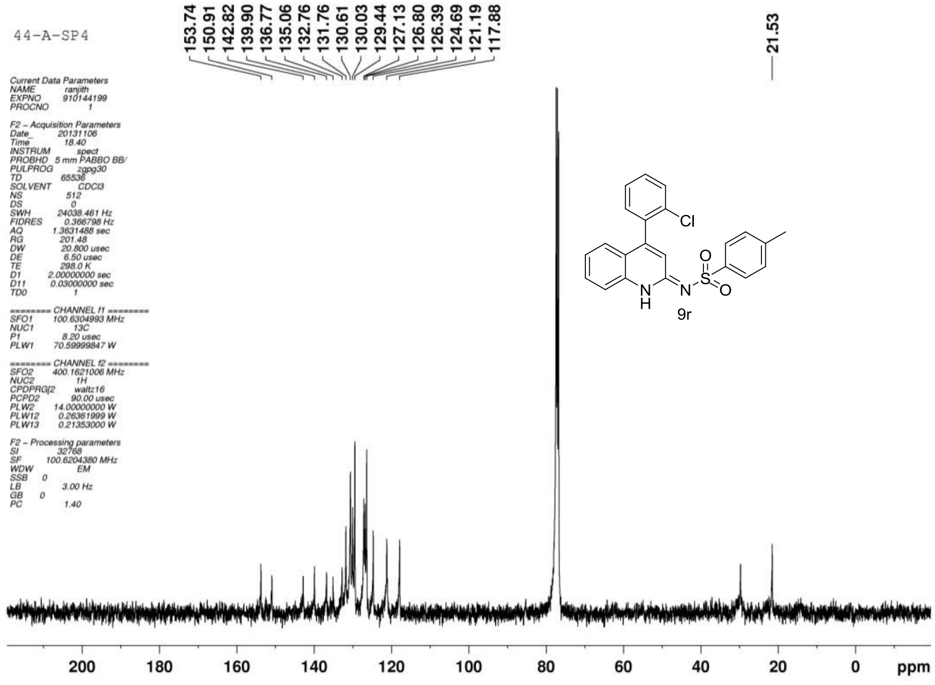
44-A-SP4
153.74
150.91
142.82
139.90
136.77
135.06
132.76
131.76
130.61
130.03
129.44
127.13
126.80
126.39
124.69
121.19
117.88
21.53
Current Data Parameters
NAME ranjith
EXPNO 910144199
PROCNO 1
F2 – Acquisition Parameters
Date_ 20131106
Time 18.40
INSTRUM spect
PROBHD 5 mm PABBO BB/
PULPROG zgpg30
TD 65536
SOLVENT CDCl3
NS 512
DS 0
SWH 24038.461 Hz
FIDRES 0.366798 Hz
AQ 1.3631488 sec
RG 201.48
DW 20.800 usec
DE 6.50 usec
TE 298.0 K
D1 2.00000000 sec
D11 0.03000000 sec
TD0 1
======= CHANNEL f1 =======
SFO1 100.6304993 MHz
NUC1 13C
P1 8.20 usec
PLW1 70.59999847 W
======= CHANNEL f2 =======
SFO2 400.1621006 MHz
NUC2 1H
CPDPRG[2 waltz16
PCPD2 90.00 usec
PLW2 14.00000000 W
PLW12 0.26361999 W
PLW13 0.21353000 W
F2 – Processing parameters
SI 32768
SF 100.6204380 MHz
WDW EM
SSB 0
LB 3.00 Hz
GB 0
PC 1.40
Cl
N
S
O
O
N
H
9r
200 180 160 140 120 100 80 60 40 20 0 ppm

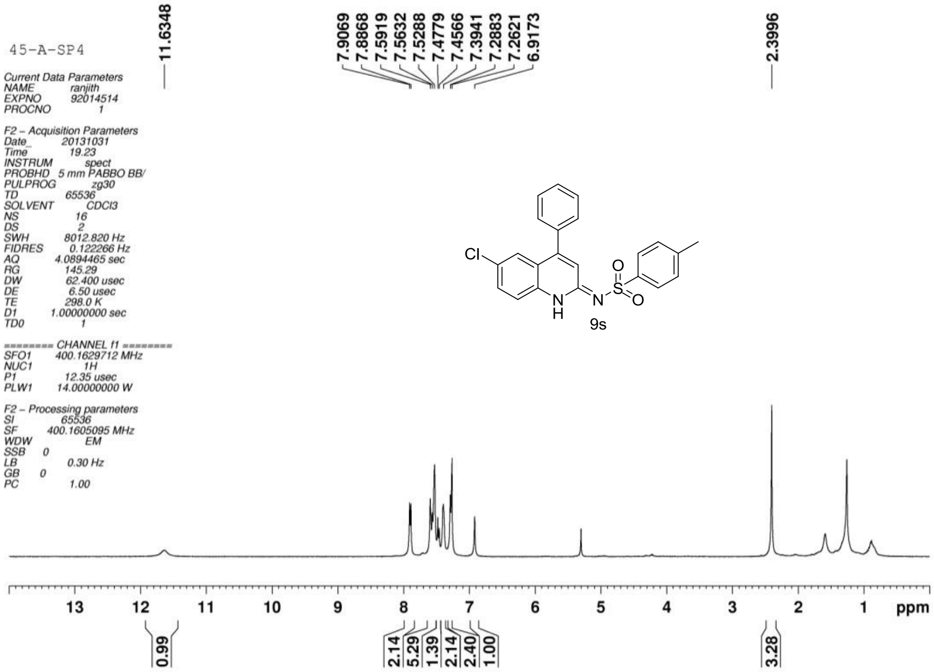
45-A-SP4
11.6348
7.9069
7.8868
7.5919
7.5632
7.5288
7.4779
7.4566
7.3941
7.2883
7.2621
6.9173
2.3996
Current Data Parameters
NAME ranjith
EXPNO 92014514
PROCNO 1
F2 – Acquisition Parameters
Date_ 20131031
Time 19.23
INSTRUM spect
PROBHD 5 mm PABBO BB/
PULPROG zg30
TD 65536
SOLVENT CDCl3
NS 16
DS 2
SWH 8012.820 Hz
FIDRES 0.122266 Hz
AQ 4.0894465 sec
RG 145.29
DW 62.400 usec
DE 6.50 usec
TE 298.0 K
D1 1.00000000 sec
TD0 1
======= CHANNEL f1 =======
SFO1 400.1629712 MHz
NUC1 1H
P1 12.35 usec
PLW1 14.00000000 W
F2 – Processing parameters
SI 65536
SF 400.1605095 MHz
WDW EM
SSB 0
LB 0.30 Hz
GB 0
PC 1.00
Cl
N
H
N
S
O
O
9s
13
12
11
10
9
8
7
6
5
4
3
2
1
ppm
0.99
2.14
5.29
1.39
2.14
2.40
1.00
3.28

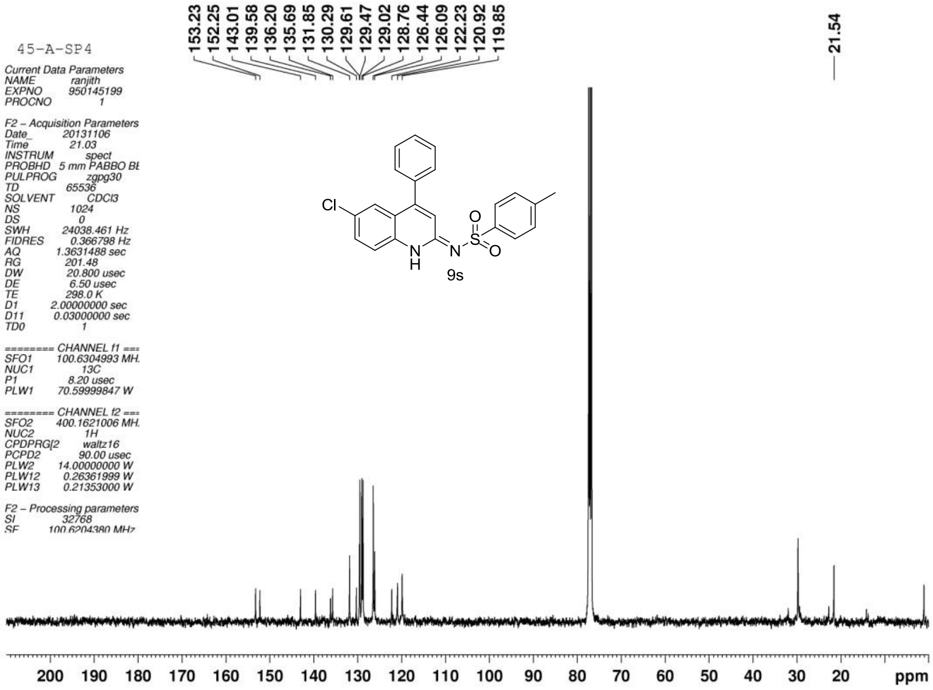
45-A-SP4
153.23
152.25
143.01
139.58
136.20
135.69
131.85
130.29
129.61
129.47
129.02
128.76
126.44
126.09
122.23
120.92
119.85
21.54
Current Data Parameters
NAME ranjith
EXPNO 950145199
PROCNO 1
F2 – Acquisition Parameters
Date_ 20131106
Time 21.03
INSTRUM spect
PROBHD 5 mm PABBO BB
PULPROG zgpg30
TD 65536
SOLVENT CDCl3
NS 1024
DS 0
SWH 24038.461 Hz
FIDRES 0.366798 Hz
AQ 1.3631488 sec
RG 201.48
DW 20.800 usec
DE 6.50 usec
TE 298.0 K
D1 2.00000000 sec
D11 0.03000000 sec
TD0 1
======= CHANNEL f1 ===
SFO1 100.6304993 MH
NUC1 13C
P1 8.20 usec
PLW1 70.59999847 W
======= CHANNEL f2 ===
SFO2 400.1621006 MH
NUC2 1H
CPDPRG[2 waltz16
PCPD2 90.00 usec
PLW2 14.00000000 W
PLW12 0.26361999 W
PLW13 0.21353000 W
F2 – Processing parameters
SI 32768
SF 100.6204380 MHz
Cl
N
H
N
S
O
O
9s
200
190
180
170
160
150
140
130
120
110
100
90
80
70
60
50
40
30
20
ppm

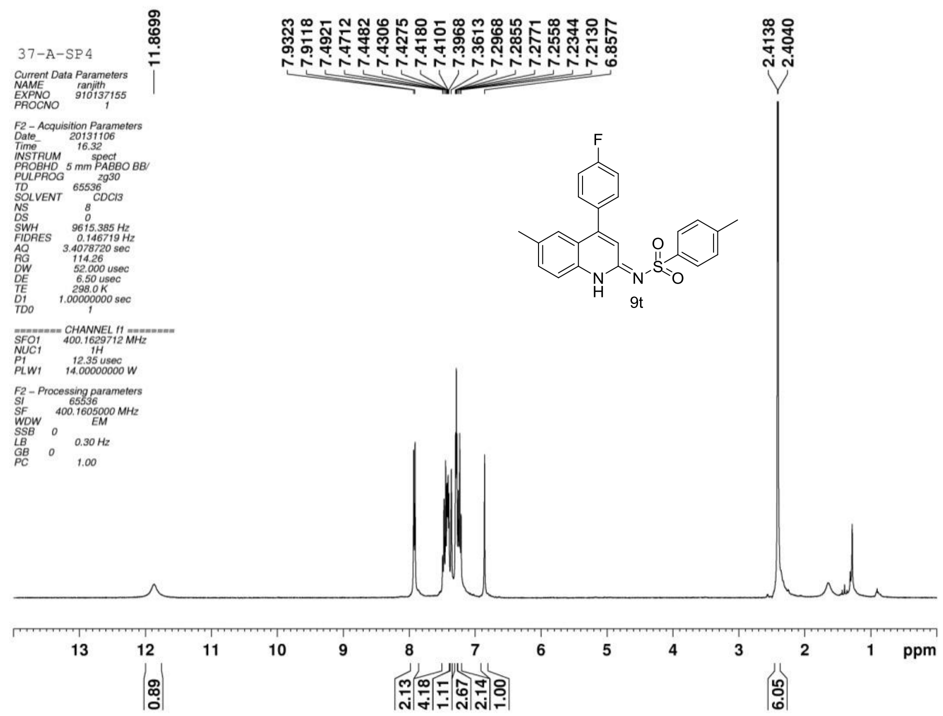
37-A-SP4
Current Data Parameters
NAME ranjith
EXPNO 910137155
PROCNO 1
F2 – Acquisition Parameters
Date_ 20131106
Time 16.32
INSTRUM spect
PROBHD 5 mm PABBO BB/
PULPROG zg30
TD 65536
SOLVENT CDCl3
NS 8
DS 0
SWH 9615.385 Hz
FIDRES 0.146719 Hz
AQ 3.4078720 sec
RG 114.26
DW 52.000 usec
DE 6.50 usec
TE 298.0 K
D1 1.00000000 sec
TD0 1
======== CHANNEL f1 ========
SFO1 400.1629712 MHz
NUC1 1H
P1 12.35 usec
PLW1 14.00000000 W
F2 – Processing parameters
SI 65536
SF 400.1605000 MHz
WDW EM
SSB 0
LB 0.30 Hz
GB 0
PC 1.00
11.8699
7.9323
7.9118
7.4921
7.4712
7.4482
7.4306
7.4275
7.4180
7.4101
7.3968
7.3613
7.2968
7.2855
7.2771
7.2558
7.2344
7.2130
6.8577
2.4138
2.4040
F
N
S
O
O
N
H
9t
13
12
11
10
9
8
7
6
5
4
3
2
1
ppm
0.89
2.13
4.18
1.11
2.67
2.14
1.00
6.05

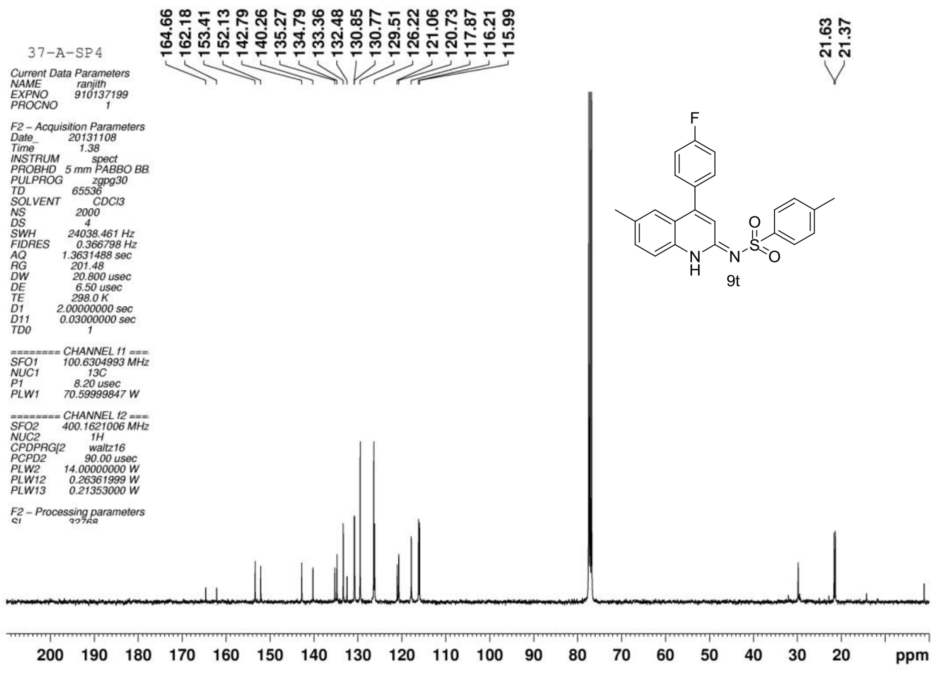
37-A-SP4
Current Data Parameters
NAME ranjith
EXPNO 910137199
PROCNO 1
F2 – Acquisition Parameters
Date_ 20131108
Time 1.38
INSTRUM spect
PROBHD 5 mm PABBO BB
PULPROG zgpg30
TD 65536
SOLVENT CDCl3
NS 2000
DS 4
SWH 24038.461 Hz
FIDRES 0.366798 Hz
AQ 1.3631488 sec
RG 201.48
DW 20.800 usec
DE 6.50 usec
TE 298.0 K
D1 2.00000000 sec
D11 0.03000000 sec
TD0 1
======== CHANNEL f1 ===
SFO1 100.6304993 MHz
NUC1 13C
P1 8.20 usec
PLW1 70.59999847 W
======== CHANNEL f2 ===
SFO2 400.1621006 MHz
NUC2 1H
CPDPRG[2 waltz16
PCPD2 90.00 usec
PLW2 14.00000000 W
PLW12 0.26361999 W
PLW13 0.21353000 W
F2 – Processing parameters
164.66
162.18
153.41
152.13
142.79
140.26
135.27
134.79
133.36
132.48
130.85
130.77
129.51
126.22
121.06
120.73
117.87
116.21
115.99
21.63
21.37
F
N
S
O
O
N
H
9t
200
190
180
170
160
150
140
130
120
110
100
90
80
70
60
50
40
30
20
ppm

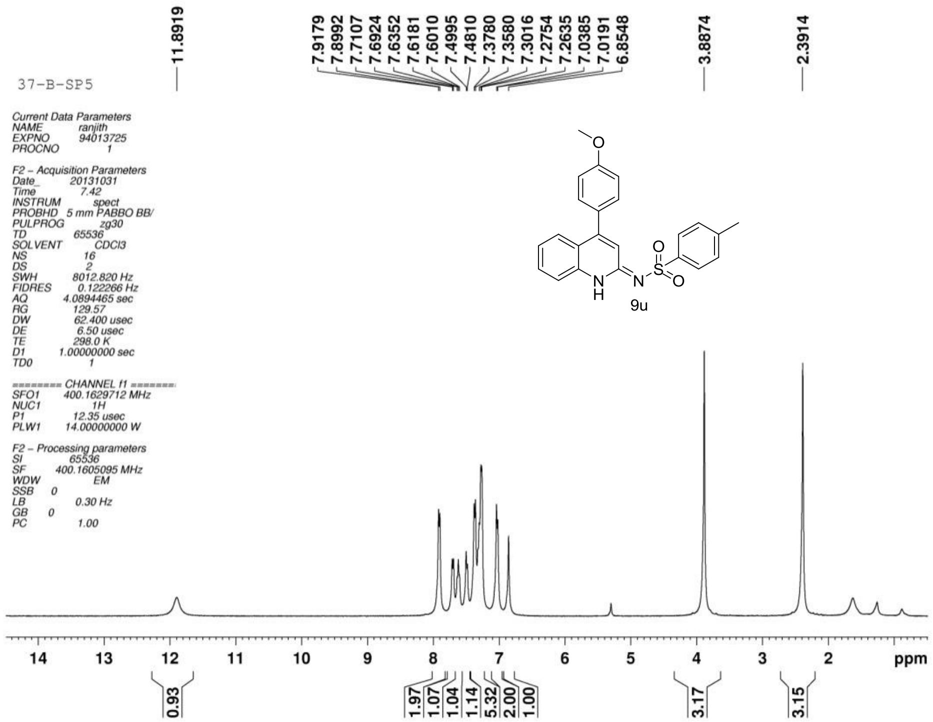
37-B-SP5
11.8919
7.9179
7.8992
7.7107
7.6924
7.6352
7.6181
7.6010
7.4995
7.4810
7.3780
7.3580
7.3016
7.2754
7.2635
7.0385
7.0191
6.8548
3.8874
2.3914
Current Data Parameters
NAME ranjith
EXPNO 94013725
PROCNO 1
F2 - Acquisition Parameters
Date_ 20131031
Time 7.42
INSTRUM spect
PROBHD 5 mm PABBO BB/
PULPROG zg30
TD 65536
SOLVENT CDCl3
NS 16
DS 2
SWH 8012.820 Hz
FIDRES 0.122266 Hz
AQ 4.0894465 sec
RG 129.57
DW 62.400 usec
DE 6.50 usec
TE 298.0 K
D1 1.00000000 sec
TD0 1
======== CHANNEL f1 ========
SFO1 400.1629712 MHz
NUC1 1H
P1 12.35 usec
PLW1 14.00000000 W
F2 - Processing parameters
SI 65536
SF 400.1605095 MHz
WDW EM
SSB 0
LB 0.30 Hz
GB 0
PC 1.00
9u
14 13 12 11 10 9 8 7 6 5 4 3 2 ppm
0.93
1.97
1.07
1.04
1.14
5.32
2.00
1.00
3.17
3.15

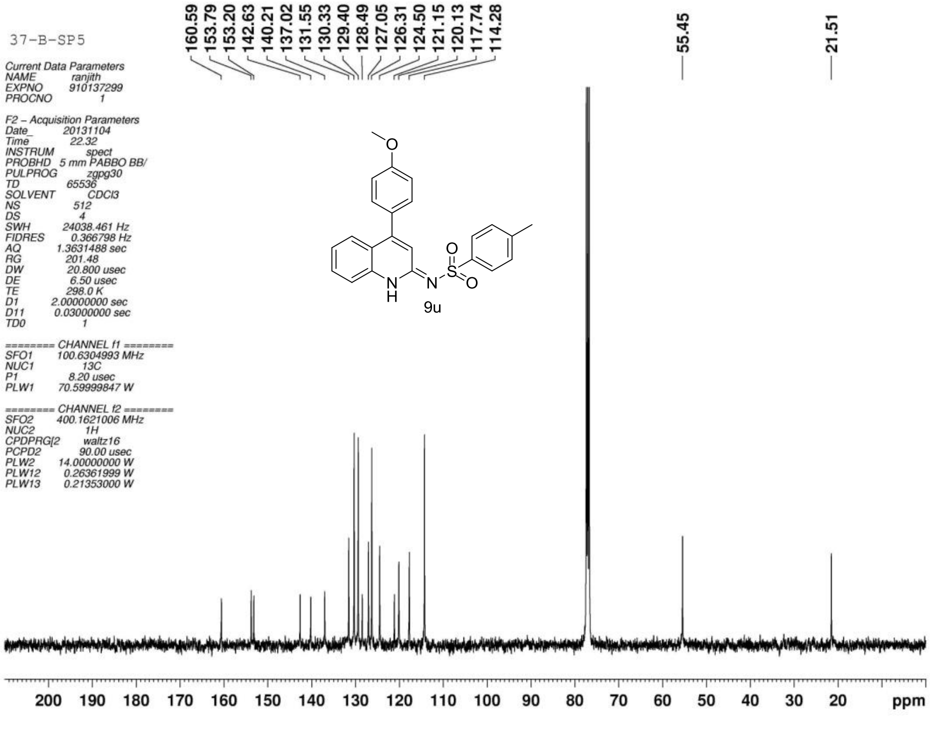
37-B-SP5
160.59
153.79
153.20
142.63
140.21
137.02
131.55
130.33
129.40
128.49
127.05
126.31
124.50
121.15
120.13
117.74
114.28
55.45
21.51
Current Data Parameters
NAME ranjith
EXPNO 910137299
PROCNO 1
F2 - Acquisition Parameters
Date_ 20131104
Time 22.32
INSTRUM spect
PROBHD 5 mm PABBO BB/
PULPROG zgpg30
TD 65536
SOLVENT CDCl3
NS 512
DS 4
SWH 24038.461 Hz
FIDRES 0.366798 Hz
AQ 1.3631488 sec
RG 201.48
DW 20.800 usec
DE 6.50 usec
TE 298.0 K
D1 2.00000000 sec
D11 0.03000000 sec
TD0 1
======== CHANNEL f1 ========
SFO1 100.6304993 MHz
NUC1 13C
P1 8.20 usec
PLW1 70.59999847 W
======== CHANNEL f2 ========
SFO2 400.1621006 MHz
NUC2 1H
CPDPRG[2 waltz16
PCPD2 90.00 usec
PLW2 14.00000000 W
PLW12 0.26361999 W
PLW13 0.21353000 W
9u
200 190 180 170 160 150 140 130 120 110 100 90 80 70 60 50 40 30 20 ppm

Detailed write up for the X-ray structure of 9j (CCDC number 971729)

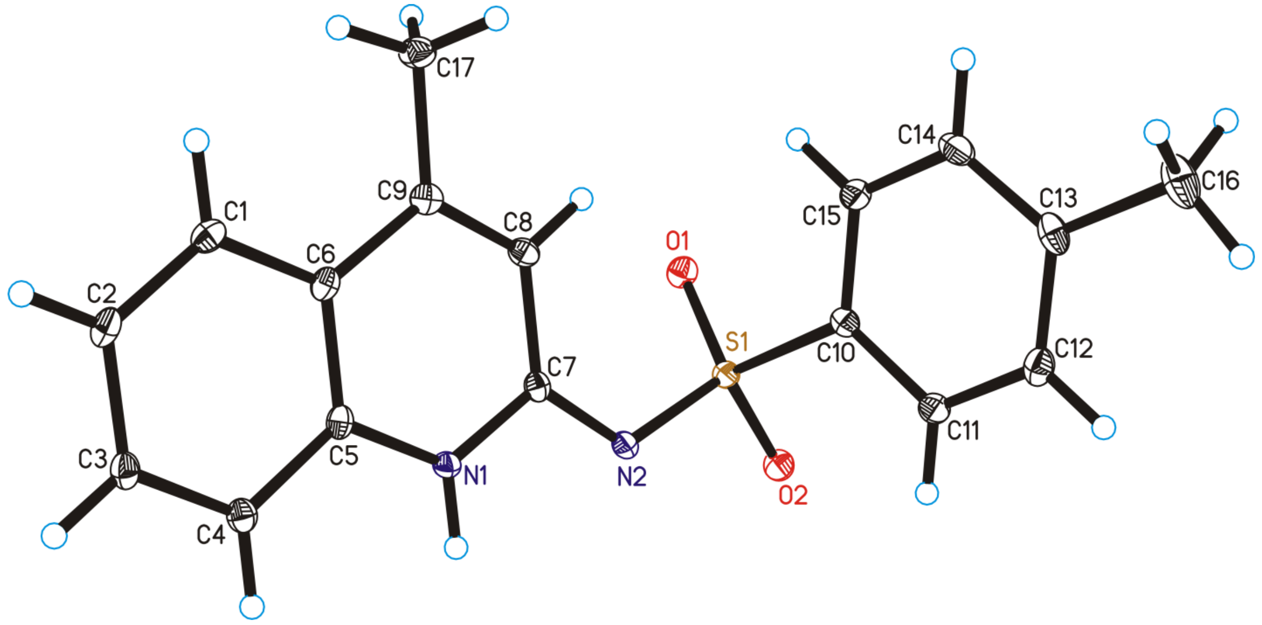

Figure S1 ORTEP diagram drawn with 30% ellipsoid probability for non-H atoms of the crystal structure of compound 9j determined at 293 K.

Crystallization: Crystals of compound **9j** were grown from the solvent EtOAc by slow evaporation method.

X-ray data collection and structure refinement details:

A good quality yellowish color single crystal of size 0.33 × 0.21 × 0.06 mm, was selected under a polarizing microscope and was mounted on a glass fiber for data collection. Single crystal X-ray data for compound **9j** were collected on the Rigaku Kappa 3 circle diffractometer equipped with the AFC12 goniometer and enhanced sensitivity (HG) Saturn724+ CCD detector in the 4 × 4 bin mode using the monochromated Mo-Kα radiation generated from the microfocus sealed tube MicroMax-003 X-ray generator equipped with specially designed confocal multilayer optics. Data collection was performed using ω-scans of 0.5° steps at 293(2) K. Cell determination, data collection and data reduction was performed using the Rigaku CrystalClear-SM Expert 2.1 b24[1] software. Structure solution and refinement were performed by using SHELXTL-NT[2]. Refinement of coordinates and anisotropic thermal parameters of non-hydrogen atoms were carried out by the full-matrix least-squares method. The hydrogen atoms attached to carbon atoms were generated with idealized geometries and isotropically refined using a riding model.

1. CrystalClear 2.1, Rigaku Corporation, Tokyo, Japan
2. Sheldrick, G. M. Acta Crystallogr., Sect. A 2008, 64, 112–122.

Table S1 Crystal data and structure refinement details for **9j**.

**Compound**	********
Empirical formula	C_{17} H_{16} N_2 S
Formula weight	312.38
Crystal System	Triclinc
Space group	*P*-1
a (Å)	25.116(11)
b (Å)	14.416(7)
c (Å)	8.406(4)
α (°)	90.00
β (°)	90.00
γ (°)	90.00
V (Å^3)	3044(2)
Z	8
D_c (g/cm^3)	1.363
F_{000}	1312
μ (mm^{-1})	0.221
θ_{max} (°)	25.38
Total reflections	21951
Unique reflections	2781
Reflections [$I > 2\sigma(I)$]	2481
Parameters	203
R_{int}	0.0414
Goodness-of-fit	1.097
R [$F^2 > 2\sigma(F^2)$]	0.0619
wR (F^2, all data)	0.1509
CCDC No.	971729

Bond lengths and angles

C1 - Distance Angles

C2 1.3759 (0.0043)

C6 1.4038 (0.0043) 120.98 (0.29)

H1 0.9300 119.51 119.51

C1 - C2 C6

C2 - Distance Angles

C1 1.3759 (0.0043)

C3 1.3991 (0.0044) 119.43 (0.29)

H2 0.9300 120.28 120.28

C2 - C1 C3

C3 - Distance Angles

C4 1.3769 (0.0044)

C2 1.3991 (0.0044) 121.37 (0.28)

H3 0.9300 119.31 119.31

C3 - C4 C2

C4 - Distance Angles

C3 1.3769 (0.0044)

C5 1.4030 (0.0040) 119.16 (0.29)

H4 0.9300 120.42 120.42

C4 - C3 C5

C5 -	Distance	Angles	
N1	1.3857 (0.0040)		
C4	1.4030 (0.0040)	119.95 (0.27)	
C6	1.4090 (0.0042)	119.69 (0.26)	120.36 (0.28)
	C5 -	N1	C4

C6 -	Distance	Angles	
C1	1.4038 (0.0043)		
C5	1.4090 (0.0042)	118.69 (0.27)	
C9	1.4455 (0.0041)	123.52 (0.28)	117.80 (0.27)
	C6 -	C1	C5

C7 -	Distance	Angles	
N2	1.3368 (0.0039)		
N1	1.3571 (0.0036)	114.94 (0.26)	
C8	1.4344 (0.0041)	128.81 (0.26)	116.24 (0.27)
	C7 -	N2	N1

C8 -	Distance	Angles	
C9	1.3557 (0.0044)		
C7	1.4344 (0.0041)	122.81 (0.27)	
H8	0.9300	118.60	118.60
	C8 -	C9	C7

C9 -	Distance	Angles
C8	1.3557 (0.0044)	
C6	1.4455 (0.0041)	119.43 (0.27)

C17 1.4932 (0.0042) 120.93 (0.27) 119.64 (0.28)

C9 - C8 C6

C10 - Distance Angles

C11 1.3917 (0.0041)

C15 1.3942 (0.0042) 119.76 (0.27)

S1 1.7724 (0.0030) 119.42 (0.23) 120.82 (0.23)

C10 - C11 C15

C11 - Distance Angles

C12 1.3850 (0.0043)

C10 1.3917 (0.0041) 119.55 (0.29)

H11 0.9300 120.23 120.23

C11 - C12 C10

C12 - Distance Angles

C11 1.3850 (0.0043)

C13 1.3916 (0.0045) 121.29 (0.29)

H12 0.9300 119.35 119.35

C12 - C11 C13

C13 - Distance Angles

C14 1.3884 (0.0047)

C12 1.3916 (0.0045) 118.47 (0.28)

C16 1.5095 (0.0043) 120.89 (0.30) 120.60 (0.30)

C13 - C14 C12

C14 -	Distance	Angles	
C15	1.3839 (0.0045)		
C13	1.3884 (0.0047)	121.09 (0.29)	
H14	0.9300	119.46	119.46
	C14 -	C15	C13

C15 -	Distance	Angles	
C14	1.3839 (0.0045)		
C10	1.3942 (0.0042)	119.81 (0.29)	
H15	0.9300	120.09	120.09
	C15 -	C14	C10

C16 -	Distance	Angles		
C13	1.5095 (0.0043)			
H16A	0.9600	109.47		
H16B	0.9600	109.47	109.47	
H16C	0.9600	109.47	109.47	109.47
	C16 -	C13	H16A	H16B

C17 -	Distance	Angles		
C9	1.4932 (0.0042)			
H17A	0.9600	109.47		
H17B	0.9600	109.47	109.47	
H17C	0.9600	109.47	109.47	109.47
	C17 -	C9	H17A	H17B

N1 - Distance Angles

C7 1.3571 (0.0036)

C5 1.3857 (0.0040) 124.00 (0.26)

H1A 0.8773 (0.0349) 116.49 (2.19) 119.49 (2.20)

N1 - C7 C5

N2 - Distance Angles

C7 1.3368 (0.0039)

S1 1.5904 (0.0025) 123.05 (0.21)

N2 - C7

O1 - Distance Angles

S1 1.4472 (0.0021)

O1 -

O2 - Distance Angles

S1 1.4423 (0.0023)

O2 -

S1 - Distance Angles

O2 1.4423 (0.0023)

O1 1.4472 (0.0022) 116.36 (0.13)

N2 1.5904 (0.0025) 104.60 (0.13) 114.45 (0.13)

C10 1.7724 (0.0030) 106.03 (0.14) 107.03 (0.13) 107.82 (0.14)

S1 - O2 O1 N2

Selected torsion angles

-0.12 (0.44) C6 - C1 - C2 - C3

0.05 (0.44) C1 - C2 - C3 - C4

0.21 (0.43) C2 - C3 - C4 - C5

179.84 (0.25) C3 - C4 - C5 - N1

-0.40 (0.42) C3 - C4 - C5 - C6

-0.07 (0.42) C2 - C1 - C6 - C5

-179.42 (0.28) C2 - C1 - C6 - C9

-179.91 (0.25) N1 - C5 - C6 - C1

0.33 (0.41) C4 - C5 - C6 - C1

-0.52 (0.39) N1 - C5 - C6 - C9

179.71 (0.26) C4 - C5 - C6 - C9

-177.23 (0.28) N2 - C7 - C8 - C9

1.54 (0.41) N1 - C7 - C8 - C9

-2.22 (0.43) C7 - C8 - C9 - C6

178.08 (0.27) C7 - C8 - C9 - C17

-178.99 (0.27) C1 - C6 - C9 - C8

1.66 (0.40) C5 - C6 - C9 - C8

0.72 (0.42) C1 - C6 - C9 - C17

-178.63 (0.26) C5 - C6 - C9 - C17

-0.35 (0.45) C15 - C10 - C11 - C12

179.26 (0.23) S1 - C10 - C11 - C12

0.25 (0.47) C10 - C11 - C12 - C13

0.78 (0.47) C11 - C12 - C13 - C14

-176.94 (0.29) C11 - C12 - C13 - C16

-1.73 (0.47) C12 - C13 - C14 - C15

175.98 (0.31) C16 - C13 - C14 - C15

1.64 (0.48) C13 - C14 - C15 - C10

-0.58 (0.46) C11 - C10 - C15 - C14

179.81 (0.24) S1 - C10 - C15 - C14

178.61 (0.24) N2 - C7 - N1 - C5

-0.33 (0.40) C8 - C7 - N1 - C5

179.65 (0.26) C4 - C5 - N1 - C7

-0.11 (0.41) C6 - C5 - N1 - C7

176.92 (0.20) N1 - C7 - N2 - S1

-4.30 (0.43) C8 - C7 - N2 - S1

-170.78 (0.23) C7 - N2 - S1 - O2

-42.28 (0.28) C7 - N2 - S1 - O1

76.67 (0.26) C7 - N2 - S1 - C10

-36.54 (0.28) C11 - C10 - S1 - O2

143.07 (0.25) C15 - C10 - S1 - O2

-161.37 (0.24) C11 - C10 - S1 - O1

18.24 (0.29) C15 - C10 - S1 - O1

75.05 (0.27) C11 - C10 - S1 - N2

-105.34 (0.26) C15 - C10 - S1 - N2
